# Vinylogous Electrochemical Carboxylation of Dienones

**DOI:** 10.1021/acselectrochem.5c00078

**Published:** 2025-07-03

**Authors:** Catia Nicoletti, Elena Tacchi, Noemi Trovato, Manuel Orlandi, Luca Dell’Amico, Abdirisak Ahmed Isse, Marco Fantin, Andrea Sartorel

**Affiliations:** Department of Chemical Sciences, 9308 University of Padova, via Marzolo 1 35131 Padova, Italy

**Keywords:** carbon dioxide, electrochemical carboxylation, unsaturated systems, vinylogous reactivity, organic electrosynthesis

## Abstract

Among the reconversion strategies of carbon dioxide, its electrochemical fixation as a C1 synthon onto organic scaffolds (electrochemical carboxylation) displays an enormous synthetic potential and is thus receiving increasing attention. Examples of electrochemical carboxylation are reported via the activation of C–X (X = halide or pseudo-halide), C–H, or C–C bonds, or of unsaturated systems comprising C=C, C=N, and C=O bonds. In this work, we report the electrochemical carboxylation of dienones, achieving the synthesis of 6-oxo-carboxylic acid derivatives in useful yields up to 56%. We show that electrochemical reduction of dienones drives their umpolung reactivity as nucleophiles toward carbon dioxide, promoting a δ-selective electrochemical carboxylation. The electrochemical reactivity was expanded to polyconjugated carboxylic derivatives such as α,β,γ,δ-unsaturated esters, thioesters, and amides. This work provides to the best of our knowledge the first example of vinylogous electrochemical reactivity in extended conjugated carbonyls involving carbon dioxide as the partner reactant. The reactivity and regioselectivity are rationalized through a mechanistic investigation that integrates cyclic voltammetry analysis and DFT calculations: this supports the reactivity with the CO_2_ electrophile of nucleophilic doubly reduced species of the parent compound, preferentially occurring at the vinylogous position. The role of CO_2_ in this process is also discussed. Considering the large synthetic versatility of carboxylic acids, our new protocol may become a useful tool for accessing novel synthons in drug design and general scientific development. We believe that these results will provide a guide for future studies on CO_2_ fixation.

## Graphical abstract

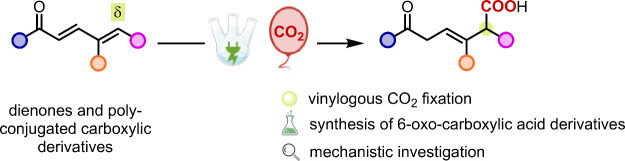

## Introduction

Carbon dioxide is the byproduct of many anthropogenic activities involving combustion of fossil fuels, and its release and increase of concentration in the atmosphere (currently reaching around 420 ppm) induce the greenhouse effect and the global temperature increase associated with the severe climate changes that humanity is facing.^1,2^ Capture and reconversion of CO_2_ are thus objectives of primary interest to reach carbon neutrality;^2^ electrochemistry can promote the development of valid technologies for these processes, since production of electricity can be sustained through the use of renewable energy sources.^3,4^

Among these opportunities, CO_2_ can serve as a cheap, abundant, nontoxic, and renewable one-carbon building block to be exploited in organic synthesis for the formation of new C–C bonds via carboxylation processes.^5–12^ In particular, electrochemical carboxylation processes are versatile and enable selective functionalization of organic scaffolds, providing access under mild conditions to a variety of carboxylic acid derivatives relevant to the pharmaceutical and chemical industries.^13^ Indeed, electricity offers an alternative means of fixing carbon dioxide, which would otherwise require highly reactive reagents and/or harsh conditions to overcome its thermodynamic stability and kinetic inertia.^5,14^ Notably, cascade electrochemical transformations such as CO_2_-to-CO conversion followed by carbonylation have also been demonstrated.^15^

Electrochemical carboxylation typically proceeds through the cathodic generation of carbanion intermediates from reduction of the parent organic substrates with these nucleophilic species that subsequently react with CO_2_ in the carboxylation step. This route is generally more favorable compared to the electrochemical generation of nucleophilic CO_2_^•–^ radical anion, which requires significantly more negative potentials (i.e. −2.2 to −2.4 V vs SCE in organic solvents,^16,17^ depending on the working electrode).

The electrochemical strategy was implemented towards the carboxylation of aryl C–H bonds;^18–20^ other organic substrates that have been considered are aryl/benzyl halides or pseudo-halides^21,22^ and unsaturated scaffolds. Examples of these latter include olefins,^23–25^ imines (for the synthesis of α-amino acids),^26,27^ diynes,^28^ α,β-unsaturated ketones,^29–32^ esters and amides,^33^ allyl chlorides^34^ and esters,^35^ dienes,^36–38^ skipped dienes,^39^ and allenes^17^ (Scheme 1).

**Scheme 1:**
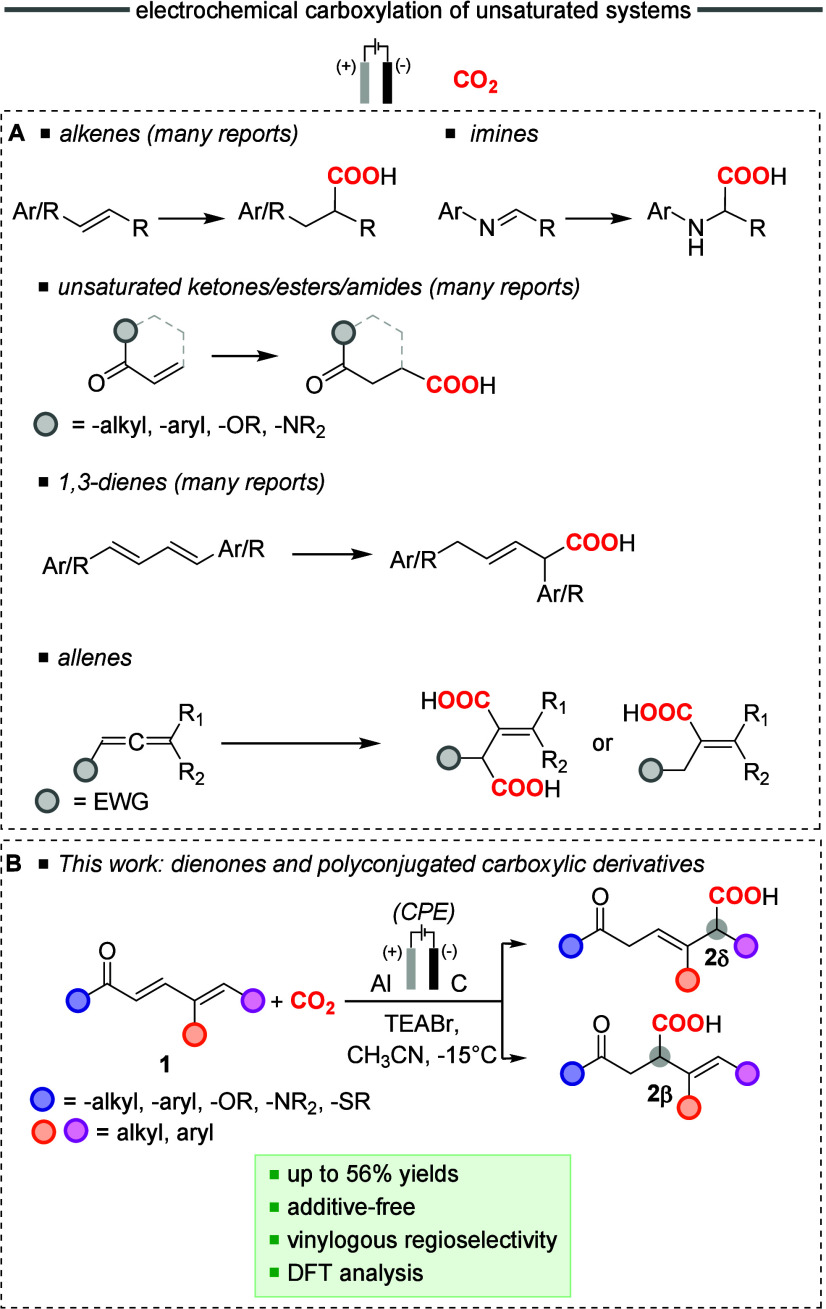
Panel A: Recent Examples of Electrochemical Carbon Dioxide Fixation onto Unsaturated Organic Scaffolds and Panel B: Electrochemical Carboxylation of Dienones (α,β,γ,δ-Unsaturated Ketones) and Polyconjugated Carboxylic Derivatives (α,β,γ,δ-Unsaturated Esters, Thioesters, and Amides) Presented in This Work, Combined with a Cyclic Voltammetry and Theoretical Analysis to Rationalize the Regioselectivity and Supporting the Reaction Mechanism

Among these, extended conjugated substrates have been limitedly explored. In this work, we report the carboxylation of dienones (Scheme 1). Classically, dienones react as Michael acceptors at multiple sites of the carbon chain producing different regioisomers.^40–42^ Here, we show that their electrochemical reduction drives an umpolung reactivity as nucleophiles toward carbon dioxide, promoting a δ-selective carboxylation. To the best of our knowledge this is the first example of vinylogous^43,44^ electrochemical reactivity of dienones involving carbon dioxide as the electrophilic partner reactant (Scheme 1). The protocol was extended also to polyconjugated carboxylic derivatives such as α,β,γ,δ-unsaturated esters, thioesters, and amides. The inherent reactivity and selectivity of the process are rationalized by combining digital simulation of cyclic voltammetry with DFT calculations that dissect the thermodynamic and kinetic parameters of the carboxylation step.^45^ These results provide insightful perspectives that can be extended to other organic intermediates and guide future studies of CO_2_ conversion and fixation.

## Results and discussion

### Electrochemical Carboxylation of Dienones

1,5-Diphenylpenta-2,4-dien-1-one (**1a**, see Supplementary Information for the synthesis^46^) was used as a model compound in the electrochemical carboxylation. The cyclic voltammetry (CV) of **1a** towards cathodic scan under argon reveals the presence of two one-electron cathodic processes, similarly to the case of α,β-unsaturated ketones, such as chalcone (Figure S1 in Supporting Information Section C, see also Figures S2–S18). Accordingly, the first wave is reversible at a high scan rate with an *E*_1/2_ = −1.74 V vs Fc^+^/Fc and is attributed to the reduction of **1a** to the radical anion **1a** ^**•–**^; the second irreversible reduction process is characterized by a cathodic peak potential of −2.30 V vs Fc^+^/Fc (recorded via CV at 0.2 V s^–1^), attributed to the formation of the dianion **1a** ^**2–**^. The CV analysis both in the absence and in the presence of CO_2_ will be extensively discussed in the mechanism section (*vide infra*).

The determination of the reduction potentials of **1a** was pivotal in setting controlled potential electrolysis (CPE) conditions for generating the reduced forms of **1a** and to further reach their reactivity towards CO_2_.^30^ The optimized setup includes an undivided cell with a glassy carbon rod, an aluminum wire, and a Ag/AgCl (NaCl 3 M) used as working, counter, and reference electrodes, respectively. The use of Al anode avoids the addition of sacrificial donor species in solution to be oxidized at the anodic side,^23,33^ although with the drawback of disposal of Al­(III) species at the end of the process. Its use allowed us to reach the highest carboxylation yield in the present system (*vide infra*); stabilization of carboxylate anion products and negatively charged reaction intermediates by Al­(III) ions was reported in the literature.^47^ The electrocarboxylation processes were generally carried out on a millimolar scale, using **1** (0.3 mmol) in a 15 mL solution. Prior to applying the cell potential, CO_2_ (1 atm) was bubbled in the reaction mixture and continuously maintained throughout the electrosynthetic procedure (see Supporting Information Sections E–F, Figures S23–S24).

Under the optimized conditions, application of a −2.46 V vs Fc^+^/Fc potential at −15°C led to initial electrolysis current of 15 mA, with accumulation of 2 F/mol_**1a**_ charge in ca 2 h before observing a significant >90% current drop (entry 1 in Table 1; for additional details, see Table S1 in Supporting Information, Section E). Acidic work-up led to the obtainment of 6-oxo and 4-oxo carboxylic acids **2aδ** and **2aβ** in an overall useful 48–55% yield (the yield range results from three independent experiments), with carboxylation occurring in δ (i.e. at C-5 of the dienone, yielding **2aδ** as the major product) and β positions (i.e. at C-3 of the dienone, yielding **2aβ** as the minor product) with respect to the carbonyl group, with a 10:1 **2aδ**:**2aβ** regioselectivity, entry 1 in Table 1.

**Table 1:**
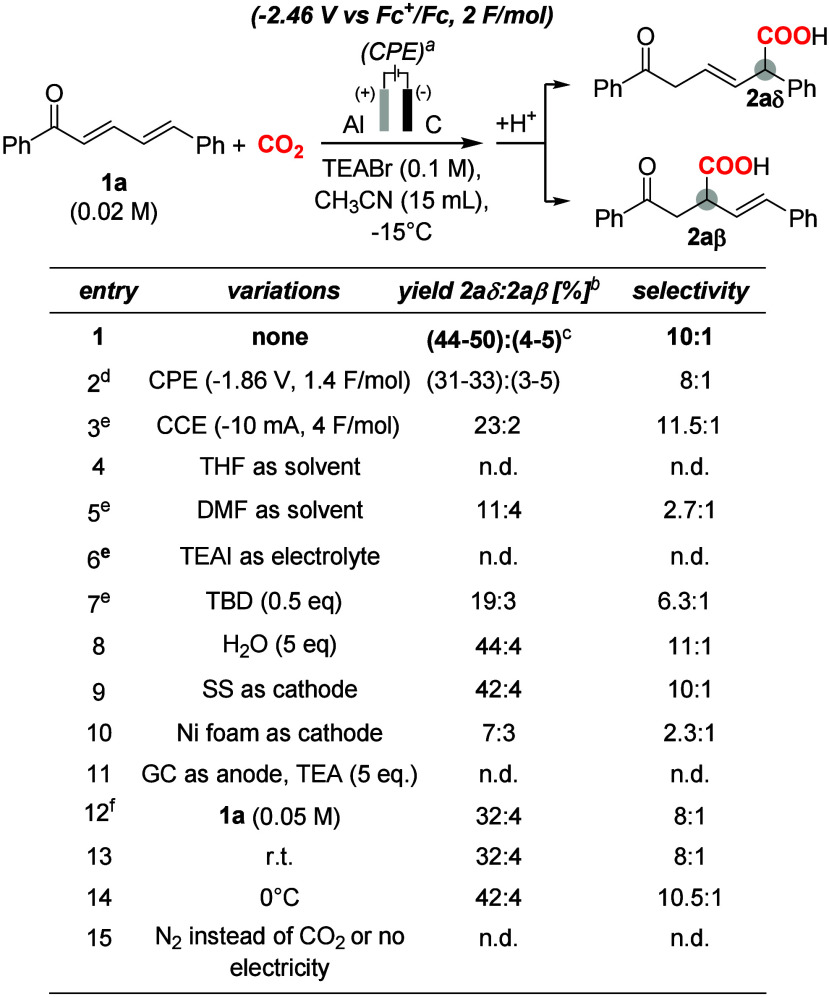
Electrochemical Carboxylation of **1a**: Optimization of the Reaction Conditions

a Reaction conditions: GC cathode, Al anode, Ag/AgCl (3 M NaCl) reference electrode separated by a frit, **1a** (0.02 M), CH_3_CN (15 mL) saturated with CO_2_ (1 atm), TEABr (0.1 M) as electrolyte, NaCl–ice bath at −15°C, CPE at −2 V vs Ag/AgCl (−2.46 V vs Fc^+^/Fc), 2 F/mol.

b Determined by ^1^H-NMR with CH_2_Br_2_ as internal standard after acidification (1 M HCl) and extraction (EtOAc).

c Range of yields obtained from repeated experiments using TEABr or TBABr as electrolytes.

d Range of yields obtained from repeated experiments. In these cases, after reaching ca. 1.4 F/mol_**1a**_, no significant charge is further accumulated.

e Reaction conducted at 0 °C.

f In this case, after reaching ca 1.6 F/mol_**1a**_, no significant charge is further accumulated. GC = Glassy Carbon. SS = stainless steel. CPE = controlled potential electrolysis. CCE = controlled current electrolysis. TEABr = Et_4_NBr. DMF = *N*,*N*-dimethylformamide. THF = tetrahydrofuran. TBD = 1,5,7-triazabicyclo[4.4.0]­dec-5-ene. TEAI = Et_4_NI. TEOA = triethanolamine. TEA = triethylamine. n.d. = not detected. Complete conditions are reported in the Supporting Information.

The nature of the carboxylation products was confirmed by mass spectrometry analysis and multiple NMR techniques on the isolated products; see the Supporting Information, Sections G and K. It is worth noting that the yield of carboxylation of **1a** is significantly lower with respect to the one observed in the same conditions for chalcone^48^ (74% yield) as an example of α,β-unsaturated ketones; this is likely ascribable to competitive pathways involving the electrogenerated reduced species of **1a** (*vide infra*).

The optimization of the electrosynthetic conditions included the evaluation of the following variables (Table 1):

(a) Potentiostatic *vs*. galvanostatic conditions, entries 1–3: higher yields of **2aδ** and **2aβ** were obtained by employing potentiostatic conditions in 3-electrode electrochemical configuration (entries 1–2 in Table 1) with respect to 2-electrode electrochemical configuration (CCE at 10 mA, entry 3, Table 1, see Table S1 in Supporting Information for further discussion on these conditions). Concerning potentiostatic conditions, a lower yield is found operating at −1.86 V corresponding to the one electron reduction of **1a** (complete substrate conversion and 33% yield of **2aδ**, 1.4 F/mol_1a_ charge, entry 2) with respect to the one registered at −2.46 V corresponding to the two-electron reduction of **1a** (48–55% yield, 2 F/mol_1a_ charge, entry 1). This trend and the reactivity of **1a** ^•–^ and **1a** ^2–^ towards CO_2_ will be further discussed in the mechanism session (*vide infra*).
(b) Solvent, entries 1, 4–5: a higher yield was obtained in acetonitrile (entry 1) with respect to THF or DMF (entries 4–5 in Table 1), typical solvents employed in carboxylation and providing good solubility of CO_2_.^49^
(c) Electrolyte, entries 1 and 6: the use of TEAI^23^ instead of TEABr lead to a complete abatement of the yield for the carboxylation of **1a**; its role was not further investigated. Moreover, a tetraalkylammonium electrolyte with a bromide anion is necessary to avoid passivation of the anode and consequent potential drop, that were promptly observed in our conditions with TBAPF_6_ and TBABF_4_ electrolytes. The passivation is due to insoluble Al­(III) salts being deposited at the Al surface;^50^ the role of bromide anion is likely to stabilize the released Al­(III) ions, avoiding their precipitation at the anode.
(d) Additives and water content, entries 1 and 7–8: the use of 1,5,7-triazabicyclo[4.4.0]­dec-5-ene (TBD) was considered because it was demonstrated that TBD could form a zwitterionic adduct with CO_2_ in CH_3_CN^51^ and could increase the CO_2_ availability in the reaction conditions. In the case of the carboxylation of **1a**, the use of TBD led to an abatement of the reaction yield (compare entries 1 and 7 in Table 1). Interestingly, the system exhibited a good tolerance towards the addition of water, which can be an adventitious contaminant of electrolytes and solvents: in particular, the presence of up to 5 equiv of water with respect to substrate **1a** did not affect the yield of **2aδ** and the **δ**/**β** selectivity (compare entries 1 and 8 in Table 1).
(e) Working electrode, entries 1, 9–10: a stainless steel (SS) rod and Ni foam cathode were employed, since they were previously considered in carboxylation of dienes.^36,39^ While SS led to a slightly reduced yield with respect to GC, the use of a Ni foam was associated with an abatement of carboxylation yield (compare entry 1 and entries 9–10 in Table 1).
(f) Anodic process, entries 1 and 11: the utilization of a GC electrode in the presence of triethanolamine or triethylamine as electron donor was considered for the replacement of the Al sacrificial anode; however, no carboxylation was observed under these conditions (entry 11 in Table 1). Replacing Al with SS or Zn anodes enabled the carboxylation, although with a decrease of the overall yield (see entries 15 and 16 in Table S1).
(g) Increasing the substrate amount to 0.75 mmol led to a decrease of the overall yield (entry 12, yield 36%), likely ascribable to the favored occurrence of the competitive bimolecular processes.
(h) Reaction temperature, entries 1 and 13–14 (Table S1 in Supporting Information for additional entries): reaction temperature is associated with the solubility of CO_2_.^49^ The temperature was varied between −41 °C and +58 °C and was shown to impact slightly on the carboxylation yield but mostly on the **δ**/**β** selectivity.

Control tests confirmed the necessity of CO_2_ and electricity to obtain the carboxylation of **1a** (entry 15 in Table 1).

With the optimal conditions in hand, we explored the generality of the process (Scheme 2) considering the carboxylation of **1­(a–q)**. In particular, concerning the group bound to the carbonyl moiety (blue group in Scheme 2) the protocol was tolerant towards the introduction of electron-donating or mild electron-withdrawing groups on the phenyl ring (**2b**, **2c**, and **2d**), although suffering from a decrease of the yield in the case of the electron-withdrawing substituent (total yield of 44 and 25% for **2b** and **2c**, respectively, and δ/β regioselectivity of 8:1 and >15:1 for **2b** and **2c**, respectively; when introducing a −CN in para position to the phenyl ring, as in the case of substrate **1n**, no electrochemical carboxylation was observed). In the case of **1c**, The reduced yield with the −CF_3_ group may also be ascribed to cathodic defluorination, although it is expected to occur at very negative potentials.^52^ Utilization of different aromatic substituents such as naphthyl- or furyl- groups was also tolerated (**2d** and **2e**, with total yield 50% in both cases and δ/β regioselectivity of >15:1 and 4:1 for **2d** and **2e**, respectively). Replacement of the aromatic groups with an aliphatic one was also considered, extending the electrosynthesis to **2f** (overall yield 56%, δ/β regioselectivity > 15).

**Scheme 2:**
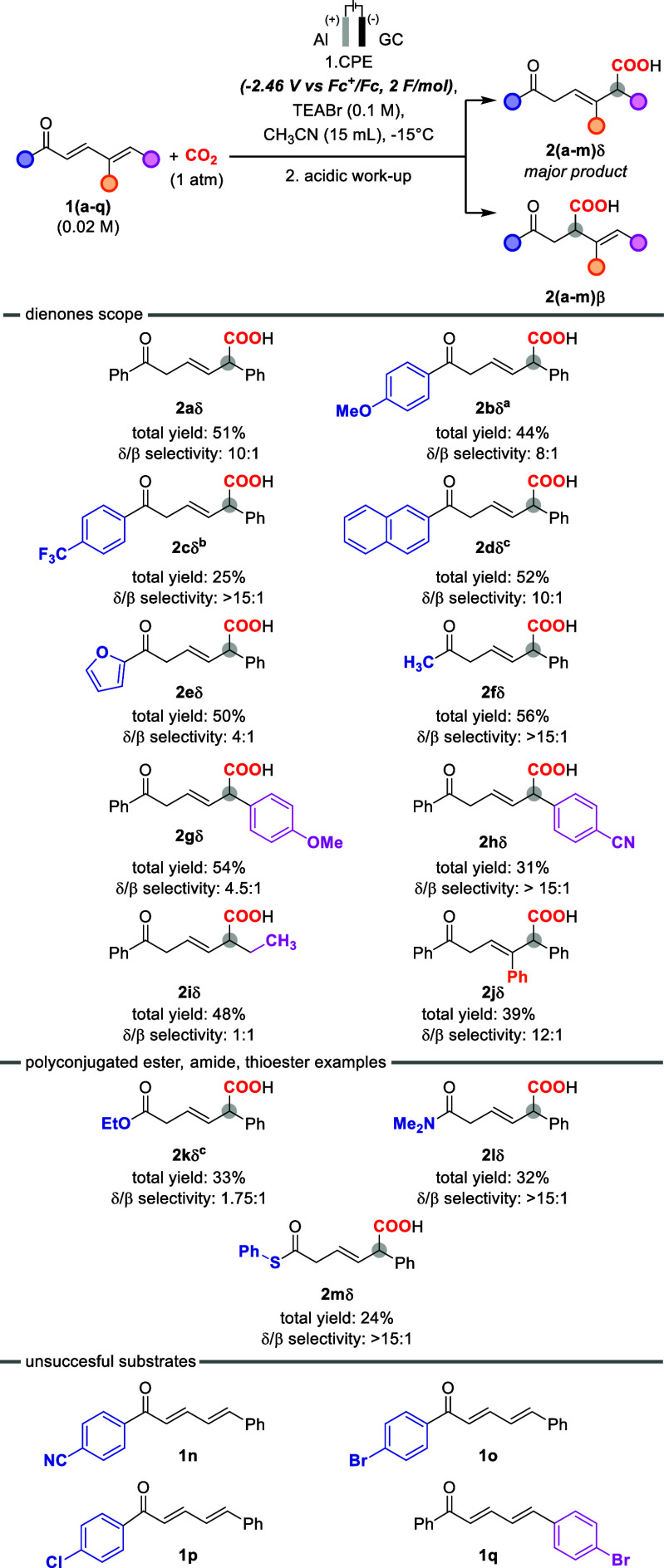
Examples of Carboxylation of Dienones and Polyconjugated Carboxylic Derivatives (α,β,γ,δ-Unsaturated Esters, Thioesters, and Amides) Obtained with the Electrochemical Carboxylation Protocol Presented in This Work^a^

a Reaction performed at −2.66 V vs Fc^+^/Fc.
b Reaction performed at −2.26 V vs Fc^+^/Fc.
c Reaction performed at −2.36 V vs Fc^+^/Fc.
d Only **2δ** regioisomers are represented; **2β** regioisomers were isolated when the yield was >10%, i.e., for **2eβ**, **2gβ**, **2iβ**, and **2kβ**.

Regarding the changes on the -ene moiety (purple ball in Scheme 2), the reaction was tolerant to the installation of an electron-donating or an electron-withdrawing substituent on the phenyl ring, as in the case of **2g** and **2h**, although also in this case a decreased yield was observed with −CN electron-withdrawing group (total yield of 54 and 31% for **2g** and **2h**, respectively, and δ/β regioselectivity of 4.5:1 and >15:1 for **2g** and **2h**, respectively). Replacement of the aromatic group with an aliphatic one was considered also in this case, extending the electrosynthesis to **2i**, which represents the only case in which the two regioisomers are obtained in a similar ratio (overall yield 48%, δ/β regioselectivity 1:1).

Installation of an additional phenyl ring in the γ-position was also tolerated, obtaining **2j** in overall 39% yield and 12:1 δ/β regioselectivity.

The protocol was then expanded to polyconjugated esters, amides, and thioesters, although with a general decrease of the reaction yields, but still maintaining a regioselectivity for the δ-isomer (**2k**, **2l**, and **2m**, with overall yields of 33%, 32%, and 24%, respectively, and δ/β selectivity of 1.75:1, >15:1, and >15:1, respectively).

Finally, the electrochemical carboxylation was unsuccessful in the case of dienones with phenyl rings bearing -Br and -Cl substituents, as in the case of **1o**, **1p**, and **1q**. In these cases, poor solubility in acetonitrile of the starting compound **1­(o–q)** was observed. The reaction was conducted also at r.t., in DMF, or with a DMF:ACN mixed solvent but a rapid current and potential drop during the electrolysis and a clearly visible passivation of the GC working electrode occurring in all cases. It is worth mentioning that the electrochemical reduction of **1o** and **1q** starting compounds may be associated with elimination of a Br^–^ anion,^53^ which could contribute to the generation of competitive pathways, such as ring carboxylated products. In our case, multiple-irreversible reduction waves were observed in the CV of **1o** and **1q** following the initial single-reduction wave, accompanied by a significant increase in current intensity (see Supporting Information for more details, Figures S16–S18). This reduction of undesired intermediates/products can be produced during the electrochemical carboxylation, as their formation is associated with a reduction potential close to the CPE applied potential of −2.46 V.

### Mechanism Analysis, CV Digital Simulations, δ/β Selectivity, and DFT Calculations

We sought to rationalize the electrochemical reactivity and the observed regioselectivity in the carboxylation of dienones by combining cyclic voltammetry analysis and DFT calculations (ωb97XD/aug-cc-pvtz//ωb97XD/def2tzvp level of theory as in our previous work,^45^ including a continuum model for acetonitrile solvent).

As previously anticipated, the CV of the **1a** model compound shows two cathodic waves due to generation of **1a** ^**•–**^ and **1a** ^**2–**^. In argon saturated acetonitrile at −15 °C, the reversibility of the first wave (reduction of **1a** to **1a** ^**•–**^, *E*_1/2_ = −1.74 V vs Fc^+^/Fc, step 1 in Figure 1) is observed only at high scan rates, indicative of a reactivity of **1a** ^**•–**^. Electrolysis in these conditions consumed ca. 0.9 F/mol_1a_ and led to a mixture of at least three dimers (see Supporting Information, Section H, Figure S22); dimerization of **1a** ^**•–**^ (step 2 in Figure 1) provides an analogy of what observed for α,β-unsaturated carbonyls.^54^ The residual fraction of **1a** ^**•–**^ undergoes reduction to **1a** ^**2–**^ (step 3 in Figure 1). Digital simulations of the CV at different scan rates according to steps 1–3 (Figure 1a) lead to a very good match with experimental traces and provide a rate constant *k* = 1.3 × 10^5^ M^–1^ s^–1^ for the dimerization step (Figure 1a, and Supporting Information, Section I). This rate constant for **1a** ^**•–**^ dimerization is consistent with the value of *k* = 1.17 × 10^5^ M^–1^ s^–1^, obtained from the analysis of the dependence of the cathodic peak potential (*E*_pc1_) versus the logarithm of the scan rate in CV (see Supporting Information, Figure S27 and related discussion).

**Figure 1:**
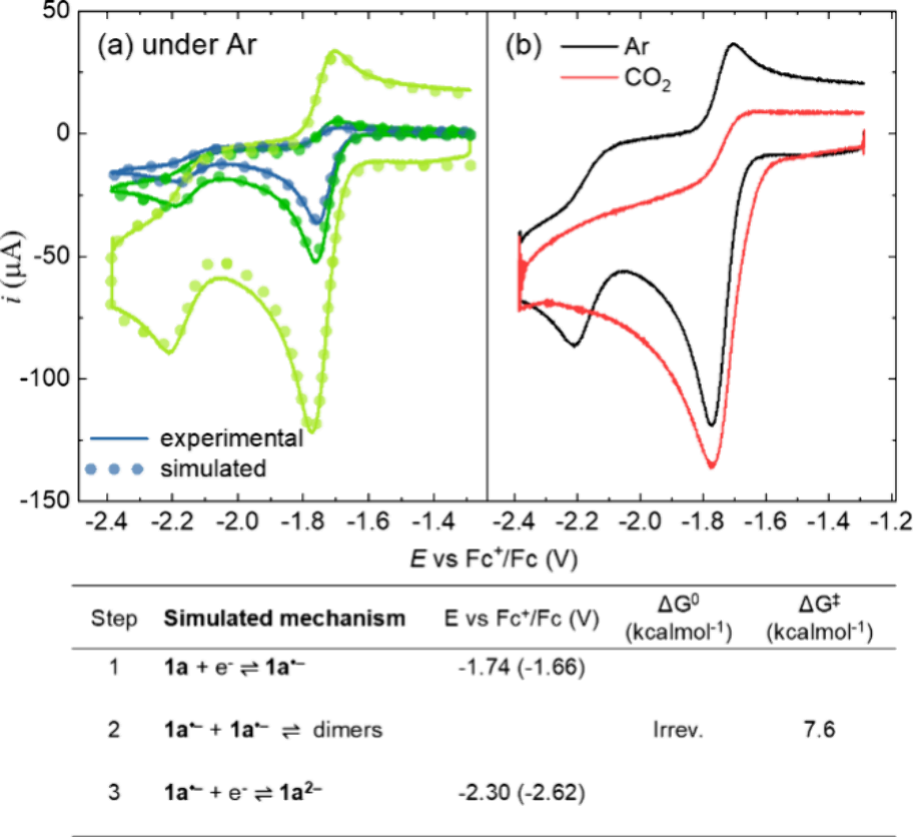
(a) Experimental (lines) and simulated (dots) voltammograms of **1a** recorded under Ar at scan rates = 1, 2, and 10 V s^–1^. CVs were recorded on 0.5 mM solutions of **1a** in CH_3_CN + 0.1 M *n*Bu_4_NPF_6_ at −15 °C on a glassy carbon disk (*d* = 3 mm). (b) Comparison between CVs recorded under Ar or CO_2_ at scan rate = 10 V s^–1^. The bottom part of the figure reports the mechanistic steps used to simulate the CV, with the associated fitting parameters (the values reported in parentheses refer to DFT calculations).

Under CO_2_, the voltammogram (Figure 1b) shows significant changes compared to the trace under argon: (i) the foot of the wave is anticipated at a less negative potential, from −1.65 to −1.60 V; (ii) an enhanced cathodic current is observed at and after the first reduction wave; and (iii) the second reduction wave disappears, all indicative of reactivity of **1a** ^**•–**^ with CO_2_.

The reactivity of **1a** ^**•–**^ with CO_2_ was investigated by DFT calculations. From the computed values of free energies of optimized **1a** and **1a** ^**•–**^, a calculated potential of −1.66 V vs Fc^+^/Fc was obtained for the **1a**/**1a** ^**•–**^ couple, in excellent agreement with the experimental value (Supporting Information, Section A). Reactivity of **1a** ^**•–**^ with CO_2_ was considered at carbon centers in β and δ positions and at the oxygen atom of the carbonyl.^55,56^ The most favorable reactivity is found in this last case, with the formation of a new C–O bond between the C of CO_2_ and the O of the carbonyl, leading to **3a** ^**•–**^ (Δ*G* ^0^ = +5.0 and Δ*G* ^‡^ = 10.0 kcal mol^–1^), Scheme 3.^57^ Interestingly, further reduction of **3a** ^**•–**^ to **3a** ^**2–**^ requires a potential of −1.80 V vs Fc^+^/Fc, ca. 0.8 V more positive than the one of −2.62 V calculated for reduction of **1a** ^**•–**^ to **1a** ^**2–**^ (Scheme 3). This scenario suggests a role of CO_2_ as a Lewis acid,^55,56^ enabling the formation of the doubly reduced **3a** ^**2–**^ at relatively mild potentials. Indeed, **3a** ^**2–**^ may undergo further reactivity with CO_2_ at both δ and β carbon positions to give **4aδ** ^**2–**^ and **4aβ** ^**2–**^ (Figure 2 and reaction pathway a in Scheme 3), associated to Δ*G* ^0^ values of −9.8 and −8.8 kcal mol^–1^, respectively, and to Δ*G* ^‡^ of 10.7 and 11.3 kcal mol^–1^, respectively (Figure 2).

**Scheme 3:**
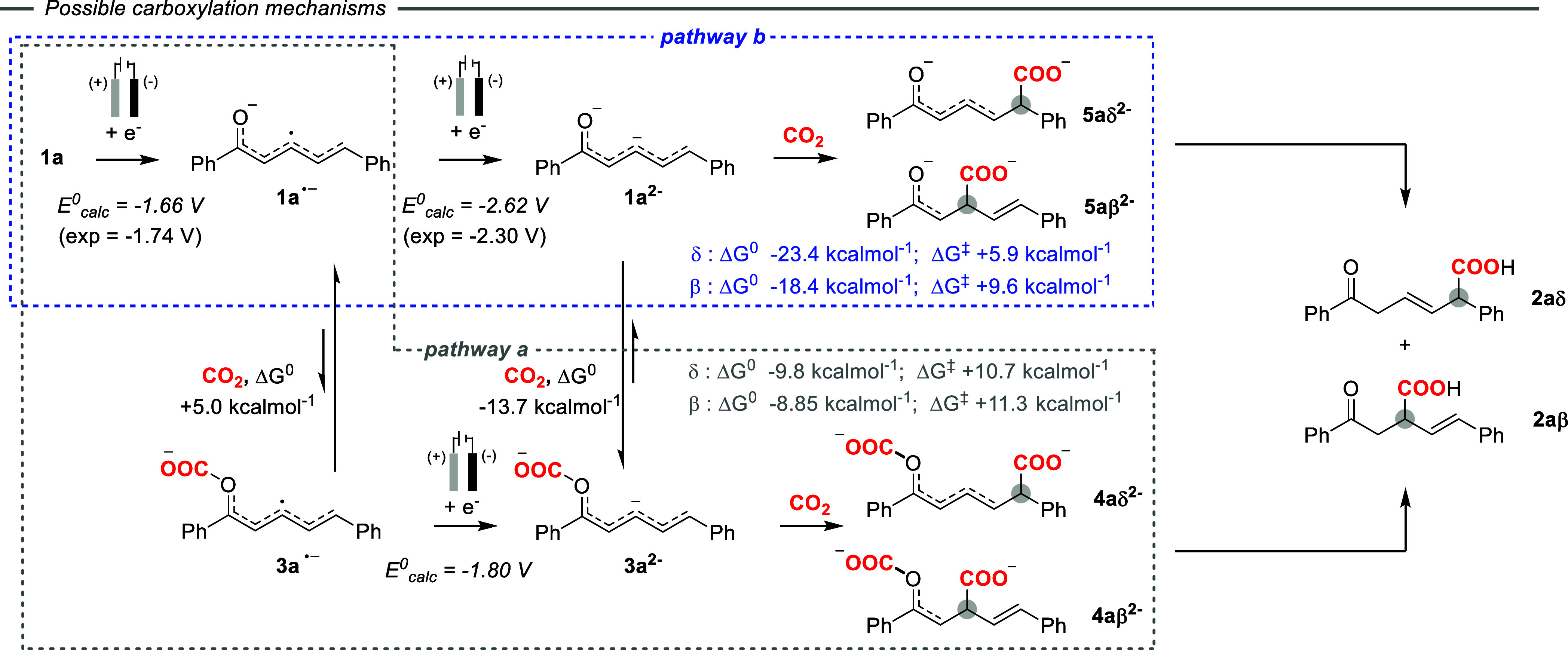
Carboxylation Pathways for **1a**, Including DFT Calculated Parameters^a^

a Potentials are referenced to the Fc^+^/Fc couple.

**Figure 2:**
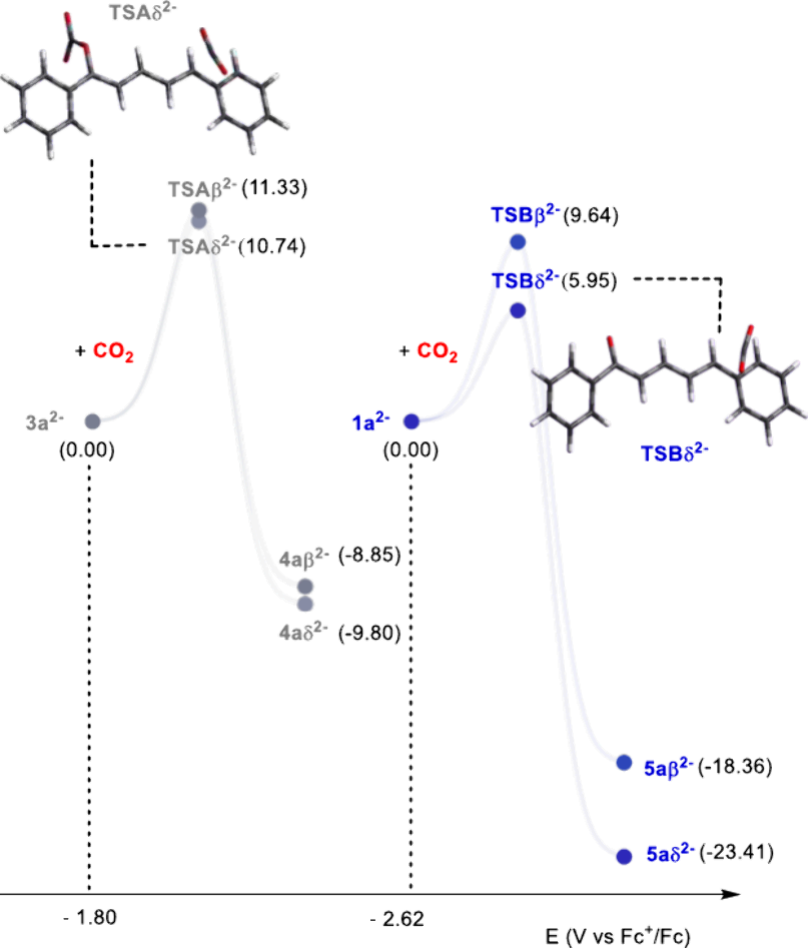
Calculated carboxylation pathways from **3a** ^2–^ and from **1a** ^2–^ at both the β and δ positions.

These steps, together with the residual dimerization of **1a** ^**•–**^ (see above discussion), were considered in a digital simulation of the cyclic voltammetry under CO_2_, providing excellent fittings of the experimental traces (Figure 3, steps 1–6). In particular, fitting of the CV at different scan rates supports the weak but fast equilibrium involving **1a** ^**•–**^ and CO_2_ to give **3a** ^**•–**^ (step 4 in Figure 3, best fitting obtained with Δ*G* ^0^ = +0.9 and Δ*G* ^‡^ = 8.6 kcal mol^–1^ in good agreement with DFT calculated values), and the further reduction of **3a** ^**•–**^ to **3a** ^**2–**^, undergoing irreversible reactivity (steps 5–6 in Figure 3, refer to the Supporting Information Section I for the complete simulated mechanism as well for the calculation of thermodynamic and kinetic parameters).

**Figure 3:**
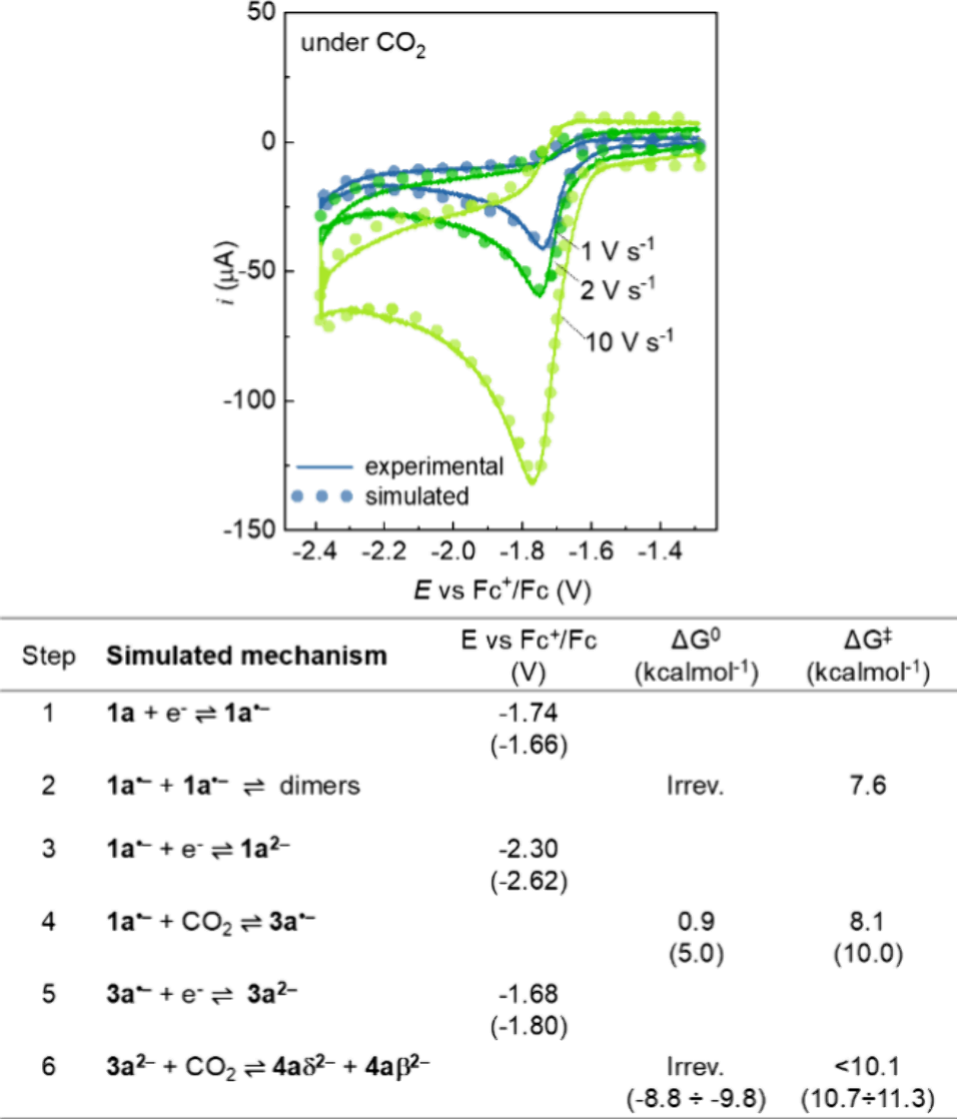
Experimental (lines) and simulated (dots) voltammograms of **1a** recorded under CO_2_. CVs were recorded on 0.5 mM solutions of **1a** in CH_3_CN + 0.1 M *n*Bu_4_NPF_6_ at −15 °C on a glassy carbon disk (*d* = 3 mm). The bottom part of the figure reports the mechanistic steps used to simulate the CV, with the associated fitting parameters (the values reported in brackets refer to DFT calculations).

This reactivity scenario corroborates pathway a in Scheme 3 and is consistent with the 33% carboxylation yield obtained at −1.86 V (entry 6 in Table 1), with the preferential reactivity at the δ position. Incidentally, the 1.4 F/mol_**1a**_ charge passed under these conditions supports the partial, second reduction of **3a** ^**•–**^ to **3a** ^**2–**^, which further undergoes carboxylation. The 1.4 F/mol_**1a**_ charge consumption likely arises from competition between bielectronic reduction leading to carboxylation (2 F/mol_**1a**_) and monoelectronic reduction leading to radical coupling (1 F/mol_**1a**_).

As previously discussed, a higher carboxylation yield is obtained by conducting the electrolysis at −2.46 V vs Fc^+^/Fc (entry 1 in Table 1), associated with the two-electron reduction of **1a** to **1a** ^**2–**^ occurring under electrolysis, with **1a** ^**2–**^ being reactive towards CO_2_. Indeed, calculations confirm a favorable carboxylation of **1a** ^**2–**^ (calculated potential of −2.6 V for the **1a** ^**•–**^/**1a** ^**2–**^ couple) to give **5aδ** ^**2–**^ and **5aβ** ^**2–**^ (Figure 2 and reaction pathway b in Scheme 3), with Δ*G* ^0^ values of −23.4 and −18.4 kcal mol^–1^ for CO_2_ insertion in the δ and β positions, respectively, and associated ΔG^‡^ values of 5.9 and 9.6 kcal mol^–1^, respectively. Both pathways a and b will be productive toward **2aδ** and **2aβ** under synthetic conditions; it is worth highlighting that pathways a and b may be simultaneously operative if fast equilibrium between **1a** ^**2–**^/**3a** ^**2–**^ occurs (Scheme 3).

Finally, we evaluated if the role of CO_2_ in favoring a second reduction of the substrate could be achieved by other Lewis acids, such as AlBr_3_ or trimethylsilyl chloride (TMSCl). In the presence of 1 equiv AlBr_3_, CV show the expected anodic shift of the waves of **1a** (Figures S19–20 in Supporting Information),^58^ although electrolysis was hampered by a current drop due to deposition of insoluble materials at the GC cathode. With 1 equiv of TMSCl with respect to **1a**, electrolysis led to 11% yield in carboxylation (Table S1 in Supporting Information). Therefore, the effect of Lewis acid additives, although advantageous in terms of operating potential, should be properly evaluated given the possibility to promote also undesired reaction pathways.

## Conclusions and Perspectives

We have developed a novel electrochemical protocol to install CO_2_ on the δ position of conjugated dienones, providing an example of vinylogous electrochemical reactivity of extended conjugated systems towards CO_2_; the resulting 6-oxo-carboxylic acid derivatives were obtained in 36–56% yield. A combination of cyclic voltammetry analysis through digital simulations and DFT calculations allowed a mechanism to be defined, where the synthetic carboxylation step involves doubly reduced species of the starting compounds and proceeds with low activation energies (< 12 kcal mol^–1^, consistent with CO_2_ as a powerful electrophile) and preferentially at the δ vinylogous position of the unsaturated scaffold. CO_2_ can also act as a Lewis acid activator of the electrogenerated radical anion, favoring a second reduction and thus enabling carboxylation at mild potentials.

These results provide valuable insights into new synthetic strategies involving inert CO_2_, while elucidating the mechanism by combining electrochemical and theoretical tools. We hope that this work will represent a useful guide for future studies on CO_2_ conversion and fixation onto organic compounds, a research field in large expansion due to its synthetic and sustainability aspects.

## Experimental Section

See Section A in the Supporting Information for the synthesis and characterization of the starting materials **1a**–**1o**.

### Electrochemical Carboxylation of **1a**–**1o** (See the Supporting Information, Section D)

Constant potential electrolysis experiments were performed in a custom-made 4-necked glass cell under stirring conditions, with a glassy carbon rod working electrode (ca. 1.5 cm^2^ of geometric area), an Ag/AgCl (NaCl 3 M) reference—separated from the bulk solution by means of a salt bridge, filled with electrolyte solution—and an Al wire counter electrode (⌀ 1.0 mm). The reaction atmosphere was controlled by securing all the cell’s necks by PTFE O-rings, silicone septa, and screw caps. In the optimized conditions, the substrate (0.3 mmol, 1 equiv) and the supporting electrolyte (tetraethylammonium bromide, 1.5 mmol, 5 equiv) were introduced into the electrochemical cell, that was filled with 15 ml of anhydrous CH_3_CN. The cell was then placed in a NaCl–ice batch at −15°C. After saturating the electrolyte solution with CO_2_ for ca. 20 min, the potential of −2 V vs Ag/AgCl was applied, and the CO_2_ atmosphere was kept above the solutions during the electrolysis experiments. The experiments were stopped at Q = −58 C (2 F/mol), when a current drop of ca. 90% with respect to the initial value was observed. The electrolysis solution was evaporated under vacuum, acidified with 2 mL of HCl (1 M), and extracted with ethyl acetate. The organic phase was dried over MgSO_4_ and then concentrated under vacuum. NMR yield was obtained by adding CH_2_Br_2_ as an internal standard. Carboxylation products were purified by preparative HPLC (H_2_O:acetonitrile gradient).

### DFT Calculations (See Supporting Information, Section A.2)

DFT calculations were performed with Gaussian16 and Gauss View 6. For all molecules, geometry optimizations and frequency calculations were performed to obtain the free energies of the species, using the density functional theory (DFT) long-range-corrected method ωB97XD and the def2tzvp basis set. The self-consistent reaction field (SCRF) was used with DFT energies, optimizations, and frequency calculations to model systems in acetonitrile solution at 298.15 K.

### Characterization (see Supporting Information, Section A)

NMR spectra were recorded on Bruker AVANCE Neo 400 Nanobay equipped with a BBFO-ATM-z grad probehead, Bruker 400 AVANCE III HD equipped with a BBI-z grad probe head 5 mm, and Bruker 500 AVANCE III equipped with a BBI-ATM-z grad probe head 5 mm.

Cyclic voltammetries were recorded employing a three-electrode cell, combining a glassy carbon working electrode (3 mm nominal diameter, 7 mm^2^ geometric area), a platinum electrode as counter electrode, and a silver/silver chloride electrode (Ag/AgCl/3 M NaCl) as reference electrode. Oxygen was removed by saturating the solution with high-purity nitrogen (N_2_), argon (Ar), or carbon dioxide (CO_2_). The glass electrochemical cell was kept closed during the measurements; the headspace of the cell was also degassed to prevent dioxygen contamination. CV experiments were conducted in acetonitrile (CH_3_CN), with 0.1 M tetrabutylammonium hexafluorophosphate (TBAPF_6_) at room temperature, and with a scan rate of 0.1 V s^–1^ unless otherwise noted. All potentials were then converted to a ferrocenium/ferrocene couple (Fc^+^/Fc), using an internal reference system.

High resolution mass spectrometry spectra (10 ppm resolution) were acquired with an electrospray ionization spectrometer Xevo G2-S QTof (Waters) coupled with an Acquity H Class UPLC system (Waters).

## Supplementary Material



## Notes

Synthetic and electrosynthetic procedures, cyclic voltammetries, digital simulation, DFT calculations, NMR and high resolution mass spectrometry analysis, and characterization data and spectra (PDF)

## References

[1] Armstrong
McKay D. I., Staal A., Abrams J. F., Winkelmann R., Sakschewski B., Loriani S., Fetzer I., Cornell S. E., Rockström J., Lenton T. M. (2022). Exceeding 1.5°C Global Warming
Could Trigger Multiple Climate Tipping Points. Science.

[2] Zandalinas S. I., Fritschi F. B., Mittler R. (2021). Global Warming, Climate Change, and
Environmental Pollution: Recipe for a Multifactorial Stress Combination
Disaster. Trends Plant Sci..

[3] Biel-Nielsen T. L., Hatton T. A., Villadsen S. N. B., Jakobsen J. S., Bonde J. L., Spormann A. M., Fosbøl P. L. (2023). L. Electrochemistry-Based
CO_2_ Removal Technologies. ChemSusChem.

[4] Diederichsen K. M., Sharifian R., Kang J. S., Liu Y., Kim S., Gallant B. M., Vermaas D., Hatton T. A. (2022). Electrochemical
Methods for Carbon Dioxide Separations. Nat.
Rev. Methods Prim..

[5] Liu Q., Wu L., Jackstell R., Beller M. (2015). Using Carbon Dioxide as a Building
Block in Organic Synthesis. Nat. Commun..

[6] Song Q. W., Ma R., Liu P., Zhang K., He L. N. (2023). Recent Progress
in CO_2_ Conversion into Organic Chemicals by Molecular Catalysis. Green Chem..

[7] Pimparkar S., Dalvi A. K., Koodan A., Maiti S., Al-Thabaiti S. A., Mokhtar M., Dutta A., Lee Y. R., Maiti D. (2021). Recent Advances
in the Incorporation of CO_2_ for C-H and C-C Bond Functionalization. Green Chem..

[8] Bierbaumer S., Nattermann M., Schulz L., Zschoche R., Erb T. J., Winkler C. K., Tinzl M., Glueck S. M. (2023). Enzymatic Conversion
of CO_2_: From Natural to Artificial Utilization. Chem. Rev..

[9] Hasan M. M. F., Rossi L. M., Debecker D. P., Leonard K. C., Li Z., Makhubela B. C. E., Zhao C., Kleij A. (2021). Can CO_2_ and
Renewable Carbon Be Primary Resources for Sustainable Fuels and Chemicals?. ACS Sustain. Chem. Eng..

[10] Qin Y., Cauwenbergh R., Pradhan S., Maiti R., Franck P., Das S. (2023). Straightforward
Synthesis of Functionalized γ-Lactams Using
Impure CO_2_ Stream as the Carbon Source. Nat. Commun..

[11] Cauwenbergh R., Goyal V., Maiti R., Natte K., Das S. (2022). Challenges
and Recent Advancements in the Transformation of CO_2_ into
Carboxylic Acids: Straightforward Assembly with Homogeneous 3d Metals. Chem. Soc. Rev..

[12] Wang Y., Qian Q., Zhang J., Bediako B. B. A., Wang Z., Liu H., Han B. (2019). Synthesis
of Higher Carboxylic Acids from Ethers, CO_2_ and H_2_. Nat. Commun..

[13] Sahoo P. K., Zhang Y., Das S. (2021). CO_2_-Promoted Reactions:
An Emerging Concept for the Synthesis of Fine Chemicals and Pharmaceuticals. ACS Catal..

[14] Qiao C., Shi W., Brandolese A., Benet-Buchholz J., Escudero-Adán E. C., Kleij A. W. (2022). A Novel
Catalytic Route to Polymerizable Bicyclic Cyclic
Carbonate Monomers from Carbon Dioxide. Angew.
Chemie - Int. Ed..

[15] Sheta A. M., Fernández S., Liu C., Dubed-Bandomo G. C., Lloret-Fillol J. (2024). An Electrocatalytic
Cascade Reaction for the Synthesis
of Ketones Using CO_2_ as a CO Surrogate. Angew. Chemie - Int. Ed..

[16] Lamy E., Nadjo L., Saveant J. M. (1977). Standard
Potential and Kinetic Parameters
of the Electrochemical Reduction of Carbon Dioxide in Dimethylformamide. J. Electroanal Chem..

[17] Brunetti A., Garbini M., Autuori G., Zanardi C., Bertuzzi G., Bandini M. (2024). Electrochemical Synthesis of Itaconic
Acid Derivatives
via Chemodivergent Single and Double Carboxylation of Allenes with
CO_2_. Chem. - A Eur. J..

[18] Zhao Z., Liu Y., Wang S., Tang S., Ma D., Zhu Z., Guo C., Qiu Y. (2023). Site-Selective Electrochemical C-H Carboxylation of
Arenes with CO_2_. Angew. Chem. Int.
Ed..

[19] Rawat V. K., Hayashi H., Katsuyama H., Mangaonkar S. R., Mita T. (2023). Revisiting the Electrochemical Carboxylation of Naphthalene with
CO_2_: Selective Monocarboxylation of 2-Substituted Naphthalenes. Org. Lett..

[20] Sun G. Q., Yu P., Zhang W., Zhang W., Wang Y., Liao L. L., Zhang Z., Li L., Lu Z., Yu D. G., Lin S. (2023). Electrochemical Reactor
Dictates Site Selectivity in N-Heteroarene
Carboxylations. Nature.

[21] Durante C., Isse A. A., Todesco F., Gennaro A. (2013). Electrocatalytic Activation
of Aromatic Carbon-Bromine Bonds toward Carboxylation at Silver and
Copper Cathodes. J. Electrochem. Soc..

[22] Senboku H., Yoneda K., Hara S. (2013). Regioselective Electrochemical Carboxylation
of Polyfluoroarenes. Electrochemistry.

[23] Alkayal A., Tabas V., Montanaro S., Wright I. A., Malkov A. V., Buckley B. R. (2020). Harnessing Applied Potential: Selective β-Hydrocarboxylation
of Substituted Olefins. J. Am. Chem. Soc..

[24] Ballivet-Tkatchenko D., Folest J.-C., Tanji J. (2000). Electrocatalytic
Reduction of CO_2_ for the Selective Carboxylation of Olefins. Appl. Organomet. Chem..

[25] Bringmann J., Dinjus E. (2001). Electrochemical Synthesis
of Carboxylic Acids from
Alkenes Using Various Nickel-Organic Mediators: CO_2_ as
C1-Synthon. Appl. Organomet. Chem..

[26] Naito Y., Nakamura Y., Shida N., Senboku H., Tanaka K., Atobe M. (2021). Integrated Flow Synthesis
of α-Amino Acids by in Situ Generation
of Aldimines and Subsequent Electrochemical Carboxylation. J. Org. Chem..

[27] Qu Y., Tsuneishi C., Tateno H., Matsumura Y., Atobe M. (2017). Green Synthesis of
α-Amino Acids by Electrochemical Carboxylation
of Imines in a Flow Microreactor. React. Chem.
Eng..

[28] Li C. H., Yuan G. Q., Qi C. R., Jiang H. F. (2013). Copper-Catalyzed
Electrochemical Synthesis of Alkylidene Lactones from Carbon Dioxide
and 1,4-Diarylbuta-1,3-Diynes. Tetrahedron.

[29] Senboku H., Yamauchi Y., Kobayashi N., Fukui A., Hara S. (2011). Electrochemical
Carboxylation of Flavones: Facile Synthesis of Flavanone-2-Carboxylic
Acids. Electrochemistry.

[30] Franceschi P., Nicoletti C., Bonetto R., Bonchio M., Natali M., Dell’Amico L., Sartorel A. (2021). Basicity as a Thermodynamic Descriptor
of Carbanions Reactivity with Carbon Dioxide: Application to the Carboxylation
of α,β-Unsaturated Ketones. Front.
Chem..

[31] Harada J., Sakakibara Y., Kunai A., Sasaki K. (1984). Electrochemical
Carboxylation
of Alpha, Beta-Unsaturated Ketones with Carbon Dioxide. Bull. Chem. Soc. Jpn..

[32] Senboku H., Yamauchi Y., Kobayashi N., Fukui A., Hara S. (2012). Some Mechanistic
Studies on Electrochemical Carboxylation of Flavones to Yield Flavanone-2-Carboxylic
Acids. Electrochim. Acta.

[33] Sheta A., Alkayal A., Mashaly M., Said S., Elmorsy S., Malkov A. V., Buckley B. R. (2021). Selective
Electrosynthetic Hydrocarboxylation
of α,Β-Unsaturated Esters with Carbon Dioxide. Angew. Chemie - Int. Ed..

[34] Ang N. W. J., Oliveira J. C. A., Ackermann L. (2020). Electroreductive
Cobalt-Catalyzed Carboxylation: Cross-Electrophile Electrocoupling
with Atmospheric CO_2_. Angew. Chemie
- Int. Ed..

[35] Zhao R., Lin Z., Maksso I., Struwe J., Ackermann L. (2022). Electrochemical
Cross-Electrophile-Coupling for Transition- Metal-Free Allylic Carboxylation
with Ambient CO_2_. ChemElectroChem..

[36] Sheta A. M., Mashaly M. A., Said S. B., Elmorsy S. S., Malkov A. V., Buckley B. R. (2020). Selective α,δ-Hydrocarboxylation
of Conjugated
Dienes Utilizing CO_2_ and Electrosynthesis. Chem. Sci..

[37] Li C. H., Yuan G. Q., Ji X. C., Wang X. J., Ye J. S., Jiang H. F. (2011). Highly Regioselective
Electrochemical Synthesis of
Dioic Acids from Dienes and Carbon Dioxide. Electrochim. Acta.

[38] Matthessen R., Fransaer J., Binnemans K., Vos D. E. D. (2013). Electrochemical
Dicarboxylation of Conjugated Fatty Acids as an Efficient Valorization
of Carbon Dioxide. RSC Adv..

[39] Zhang W., Liao L. L., Li L., Liu Y., Dai L. F., Sun G. Q., Ran C. K., Ye J. H., Lan Y., Yu D. G. (2023). Electroreductive Dicarboxylation of Unactivated Skipped
Dienes with
CO_2_. Angew. Chemie - Int. Ed..

[40] Gao Z., Fletcher S. P. (2018). Construction of β to Carbonyl Stereogenic Centres
by Asymmetric 1,4-Addition of Alkylzirconocenes to Dienones and Ynenones. Chem. Commun..

[41] Yang X. Y., Tay W. S., Li Y., Pullarkat S. A., Leung P. H. (2015). Asymmetric 1,4-Conjugate Addition
of Diarylphosphines
to α,β,γ,δ-Unsaturated Ketones Catalyzed by
Transition-Metal Pincer Complexes. Organometallics.

[42] Amoah E., Dieter R. K. (2017). Regioselective 1,4-Conjugate Addition of Grignard Reagents
to α,β-γ,δ-Dienones and α,β-γ,δ-Dienyl
Thiol Esters. J. Org. Chem..

[43] Fuson R. C. (1935). The Principle
of Vinylogy. Chem. Rev..

[44] Christ R. E., Fuson R. C. (1937). The Application
of the Principle of Vinylogy to Unsaturated
Ketones. J. Am. Chem. Soc..

[45] Nicoletti C., Orlandi M., Dell’Amico L., Sartorel A. (2024). Unveiling the Reactivity
of CO_2_ with Carbanions: A Theoretical Analysis of the Carboxylation
Step. Sustain. Energy Fuels.

[46] Kochurin M. A., Ismagilova A. R., Zakusilo D. N., Khoroshilova O. V., Boyarskaya I. A., Vasilyev A. V. (2022). Reactions of Linear Conjugated Dienone
Structures with Arenes under Superelectrophilic Activation Conditions.
An Experimental and Theoretical Study of Intermediate Multicentered
Electrophilic Species. New J. Chem..

[47] Isse A. A., Gennaro A. (2002). Electrocatalytic Carboxylation
of Benzyl Chlorides
at Silver Cathodes in Acetonitrile. Chem. Comm.

[48] Chen R., Tian K., He D., Gao T., Yang G., Xu J., Chen H., Wang D., Zhang Y. (2020). Carboxylation of α,β-Unsaturated
Ketones by CO_2_ Fixation through Photoelectro-Chemistry. ACS Appl. Energy Mater..

[49] Gennaro A., Isse A. A., Vianello E. (1990). Solubility
and Electrochemical Determination
of CO_2_ in Some Dipolar Aprotic Solvents. J. Electroanal. Chem..

[50] Manabe S., Wong C. M., Sevov C. S. (2020). Direct
and Scalable Electroreduction
of Triphenylphosphine Oxide to Triphenylphosphine. J. Am. Chem. Soc..

[51] Villiers C., Dognon J. P., Pollet R., Thuéry P., Ephritikhine M. (2010). An Isolated CO_2_ Adduct
of a Nitrogen Base:
Crystal and Electronic Structures. Angew. Chemie
- Int. Ed..

[52] Box J. R., Avanthay M., Poole D. L., Lennox A. J. J. (2023). Electronically
Ambivalent Hydrodefluorination of Aryl-CF_3_ Groups Enabled
by Electrochemical Deep-Reduction on a Ni Cathode. Angew. Chem. Int. Ed..

[53] Isse A. A., Durante C., Gennaro A. (2011). One-Pot Synthesis of Benzoic Acid
by Electrocatalytic Reduction of Bromobenzene in the Presence of CO_2_. Electrochem. commun..

[54] Sisa M., Bonnet S. L., Ferreira D., Van Der Westhuizen J. H. (2010). Photochemistry
of Flavonoids. Molecules.

[55] Wang H., Zhu H. W., Guo R. R., Hu Q. L., Zeng S., Lu J. X. (2017). Computational and
Experimental Study on Electrocarboxylation of Benzalacetone. Asian J. Org. Chem..

[56] Zhang K., Ren B., Liu X., Wang L., Zhang M., Ren W.-M., Lu X.-B., Zhang W.-Z. (2022). Direct and Selective Electrocarboxylation
of Styrene Oxides with CO_2_ for Accessing β-Hydroxy
Acids. Angew. Chem. - Int. Ed..

[57] A carboxylation step of **1a** ^ *•–* ^ is endergonic at both δ and β carbon positions (Δ*G* ^0^ = +10.5 and +17.7 kcal mol^–1^, respectively), with activation free energies Δ*G* ^‡^ of 19.2 and 22.1 kcal mol^–1^, respectively.

[58] Isse A. A., Scialdone O., Galia A., Gennaro A. (2005). The Influence
of Aluminium
Cations on Electrocarboxylation Processes in Undivided Cells with
Al Sacrificial Anodes. J. Electroanal. Chem..

