# Supplementary material for: Vinylogous Electrochemical Carboxylation of Dienones
Source: ACS Electrochem. 2025 Jul 3;1(8):1443–51. doi: 10.1021/acselectrochem.5c00078 (PMC12337805; doi:10.1021/acselectrochem.5c00078)
Supplement: Supplementary file 1 [file ec5c00078_si_001.pdf]

Supporting Information

Vinylogous electrochemical carboxylation of dienones

Catia Nicoletti*, Elena Tacchi, Noemi Trovato, Manuel Orlandi, Luca Dell'Amico, Abdirisak Ahmed Isse, Marco Fantin*, Andrea Sartorel*

Department of Chemical Sciences, University of Padova, via Marzolo 1, 35131 Padova, Italy.

catia.nicoletti@phd.unipd.it; marco.fantin@unipd.it; andrea.sartorel@unipd.it

TABLE OF CONTENTS

A. GENERAL INFORMATION 5

A.1 CYCLIC VOLTAMMETRY 6

A.2 DFT CALCULATIONS 6

A.3 REDUCTION POTENTIAL CALCULATIONS 7

B. GENERAL INFORMATION FOR THE STARTING MATERIALS SYNTHESIS 8

B.1 PREPARATION OF EXTENDED UNSATURATED KETONES 8

B.1.1 PROCEDURE A FOR SYNTHESIS OF 1A-G AND 1J, 1N-Q 8

B.1.2 PROCEDURE B FOR SYNTHESIS OF 1H-I 9

B.1.3 PROCEDURE C FOR SYNTHESIS OF 1K 9

B.1.4 PROCEDURE D FOR SYNTHESIS OF 1K 9

C. ELECTROCHEMICAL CHARACTERIZATION 16

C.1 CV OF (1A) 16

C.2 CV OF (1B) 18

C.3 CV OF (1C) 18

C.4 CV OF (1D) 19

C.5 CV OF (1E) 19

C.6 CV OF (1F) 20

C.7 CV OF (1G) 20

C.8 CV OF (1H) 21

C.9 CV OF (1I) 21

C.10 CV OF (1J) 22

C.11 CV OF (1K) 22

C.12 CV OF (1L) 23

C.13 CV OF (1M) 23

C.14 CV OF (1N) 24
C.15 CV OF (1O) 24
C.14 CV OF (1P) 25
C.15 CV OF (1Q) 25

D. ELECTROCHEMICAL CARBOXYLATION OF CHALCONE 27

D.1 CV OF CHALCONE (S8) 27
D.2 ELECTROCHEMICAL CARBOXYLATION 27

E. REACTION OPTIMIZATION 29

E.1 2-ELECTRODE ELECTROCHEMICAL CELL SET UP 30

F. ELECTROCHEMICAL CARBOXYLATION PROCEDURES 31

G. CHARACTERIZATION DATA 33

H. ELECTROCHEMICAL DIMER SYNTHESIS 40

I. CV ANALYSIS AND DIGITAL SIMULATION 43

I.1 CV ANALYSIS 43
I.1 DIGITAL SIMULATION OF CV 44

J. DFT CALCULATIONS 46

J.1 REACTION PROFILE FOR $1A^{\bullet -}$ CARBOXYLATION 46
J.2 OPTIMIZED GEOMETRIES OF RADICAL ANIONS 47
J.3 OPTIMIZED GEOMETRIES OF DIANIONS 48

K. NMR SPECTRA 51

L. REFERENCES 78

A. General Information

NMR spectra were recorded on Bruker AVANCE Neo 400 Nanobay equipped with a BBFO-ATM-z grad probehead, Bruker 400 AVANCE III HD equipped with a BBI-z grad probe head 5mm, Bruker 500 AVANCE III equipped with a BBI-ATM-z grad probe head 5mm. The chemical shifts (δ) for ^{1}H and ^{13}C are given in ppm relative to residual signals of the solvents ($CDCl_3$: @7.26 ppm for ^{1}H NMR and @77.16 ppm for ^{13}C NMR, CD_3CN: @1.94 ppm for ^{1}H NMR and @118.26 ppm for ^{13}C NMR). Coupling constants are given in Hz. The following abbreviations are used to indicate the multiplicity: s, singlet; d, doublet; t, triplet; q, quartet; m, multiplet; br, broad signal. NMR yields were calculated by using CH_2Br_2 as internal standard. The standard Bruker pulse sequence *hmbcgplpndqf* was used for the ^{1}H-^{13}C HMBC experiments. The spectral sizes for the ^{1}H-^{13}C HMBC experiments were 5263.16 Hz (13.15 ppm) × 22149.75 Hz (220.00 ppm) with 128 increments in f1 and 4 transients per increment. A delay of 50 ms was optimised to select multiple-bond (^{n}JHC) couplings of 10 Hz. All HMBC spectra were presented in magnitude mode. The standard Bruker pulse sequence *cosygpppqf* was used for the ^{1}H-^{1}H COSY experiments. A spectral width of 4717 Hz (11.78 ppm) was used; the spectra were acquired with 128 increments, with a single scan for each increment. The standard Bruker pulse sequence *noesygpph* was used for the ^{1}H-^{1}H NOESY experiments. A spectral width of 4000 Hz (10 ppm) was used; the spectra were acquired in TPPI mode, with 400 increments and 2 scans for each increment and a mixing time of 1.5 seconds. The spectra were acquired with the help of Dr. Ileana Menegazzo (University of Padova). NMR spectra were processed using MestReNova software.

HRMS (ESI-MS) spectra (10 ppm resolution) were performed by Susanna Vogliardi at the department of pharmaceutical and pharmacological sciences (University of Padova) with a Xevo G2-S QTof (Waters) coupled with a UPLC system Acquity H Class (Waters).

The cyclic voltammetry (CV) characterizations were carried out on a BASi EC Epsilon potentiostat-galvanostat in a typical three-electrode cell.

Electrosynthesis experiments were performed with a Metrohm Autolab PGSTAT 2014 potentiostat-galvanostat in combination with the Nova 2.1.4 software (https://www.metrohm-autolab.com/Products/Echem/Software/Nova.html).

Chromatographic purification was accomplished using flash chromatography on silica gel (SiO_2, 0.04-0.063 mm) purchased from Machery-Nagel, with the indicated solvent system according to the standard techniques. Thin-layer chromatography (TLC) analysis was performed on pre-coated Merck TLC plates (silica gel 60 GF254, 0.25 mm). Visualization of the developed chromatography was performed by checking UV absorbance (254 nm) as well as with potassium permanganate and bromocresol green stains. Organic solutions were concentrated under reduced pressure on a Büchi rotary evaporator.

Preparative HPLC was developed on an Phennomenex Kinetex C_{18} column (100 Å, 150 mm × 21.2 mm, 5 μm) using a SPD–20A UV detector (Shimadzu Europa GmbH) and a Shimatzu LC-8A workstation. The concentration of crude solution for injection was 50 mg/mL.

All commercial grade solvents were purchased at the highest commercial quality from Sigma Aldrich and used as received, unless otherwise stated.

A.1 Cyclic voltammetry

Electrochemical characterization of **1a at** T= -15°C was performed on a Autolab PGSTAT 302N controlled with Nova 2.1 software with compensation of solution resistance. Measurement was made in a thermostated 5-necked glass cell under Ar or CO_2 saturated atmosphere using a three-electrodes setup, with a solution volume of 15 mL. Cyclic voltammetry experiments were conducted using as counter electrode (CE) a Pt wire, a glassy carbon (Tokai, 3mm diameter) working electrode (WE) and a saturated calomel electrode as reference electrode (RE). The WE was polished with 0.25 µm diamond paste and sonicated before use.

For the electrochemical characterization of all substrates (**1a-1q**) at ambient temperature, a typical three-electrode cell was employed, combining a glassy carbon (GC) working electrode (WE) (BioLogic, 3 mm nominal diameter, 7 mm^2 geometric area), a platinum electrode (BASi) as counter electrode (CE) and a silver/silver chloride electrode (Ag/AgCl/3M NaCl) as reference electrode (RE). Oxygen was removed by saturating the solution with high-purity Nitrogen (N_2) or carbon dioxide (CO_2). The glass electrochemical cell was kept closed during the measurements, the headspace of the cell being also degassed to prevent dioxygen contamination.

All potentials were then converted to ferrocenium/ferrocene (Fc^+/Fc), using an internal reference system,[1] upon addition, at the end of each experiment session, of ferrocene to the analyte solutions, and running a cyclic voltammogram from which the $E_{1/2}$ of the couple was measured.

The glassy carbon working electrode was polished before any measurement with a 1 μm diamond paste on a microfiber cloth (Struers), carefully rinsed with de-ionized water, then acetone and rinsed by applying ultrasonic for 1 minutes. After each series of CV experiments, the electrochemical cell was carefully rinsed with acetone, and de-ionized water; afterwards, the cell and the magnetic stirrer were sonicated for 5 min with acetone.

A.2 DFT calculations

All calculations were performed with Gaussian16 software at the C_3P facility of the University of Padova; Gauss View 6 software was used as a graphical interface to build the input and interpret the output.

After an initial conformational analysis, for all molecules, geometry optimisations and frequency calculations were done, using the density functional theory (DFT) method:

- b3lyp/6-311g(d,p)//b3lyp/6-311g(d,p): B3LYP functional and Pople 6-311g(d,p) basis set;
- ωb97xd/def2tzvp//ωb97xd/def2tzvp: ωB97XD long-range-corrected functional, which includes empirical dispersion and with def2tzvp Ahlrichs' triple-zeta basis set.

A correction of +1.90 $kcalmol^{-1}$ to the computed free energy and enthalpy values was applied to convert the standard state from 1 atm to a 1 M solution.[2,3]
The self-consistent reaction field (SCRF) was used with DFT energies, optimizations, and frequency calculations to model systems in acetonitrile solution. For all calculations a pruned (99,590) integration grid was used (keyword: Integral=UltraFine, the default choice in Gaussian16).
All the structures were optimised including a continuum solvation model for acetonitrile using the integral equation formalism variant (IEFPCM).
Stationary points on the potential energy surface were determined to be minima (no vibrational modes with imaginary frequency) and the actual nature of calculated transition states was confirmed by analysis of frequencies (only one mode with imaginary vibrational frequency) and the reliability of the transition states was corroborated by intrinsic reaction coordinate (IRC) computation.

The command expressed in all computed jobs in this work was:

- To obtain the optimized molecular structure and local minimum of energy: # opt freq b3lyp/6-311g(d,p) scrf=(solvent=acetonitrile) or # opt freq=noraman wb97xd scrf=(solvent=acetonitrile) def2tzvp
- To obtain the scan of the energy while breaking a C-COO bond: opt=modredundant b3lyp/6-311g(d,p) scrf=(solvent=acetonitrile) or # opt=modredundant wb97xd/def2tzvp scrf=(solvent=acetonitrile). Scans were done with an amplitude of 0.1 and 0.01 Å.
- To obtain the optimized molecular structure of a transition state and local maximum of energy: # opt=(calcfc,ts,noeigen) freq=noraman b3lyp/6-311g(d,p) scrf=(solvent=acetonitrile) or # opt=(calcfc,ts,noeigen) freq=noraman wb97xd/def2tzvp scrf=(solvent=acetonitrile)
- To obtain intrinsic reaction coordinate (IRC) profile: # irc=(calcfc,recorrect=never,maxpoints=20) wb97xd scrf=(solvent=acetonitrile) def2tzvp

A.3 Reduction Potential calculations

The potentials of the **1a**/**1a**$^{\bullet-}$ and **1a**$^{\bullet-}$/**1a**$^{2-}$ were calculated considering the combination of equation 3-5[4].

$$E^{0,SCE}_{red,ACN}\,(O/R) = E^{0,abs}_{red,ACN}\,(O/R) - E^{0,abs}_{ACN}\,(SCE) \quad \textit{(eq.3)}$$

$$\Delta_r G_{,ACN}\,(O/R) = -\,n_e \cdot F \cdot E^{0,abs}_{ACN}\,(O/R) \quad \textit{(eq.4)}$$

$$\Delta_r G\ (O/R) = G_R - G_O \qquad (eq.5)$$

$E^{0,SCE}_{red,ACN}$ (O/R) is referred to the reduction potential calculated (including a continuum model for acetonitrile solvent) for the couple **1a/1a**$^{\bullet-}$ or **1a**$^{\bullet-}$**/1a**$^{2-}$ (oxidised specie | reduced specie) with respect to SCE as reference electrode. $E^{0,abs}_{red,ACN}$ (O/R) is the absolute (i.e. no reference electrode is taken in consideration) calculated value of reduction potential of the couple **1a/1a**$^{\bullet-}$ or **1a**$^{\bullet-}$**/1a**$^{2-}$. $E^{0,abs}_{ACN}$ (SCE) is the absolute (i.e. no reference electrode is taken in consideration) calculated value of reduction potential of the reaction *(eq.6)*:

$$Hg_2Cl_2 + 2\ e^- \rightleftarrows 2Hg_{(l)} + 2Cl^- \qquad (eq.6)$$

$E^{0,abs}_{ACN}$ (O/R) can be calculated from the computed values of Gibbs free energies G_R and G_O, of the reduced and oxidised species, respectively. $\Delta_r G$ and F are expressed in kcal/mol (where F is the Faraday constant = 23.061 kcal/mol). Finally, – n_e is the number of electrons transferred in the reduction process, which is 1 in the case of **1a/1a**$^{\bullet-}$ or **1a**$^{\bullet-}$**/1a**$^{2-}$.

For a better comparation with the experimental values, the potentials were referred to Fc^+/Fc couple, subtracting the value of -0.403 V (as reported in ref[5]) to $E^{0,SCE}_{red,ACN}$ (**1a/1a**$^{\bullet-}$) and $E^{0,SCE}_{red,ACN}$ (**1a**$^{\bullet-}$**/1a**$^{2-}$). The reduction potentials resulted -1.7 V and -2.6 V vs Fc^+/Fc, respectively, in very good agreement with the experimental values of -1.7 and -2.5 V (from the cathodic peak potential E_{pc} of the two waves, see **Electrochemical characterization**).

B. General information for the starting materials synthesis

B.1 Preparation of extended unsaturated ketones

B.1.1 Procedure A for synthesis of **1a-g** and **1j**, **1n-q**

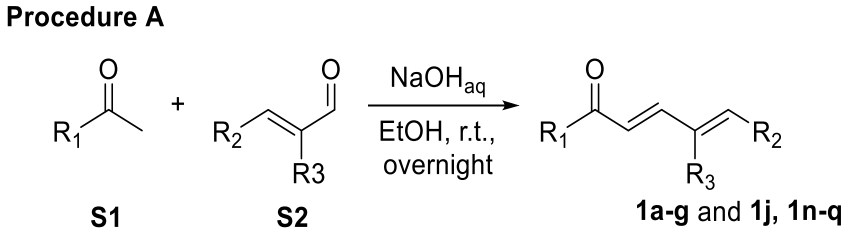

According to a modified literature procedure,[6] in a single neck round bottom flask with a stirrer magnet, ketone **S1** (5 mmol, 1 eq) was diluted in EtOH (27 mL) at 0°C. An aqueous solution of NaOH (0.14 M, 20 mL) added. After 30 min, aldehyde **S2** (5 mmol, 1 eq) was added dropwise and the solution was allowed to warm up to room temperature and further stirred overnight. At the end of the reaction a yellow precipitate was filtered on a Buckner funnel and washed with cold water. The precipitate was then diluted in CH_2Cl_2 (30 mL) and washed with water (15 mL) for 3 times in a separatory funnel. The dienones **1a-g** were isolated by flash chromatographic column on silica (eluent mixture: petroleum ether/ethyl acetate or petroleum ether/CH_2Cl_2).

B.1.2 Procedure B for synthesis of **1h-i**

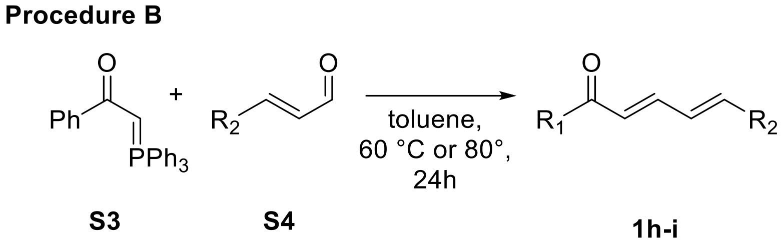

According to a modified literature procedure[6], in a single neck round bottom flask with a stirrer magnet, phosphonium ylide **S3** (5.5 mmol, 1.1 eq) and aldehyde **S4** (5 mmol, 1 eq) were diluted in dry toluene (22 mL). The reaction was conducted under N_2 atm and heated at 80°C (for compound **1h**) or 60°C (for compound **1i**) for 24h. At the end of the reaction, the crude was extracted with EtOAc (3 x 30 mL) and water (15 mL) and washed with brine. The dienones **1h-i** were isolated by flash chromatographic column on silica (eluent mixture: petroleum ether/ethyl acetate).

B.1.3 Procedure C for synthesis of **1k**

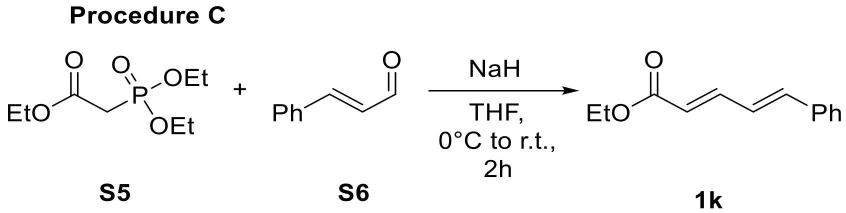

According to a modified literature procedure,[7] in a flamed-dry 2-neck round bottom flask with a stirrer magnet, NaH (60% in paraffin oil, 1.8 g, 1.5 eq) and dry THF were added at 0°C and under N_2 atm. Compound **S5** (36 mmol, 1.2 eq) was added dropwise and the solution was stirred for 30 min. Then (E)-3-phenylpropenal **S6** (30 mmol, 1 eq) was added dropwise to the solution and the reaction mixture was warmed to room temperature. After 2 h, the crude was neutralised with $NaHCO_3$ (50 mL), extracted with Et_2Oc (3 x 50 mL) and finally washed with brine. Compound **1k** was isolated as a yellow liquid by flash chromatographic column on silica (eluent mixture: petroleum ether/ethyl acetate).

B.1.4 Procedure D for synthesis of **1k**

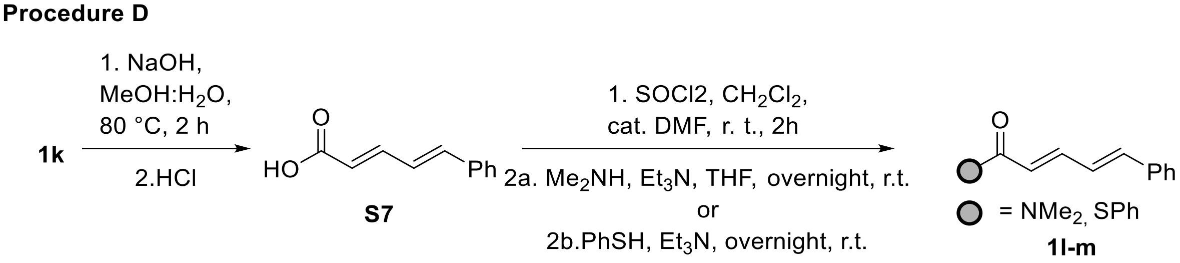

According to a modified literature procedure[8], in a single neck round bottom flask with a stirrer magnet, ester **1k** (6.5 mmol, 1 eq) and NaOH (12 mmol, 2 eq) were added to a solution

of MeOH:H_2O (8:1, 135 mL). The solution was heated at 80°C. The reaction mixture was checked by TLC and, after completion (ca 2 h), MeOH was evaporated with rotatory evaporator. HCl (2M, 15 mL) was added, and the solution was extracted with ETOAc (3 x 30 mL). The solvent was evaporated and carboxylic acid **S7** was obtained as white solid and used without further purification.

According to modified literature procedures[8,9], in a single neck round bottom flask with a stirrer magnet, **S7** (6 mmol, 1 eq) and dry CH_2Cl_2 (4.8 mL) were added under N_2 atm (*the N_2 balloon was equipped with a PTFE tube to avoid the corrosion of the needle*). $SOCl_2$ (7.2 mmol, 1.2 eq) was added dropwise. After the addition of a catalytic amount of DMF (60 μL), the solution was stirred for 2 h at room temperature.

<u>Step 2a:</u>

The crude reaction mixture was evaporated and redissolved in 2 mL of dry THF. A 2 M solution of Me_2NH in THF (12 mmol, 2 eq, 6 mL) was added dropwise. Et_3N (15 mmol, 2 eq) was added and the suspension was stirred overnight. The crude was washed with H_2O (10 mL) and extracted with EtOAc (3 x 20 mL). Compound **1l** was isolated by flash chromatographic column on silica (eluent mixture: petroleum ether/ethyl acetate).

<u>Step 2b:</u>

To the crude reaction mixture PhSH (6 mmol, 1 eq) was added dropwise. Et_3N (15 mmol, 2 eq) was also added and the suspension was stirred overnight. The crude was washed with H_2O (10 mL) and extracted with EtOAc (3 x 20 mL). Compound **1m** was isolated by flash chromatographic column on silica (eluent mixture: petroleum ether/ethyl acetate).

<u>Characterization data</u>

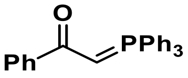

S3 1-phenyl-2-(triphenylphosphanylidene)ethan-1-one (**S3**) was synthesized according to a literature procedure[10] from 2-bromo-1-phenylethan-1-one and triphenylphosphine as a white solid (4.39 g, 76% yield). **^{1}H-NMR** (400 MHz, $CDCl_3$): δ 7.97 (dd, *J* = 6.5, 2.9 Hz, 2H), 7.72 (ddd, *J* = 12.6, 8.4, 1.4 Hz, 6H), 7.56 (ddd, *J* = 7.2, 5.2, 1.6 Hz, 3H), 7.52 – 7.41 (m, 6H), 7.40 – 7.31 (m, 3H), 4.43 (d, *J* = 24.5 Hz, 1H) ppm. The characterization data matched with the reported one.[10]

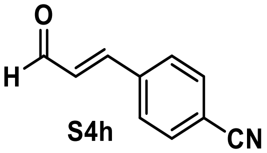

S4h 4-(3-oxoprop-1-en-1-yl)benzonitrile (**S4h**) was synthesized according to a literature procedure[11] from 2-(triphenylphosphanylidene)acetaldehyde and 4-formylbenzonitrile as a yellow solid (0.88 g, 74% yield, E:Z = 10:1.5). (E)-isomer : **^{1}H-NMR** (400 MHz, $CDCl_3$): (E)-isomer: δ 9.76 (d, *J* = 7.5 Hz, 1H), 7.73 (d, *J* = 8.4 Hz, 2H), 7.66 (d, *J* = 8.3 Hz, 2H), 7.48 (d, *J* = 16.1 Hz, 1H), 6.77 (dd, *J* = 16.1, 7.5 Hz, 1H) ppm. **^{13}C-NMR**

(101 MHz, $CDCl_3$): δ 193.0, 149.5, 138.2, 132.9 (x2), 131.3, 128.8 (x2), 118.2, 114.4 ppm. The characterization data matched with the reported one.[11]

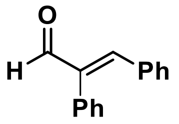

S2j 2,3-diphenylacrylaldehyde (**S2j**) was synthesized according to a literature procedure[12] from 2-phenylacetaldehyde and benzaldehyde as a white solid (0.88 g, 74% yield, E:Z = 10:0.3). **^{1}H-NMR** (400 MHz, $CDCl_3$): δ 9.78 (s, 1H), 7.49 – 7.34 (m, 4H), 7.32 – 7.27 (m, 1H), 7.24 – 7.17 (m, 6H) ppm. **^{13}C-NMR** (101 MHz, $CDCl_3$): δ 194.07, 150.29, 141.96, 134.17, 133.48, 130.8 (x2), 130.3, 129.4 (x2), 129.0 (x2), 128.6 (x2), 128.4 ppm. The characterization data matched with the reported one.[12]

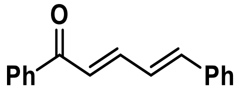

1a 1,5-diphenylpenta-2,4-dien-1-one (**1a**) was synthesized according to the general **Procedure A** from acetophenone and *trans*-cinnamaldehyde. **1a** was obtained as a yellow solid (1.47 g, 70% yield). **^{1}H-NMR** (400 MHz, $CDCl_3$): δ 8.02 – 7.95 (m, 2H), 7.65 – 7.54 (m, 2H), 7.53 – 7.46 (m, 4H), 7.42 – 7.31 (m, 3H), 7.10 (d, J = 14.9 Hz, 1H), 7.05 – 7.01 (m, 2H) ppm. **^{13}C-NMR** (101 MHz, $CDCl_3$): δ 190.6, 145.0, 142.0, 138.3, 136.2, 132.8, 129.3, 129.0 (x2), 128.7 (x2), 128.5 (x2), 127.4 (x2), 127.1, 125.6 ppm. The characterization data matched with the reported one.[6]

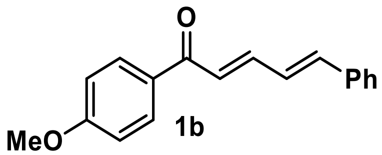

1b 1-(4-methoxyphenyl)-5-phenylpenta-2,4-dien-1-one (**1b**) was synthesized according to the general **Procedure A** from 1-(4-methoxyphenyl)ethanone (5 mmol, 1 eq) and *trans*-cinnamaldehyde (5 mmol, 1 eq). **1b** was obtained as a yellow solid (0.85 g, 64 % yield). **^{1}H-NMR** (400 MHz, $CDCl_3$): δ 8.03 – 7.94 (m, 2H), 7.60 (ddd, J = 14.9, 7.8, 2.4 Hz, 1H), 7.53 – 7.48 (m, 2H), 7.36 (dt, J = 15.4, 7.1 Hz, 3H), 7.11 (d, J = 14.9 Hz, 1H), 7.06 – 6.95 (m, 4H), 3.89 (s, 3H) ppm. **^{13}C-NMR** (101 MHz, $CDCl_3$): δ 188.8, 163.4, 144.1, 141.5, 136.3, 131.3 (x2), 130.8, 129.2, 128.9(x2), 127.3 (x2), 127.2, 125.4, 113.9 (x2), 55.6 ppm. The characterization data matched with the reported one.[13]

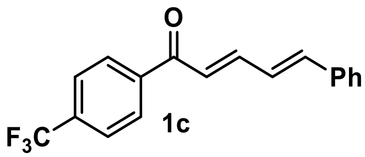

1c 5-phenyl-1-(4-(trifluoromethyl)phenyl)penta-2,4-dien-1-one (**1c**) was synthesized according to the general **Procedure A** from 1-(4-(trifluoromethyl)phenyl)ethan-1-one and *trans*-cinnamaldehyde (5 mmol, 1 eq). **1c** was

obtained as a yellow solid (1.03 g, 68 % yield). **^{1}H-NMR** (400 MHz, $CDCl_3$): δ 8.06 (d, *J* = 8.1 Hz, 2H), 7.76 (d, *J* = 8.1 Hz, 2H), 7.63 (ddd, *J* = 15.0, 7.9, 2.4 Hz, 1H), 7.55 – 7.48 (m, 2H), 7.45 – 7.30 (m, 3H), 7.12 – 6.99 (m, 3H) ppm. **^{19}F-NMR** (377 MHz, $CDCl_3$) δ -63.00 ppm.**^{13}C-NMR** (101 MHz, $CDCl_3$): 189.7, 146.2, 143.1, 141.2, 136.0, 134.0 (q, *J* = 32.6 Hz), 129.6, 129.0 (x2), 128.7 (x2), 127.5 (x2), 126.7, 125.7 (d, *J* = 3.5 Hz), 124.9 ppm. The characterization data matched with the reported one.[14]

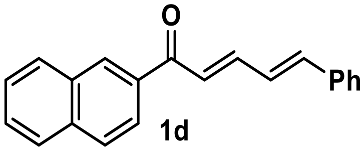

1d

1-(naphthalen-2-yl)-5-phenylpenta-2,4-dien-1-one (**1d**) was synthesized according to the general **Procedure A** from 1-(naphthalen-2-yl)ethan-1-one (5 mmol, 1 eq) and *trans*-cinnamaldehyde (5 mmol, 1 eq). **1d** was obtained as a yellow solid (0.85 g, 60 % yield). **^{1}H-NMR** (400 MHz, $CDCl_3$): δ 8.50 (s, 1H), 8.08 (dd, *J* = 8.6, 1.8 Hz, 1H), 7.99 (d, *J* = 7.9 Hz, 1H), 7.92 (dd, *J* = 14.7, 8.3 Hz, 2H), 7.73 – 7.47 (m, 5H), 7.43 – 7.28 (m, 4H), 7.14 – 7.00 (m, 2H) ppm. **^{13}C-NMR** (101 MHz, $CDCl_3$): δ 190.4, 144.9, 142.0, 136.3, 135.7, 135.5, 132.7, 129.9, 129.6, 129.3, 129.0 (x2), 128.6, 128.4, 127.9, 127.4, 127.1 (x2), 126.8, 125.6, 124.6 ppm. The characterization data matched with the reported one.[15]

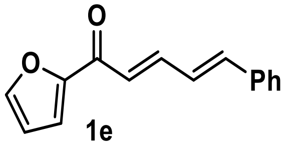

1e

1-(furan-2-yl)-5-phenylpenta-2,4-dien-1-one (**1e**) was synthesized according to the general **Procedure A** from 1-(furan-2-yl) ethan-1-one (5 mmol, 1 eq) and *trans*-cinnamaldehyde (5 mmol, 1 eq). **1e** was obtained as a yellow solid (0.70 g, 63 % yield). **^{1}H-NMR** (400 MHz, $CDCl_3$): δ 7.71 – 7.61 (m, 2H), 7.55 – 7.48 (m, 2H), 7.41 – 7.30 (m, 3H), 7.29 – 7.26 (m, 1H), 7.05 – 6.97 (m, 3H), 6.58 (dd, *J* = 3.6, 1.7 Hz, 1H) ppm. **^{13}C-NMR** (101 MHz, $CDCl_3$): δ 178.2, 153.9, 146.5, 144.1, 142.2, 136.2, 129.4, 128.9 (x2), 127.4 (x2), 126.9, 124.8, 117.3, 112.5 ppm. The characterization data matched with the reported one.[16]

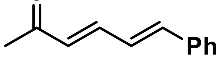

1f

6-phenyl-3,5-hexadien-2-one (**1f**) was synthesized according to the general **Procedure A** from acetone (used as a solvent, 135 mmol, 13.5 eq, 10 ml) and *trans*-cinnamaldehyde (10 mmol, 1 eq). **1f** was obtained as a pale yellow solid (0.75 g, 32 % yield). **^{1}H-NMR** (400 MHz, $CDCl_3$): δ 7.52 – 7.49 (m, 2H), 7.42 –7.31 (m, 4H), 7.03 – 6.86 (m, 2H), 6.29 (d, J = 15.5 Hz), 2.35 (s, 3H) ppm. **^{13}C-NMR** (101 MHz, $CDCl_3$): δ 198.4, 143.46, 141.2, 135.9, 130.5, 129.2, 128.8 (x2), 127.2 (x2), 126.6, 27.4 ppm. The characterization data matched with the reported one.[17]

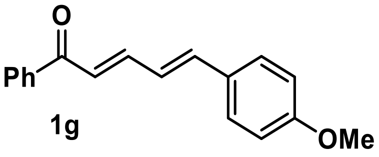

1g

5-(4-methoxyphenyl)-1-phenylpenta-2,4-dien-1-one (**1g**) was synthesized according to the general **Procedure A** from acetophenone (5 mmol, 1 eq) and and (E)-3-(4-methoxyphenyl)acrylaldehyde (5 mmol, 1 eq). **1g** was obtained as a yellow solid (0.81 g, 62 % yield). **^{1}H-NMR** (400 MHz, $CDCl_3$): δ 7.97 (d, *J* = 7.4 Hz, 2H), 7.67 – 7.53 (m, 2H), 7.47 (dd, *J* = 16.3, 8.2 Hz, 4H), 7.05 (d, *J* = 14.9 Hz, 1H), 7.01 – 6.86 (m, 4H), 3.84 (s, 3H) ppm. **^{13}C-NMR** (101 MHz, $CDCl_3$): 190.7, 160.7, 145.5, 141.9, 138.5, 132.6, 129.11, 128.9 (x2), 128.6 (x2), 128.4 (x2), 125.0, 124.4, 114.4 (x2), 55.5 ppm. The characterization data matched with the reported one.[18]

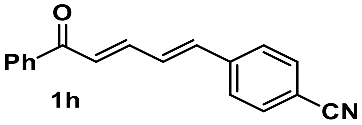

1h

4-(5-oxo-5-phenylpenta-1,3-dien-1-yl)benzonitrile (**1h**) was synthesized according to the general **Procedure B** from previously synthetised compound **S4a** (2 mmol, 1 eq) and phosphonium ylide **S3** (2.2 mmol, 1.1 eq). **1h** was obtained as a yellow solid (265 mg , 50 % yield). **^{1}H-NMR** (400 MHz, $CDCl_3$): δ 8.04 – 7.87 (m, 2H), 7.66 (d, *J* = 8.2 Hz, 2H), 7.62 – 7.54 (m, 4H), 7.54 – 7.47 (m, 2H), 7.21 – 7.06 (m, 2H), 6.99 (d, *J* = 15.6 Hz, 1H) ppm. **^{13}C-NMR** (101 MHz, $CDCl_3$): δ 190.29, 143.45, 140.56, 139.19, 138.02, 133.13, 132.75, 130.45, 128.84, 128.58, 127.69, 127.61, 118.79, 112.25 ppm. **HRMS (ESI-MS)** calculated for $C_{18}H_{14}NO^+$ $[M+H]^+$ 260.1071, found 260.1131.

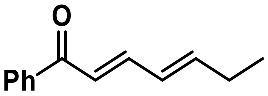

1i

1-phenylhepta-2,4-dien-1-one (**1i**) was synthesized according to the general **Procedure B** from (E)-pent-2-enal **S4i** (5 mmol, 1 eq) phosphonium ylide **S3** (5.5 mmol, 1 eq) (5 mmol, 1 eq). **1i** was obtained as a yellow oil (422 mg, 45 % yield). **^{1}H-NMR** (400 MHz, $CDCl_3$): δ 8.00 – 7.89 (m, 2H), 7.61 – 7.51 (m, 1H), 7.52 – 7.32 (m, 3H), 6.89 (d, *J* = 15.0 Hz, 1H), 6.31 (dd, *J* = 6.2, 2.9 Hz, 2H), 2.29 – 2.21 (m, 2H), 1.08 (t, *J* = 7.5 Hz, 3H) ppm.**^{13}C-NMR** (101 MHz, $CDCl_3$): δ 191.1, 148.0, 145.6, 138.4, 132.6, 128.6 (x2), 128.5 (x2), 128.3, 123.7, 26.4, 13.0 ppm. The characterization data matched with the reported one.[19]

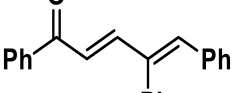

1j

1,4,5-triphenylpenta-2,4-dien-1-one (**1j**) was synthesized according to the general **Procedure A** from acetophenone (5 mmol, 1 eq) and previously synthetised (E)-2,3-diphenylpropenal (5 mmol, 1 eq). **1j** was obtained as a yellow solid (0.56 g, 36 % yield). **^{1}H-NMR** (400 MHz, $CDCl_3$): δ 7.88 – 7.77 (m, 3H), 7.46 (ddd, J = 24.8, 15.1, 7.4 Hz, 7H), 7.25 – 7.21 (m, 1H*), 7.18 – 7.10 (m, 3H), 7.04 (s, 1H), 7.00 – 6.95 (m, 2H), 6.58 (d, J = 15.1 Hz,

1H) ppm. **^{13}C-NMR** (101 MHz, $CDCl_3$): δ 190.89, 150.03, 140.34, 140.31, 138.45, 137.29, 135.91, 132.71, 130.30 (x2), 129.41 (x4), 128.63 (x2), 128.54, 128.51(x2), 128.36 (x2), 128.12, 124.37 ppm. **HRMS (ESI-MS)** calculated for $C_{23}H_{19}O^+$ $[M+H]^+$ 311.1440, found 311.1444. *Signal over-imposed with $CDCl_3$ signal.

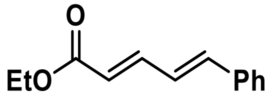

1k

Ethyl 5-phenylpenta-2,4-dienoate (**1k**) was synthesized according to the **Procedure C** as a yellow oil (3.35 g, 55 % yield). **^{1}H-NMR** (400 MHz, $CDCl_3$): δ 7.50 – 7.41 (m, 3H), 7.39 – 7.28 (m, 3H), 6.88 (d, *J* = 8.6 Hz, 2H), 5.99 (d, *J* = 15.3 Hz, 1H), 4.23 (q, *J* = 7.2 Hz, 2H), 1.32 (t, *J* = 7.1 Hz, 3H) ppm. **^{13}C-NMR** (101 MHz, $CDCl_3$): δ 167.2, 144.6, 140.4, 136.1, 129.1, 128.9 (x2), 127.3 (x2), 126.4, 121.4, 60.4, 14.4 ppm. The characterization data matched with the reported one.[7]

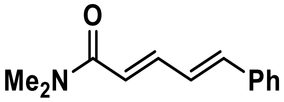

1l

N,N-dimethyl-5-phenylpenta-2,4-dienamide (**1l**) was synthesized according to the general **Procedure D**. **1l** was obtained as a white solid (0.80 g, 66 % yield). **^{1}H-NMR** (400 MHz, $CDCl_3$): δ 7.50 – 7.40 (m, 3H), 7.34 (t, *J* = 7.4 Hz, 2H), 7.31 – 7.27 (m, 1H), 6.98 – 6.81 (m, 2H), 6.46 (d, *J* = 14.7 Hz, 1H), 3.12 (s, 3H), 3.05 (s, 3H) ppm. **^{13}C-NMR** (101 MHz, $CDCl_3$): δ 166.7, 142.4, 138.9, 136.4, 128.7 (x2), 128.6, 126.98 (x2), 126.91, 120.6, 37.3, 35.8 ppm. The characterization data matched with the reported one.[8]

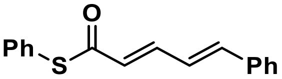

1m

S-phenyl (2E,4E)-5-phenylpenta-2,4-dienethioate (**1m**) was synthesized according to the general **Procedure D**. **1m** was obtained as a yellow solid (0.28 g, 16 % yield). **^{1}H-NMR** (400 MHz, $CDCl_3$): δ 7.52 – 7.41 (m, 8H), 7.41 – 7.30 (m, 3H), 7.01 (d, *J* = 15.6 Hz, 1H), 6.87 (dd, *J* = 15.5, 10.9 Hz, 1H), 6.35 (d, *J* = 15.0 Hz, 1H) ppm. **^{13}C-NMR** (101 MHz, $CDCl_3$: δ 187.9, 142.5, 141.7, 136.0, 134.7 (x2), 129.54, 129.52, 129.3 (x2), 129.0 (x2), 127.8, 127.4 (x2), 127.2, 126.1 ppm. **HRMS (ESI-MS)** calculated for $C_{17}H_{15}OS^+$ $[M+H]^+$ 267.0838, found 267.0866.

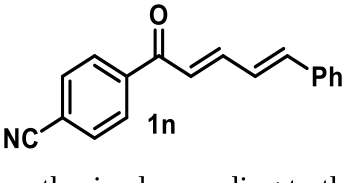

1n

4-((2E,4E)-5-phenylpenta-2,4-dienoyl)benzonitrile (**1n**) was synthesized according to the general **Procedure A** from 4-acetylbenzonitrile (5 mmol, 1 eq) and *trans*-cinnamaldehyde (6 mmol, 1.2 eq). **1n** was obtained as a yellow solid (0.940 g, 35 % yield). **^{1}H-NMR** (400 MHz, $CDCl_3$): δ 8.04 (d, *J* = 8.1 Hz, 2H), 7.79 (d, *J* = 8.1 Hz, 2H),

7.63 (dd, J = 14.9, 9.5 Hz, 1H), 7.52 (d, J = 7.2 Hz, 2H), 7.37 (dd, J = 10.1, 7.1 Hz, 3H), 7.14 – 6.97 (m, 3H). **^{13}C-NMR** (101 MHz, $CDCl_3$: δ = 189.1, 146.6, 143.5, 141.6, 135.9, 132.6 (x2), 129.7, 129.0(x2), 128.8 (x2), 127.6 (x2), 126.6, 124.5, 118.21 (CN), 115.8 ppm.The characterization data matched with the reported one.[20]

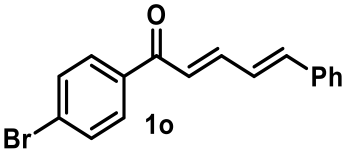

1o

(2E,4E)-1-(4-bromophenyl)-5-phenylpenta-2,4-dien-1-one (**1o**) was synthesized according to the general **Procedure A** from 1-(4-bromophenyl)ethan-1-one (15 mmol, 1 eq) and *trans*-cinnamaldehyde (18 mmol, 1.2 eq). **1n** was obtained as a yellow solid (0.940 g, 35 % yield). **^{1}H-NMR** (400 MHz, $CDCl_3$): δ 7.92 – 7.78 (m, 2H), 7.69 – 7.53 (m, 3H), 7.53 – 7.47 (m, 2H), 7.44 – 7.30 (m, 3H), 7.09 – 6.94 (m, 3H) ppm. **^{13}C-NMR** (101 MHz, $CDCl_3$: δ = 124.8, 126.8, 127.4 (x2), 127.8, 128.9(x2), 129.4, 129.9 (x2), 131.9 (x2),136.0, 136.9, 142.5, 145.4, 189.4 ppm. The characterization data matched with the reported one.[21]

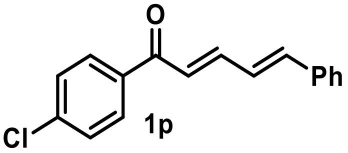

1p

(2E,4E)-1-(4-chlorophenyl)-5-phenylpenta-2,4-dien-1-one (**1p**) was synthesized according to the general **Procedure A** from 1-(4-chlorophenyl)ethan-1-one (10 mmol, 1 eq) and *trans*-cinnamaldehyde (12 mmol, 1.2 eq). **1p** was obtained as a yellow solid (1.61 g, 60 % yield). **^{1}H-NMR** (400 MHz, $CDCl_3$): δ 7.94 – 7.88 (m, 2H), 7.61 (ddd, J = 14.9, 7.0, 3.2 Hz, 1H), 7.55 – 7.43 (m, 4H), 7.43 – 7.30 (m, 3H), 7.13 – 6.98 (m, 3H). **^{13}C-NMR** (101 MHz, $CDCl_3$): δ 189.2, 145.4, 142.5, 139.2, 136.6, 136.1, 129.9 (x2), 129.52, 129.04 (x2), 129.05(x2), 127.5 (x2), 126.9, 124.9. The characterization data matched with the reported one.[21]

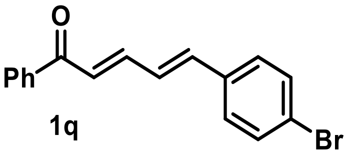

1q

(2E,4E)-5-(4-bromophenyl)-1-phenylpenta-2,4-dien-1-one (**1q**) was synthesized according to the general **Procedure A** from acetophenone (8.5 mmol, 1 eq) and (E)-3-(4-bromophenyl)acrylaldehyde (8.5 mmol, 1 eq). **1q** was obtained as a yellow solid (1.09 g, 23 % yield). **^{1}H-NMR** (400 MHz, $CDCl_3$): 8.02 – 7.93 (m, 2H), 7.63 – 7.53 (m, 2H), 7.53 – 7.45 (m, 4H),7.36 (d, J=8.4Hz, 2H), 7.11 (d, J = 15 Hz, 1H), 7.07 – 6.88 (m, 2H) ppm. **^{13}C-NMR** (75MHz, $CDCl_3$): 190.5, 144.5, 140.5, 138.2, 135.1, 132.9, 132.2 (x2), 128.8 (x2), 128.7 (x2), 128.5 (x2), 127.7, 126.0, 123.3 ppm. **HRMS (ESI-MS)** calculated for $C_{17}H_{13}BrO^+$ $[M+H]^+$ 313.0223, found 313.0275.

C. ELECTROCHEMICAL CHARACTERIZATION

Electrochemical characterization under Ar or CO_2 saturated atmosphere at T= -15 °C was performed for model compound **1a**, **Figure S1** shows the CVs under Ar atmosphere at different scan rates.

C.1 CV OF (1A)

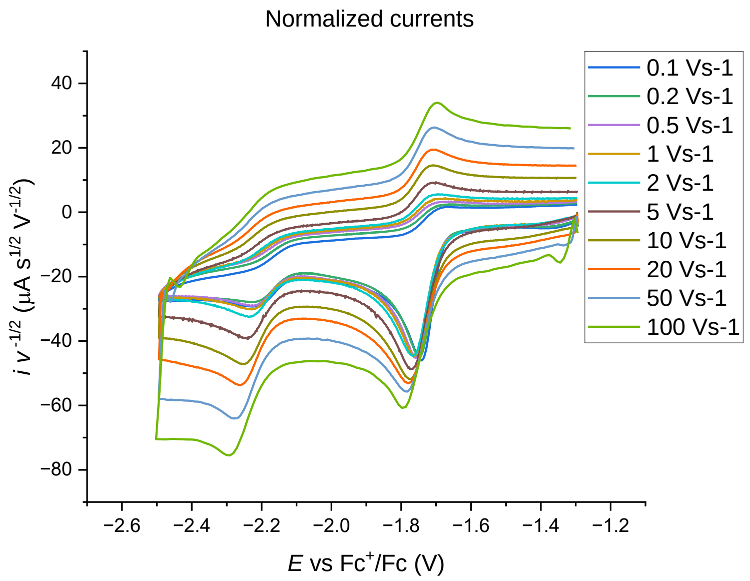

a)

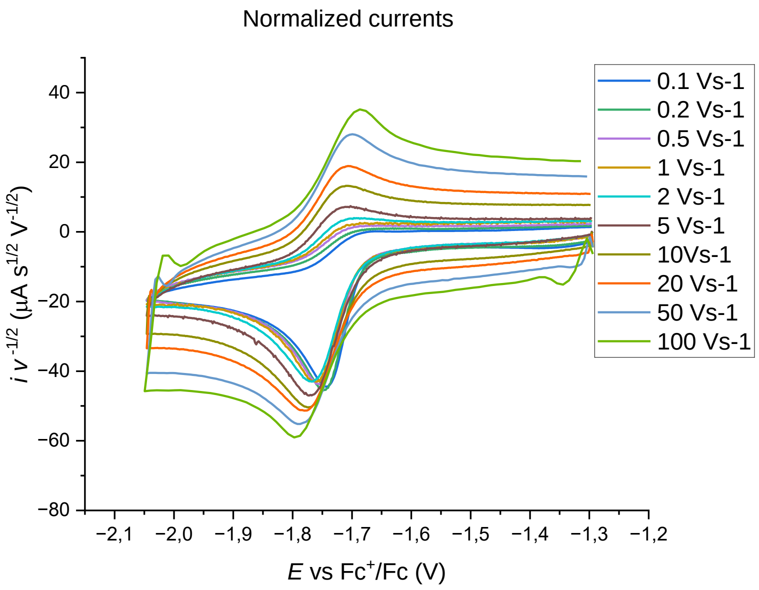

b)

Figure S1. CVs of **1a** (0.525 mM) in CH_3CN + 0.1 M n-Bu_4NPF_6 recorded at different scan rates on a GC disk (3 mm diameter) at -15 °C, under Ar atmosphere. a) CVs of **1a,** including both reduction waves. b) CVs of **1a,** including only the first reduction event.

The electrochemical properties of the starting compounds **1a-q** were also all characterized by CV experiments, conducted in acetonitrile (CH_3CN), with 0.1 M tetrabutylammonium hexafluorophosphate ($TBAPF_6$) at room temperature, and with a scan rate of 0.1 $V{\cdot}s^{-1}$.

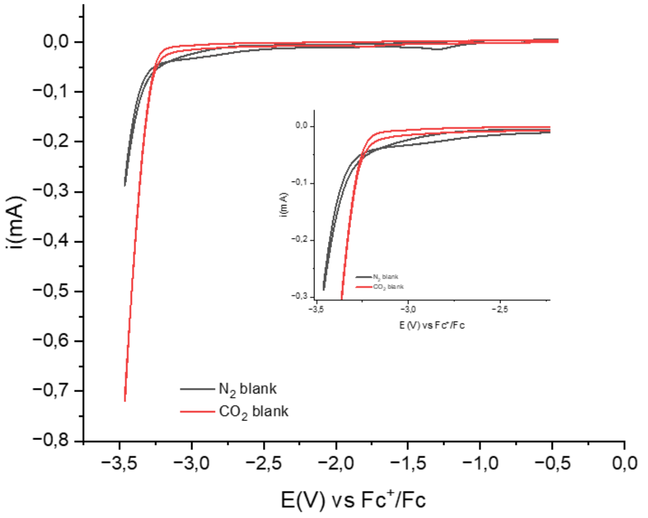

a)

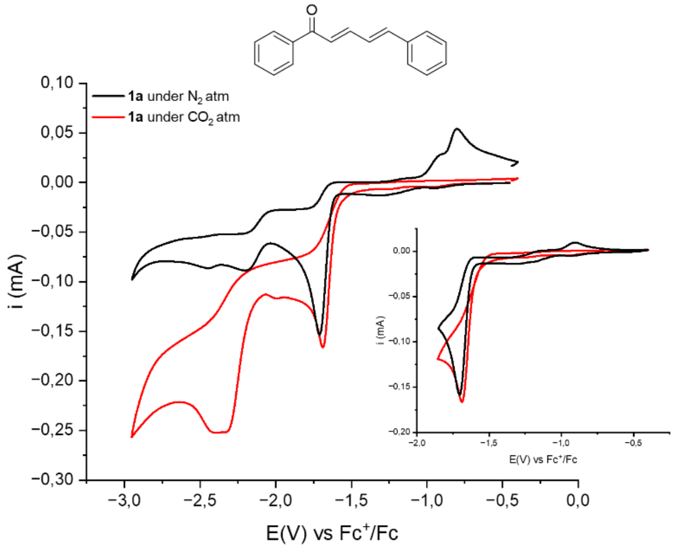

b)

Figure S2. Cyclic voltammetry of a) blank solution of only $TBAPF_6$ electrolyte (0.1 M in CH_3CN) and b) **1a** (1 mM in CH_3CN) under N_2 (black trace) and CO_2 (red trace) atmosphere. The inset in a) highlight that no cathodic current is associated to CO_2 reduction at potential of -2.5 V vs Fc^+/Fc. The inset in b) highlight only the first reduction event of **1a**.

C.2 CV OF (1B)

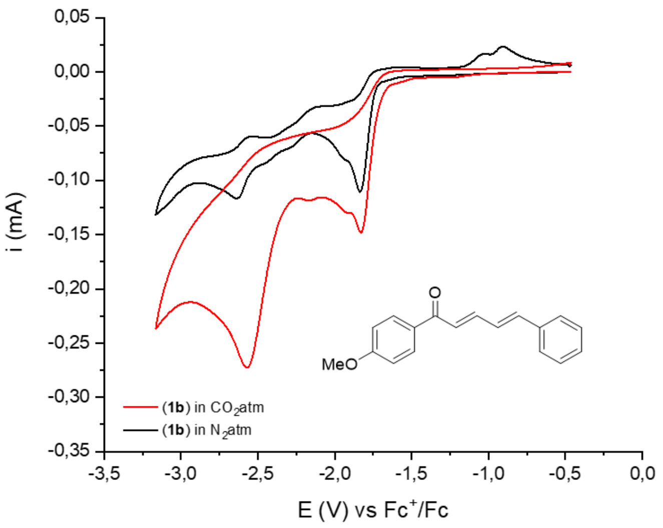

Figure S3. Cyclic voltammetry of **1b** (1 mM in CH_3CN) under N_2 (black trace) and CO_2 (red trace) atmosphere.

C.3 CV OF (1C)

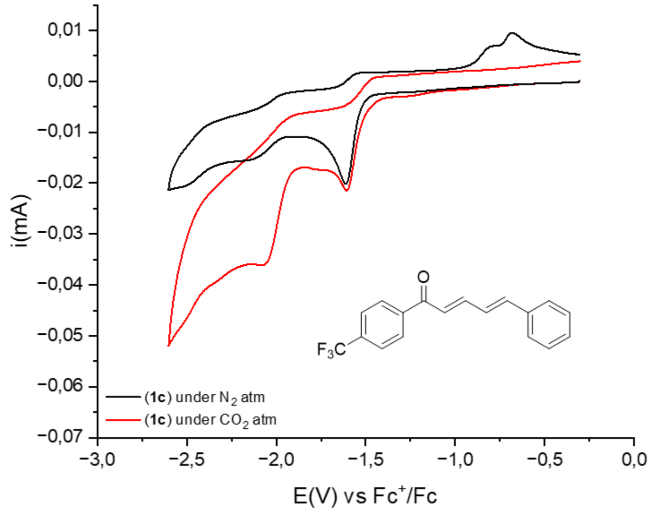

Figure S4. Cyclic voltammetry of **1c** (1 mM in CH_3CN) under N_2 (black trace) and CO_2 (red trace) atmosphere.

C.4 CV OF (1D)

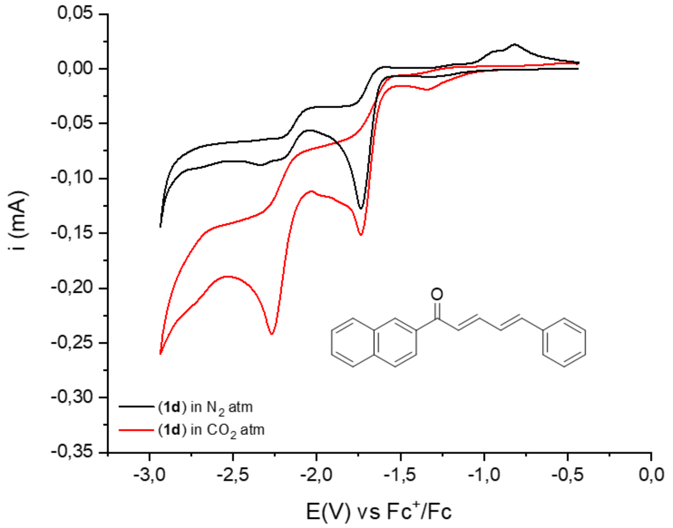

Figure S5. Cyclic voltammetry of **1d** (1 mM in CH_3CN) under N_2 (black trace) and CO_2 (red trace) atmosphere.

C.5 CV OF (1E)

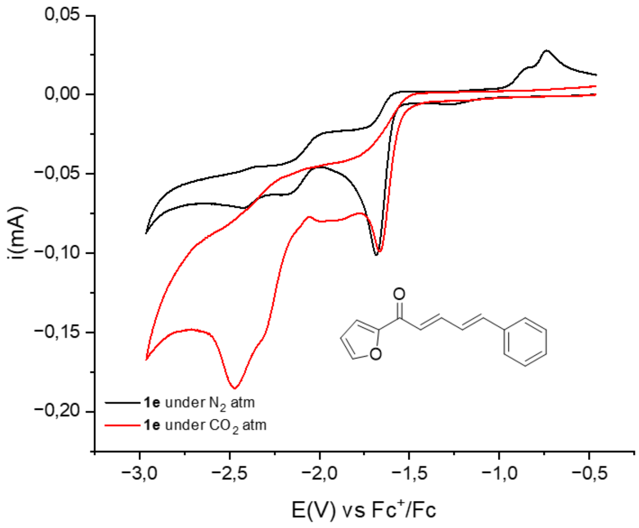

Figure S6. Cyclic voltammetry of **1d** (1 mM in CH_3CN) under N_2 (black trace) and CO_2 (red trace) atmosphere.

C.6 CV OF (1F)

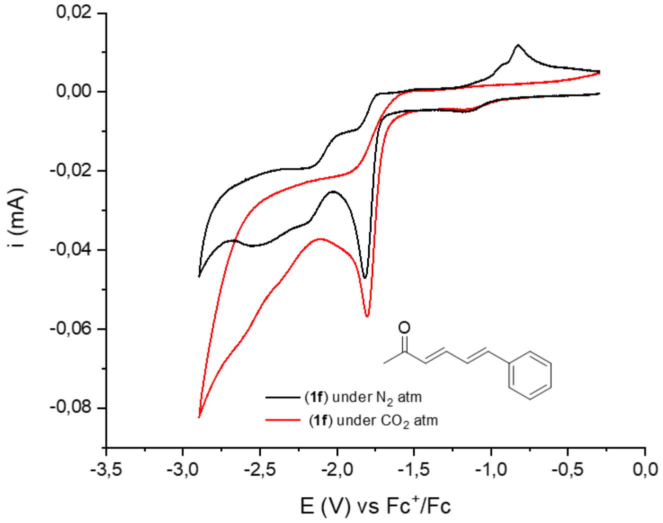

Figure S7. Cyclic voltammetry of **1f** (1 mM in CH_3CN) under N_2 (black trace) and CO_2 (red trace) atmosphere.

C.7 CV OF (1G)

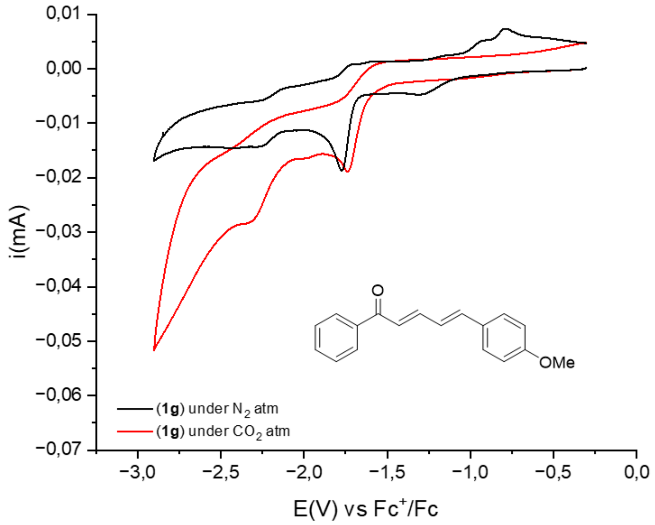

Figure S8. Cyclic voltammetry of **1g** (1 mM in CH_3CN) under N_2 (black trace) and CO_2 (red trace) atmosphere.

C.8 CV OF (1H)

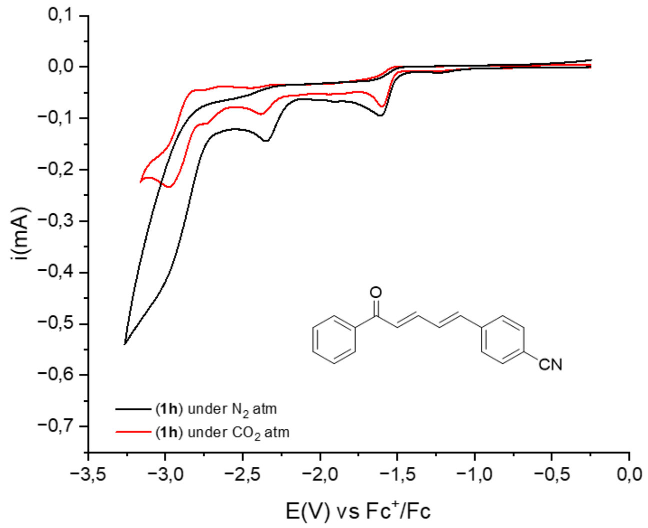

Figure S9. Cyclic voltammetry of **1h** (1 mM in CH_3CN) under N_2 (black trace) and CO_2 (red trace) atmosphere.

C.9 CV OF (1I)

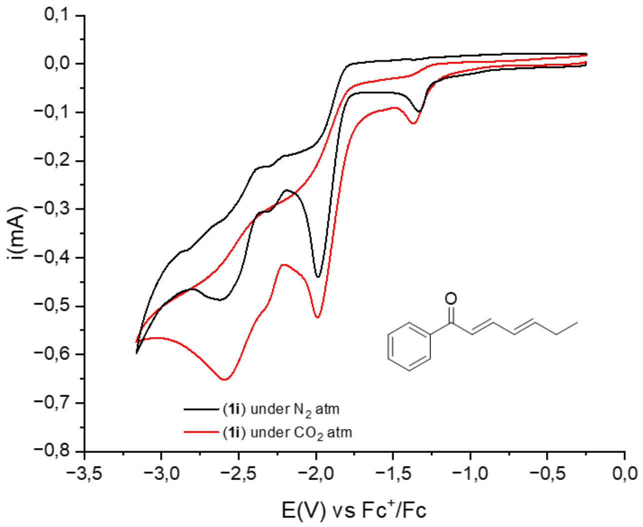

Figure S10. Cyclic voltammetry of **1i** (1 mM in CH_3CN) under N_2 (black trace) and CO_2 (red trace) atmosphere.

C.10 CV of (1j)

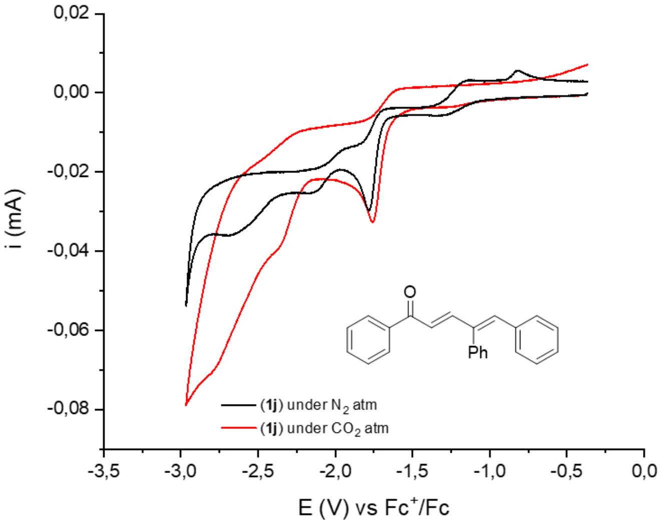

Figure S11. Cyclic voltammetry of **1j** (1 mM in CH_3CN) under N_2 (black trace) and CO_2 (red trace) atmosphere.

C.11 CV of (1k)

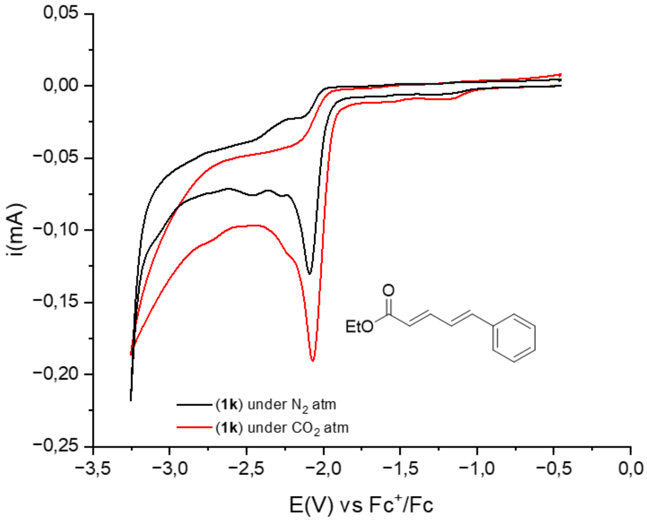

Figure S12. Cyclic voltammetry of **1k** (1 mM in CH_3CN) under N_2 (black trace) and CO_2 (red trace) atmosphere.

C.12 CV OF (1L)

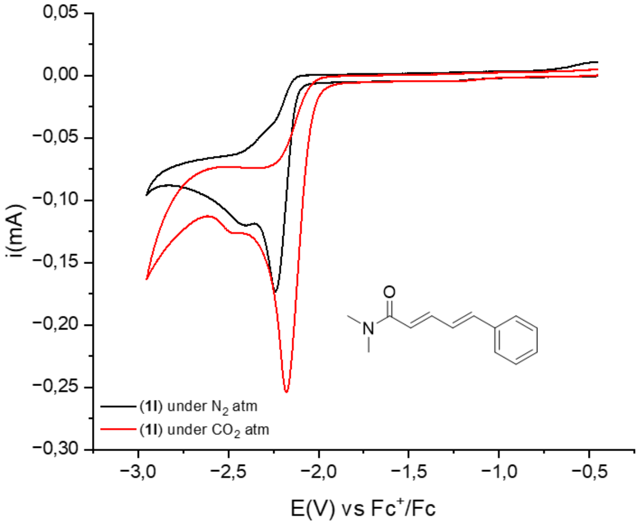

Figure S13. Cyclic voltammetry of **1l** (1 mM in CH_3CN) under N_2 (black trace) and CO_2 (red trace) atmosphere.

C.13 CV OF (1M)

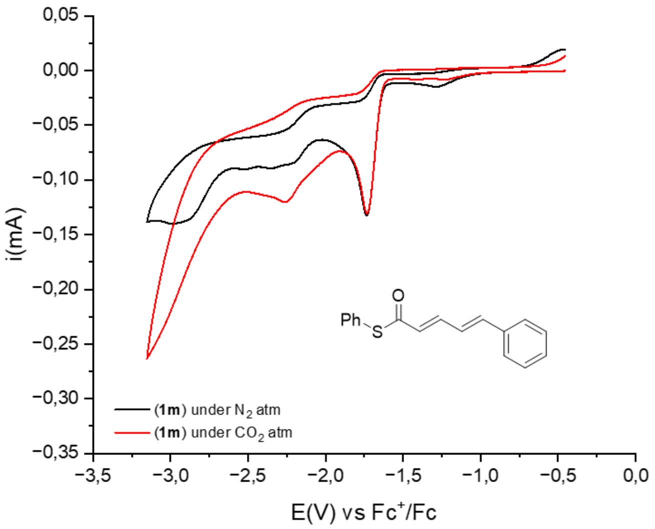

Figure S14. Cyclic voltammetry of **1m** (1 mM in CH_3CN) under N_2 (black trace) and CO_2 (red trace) atmosphere.

C.14 CV OF (1N)

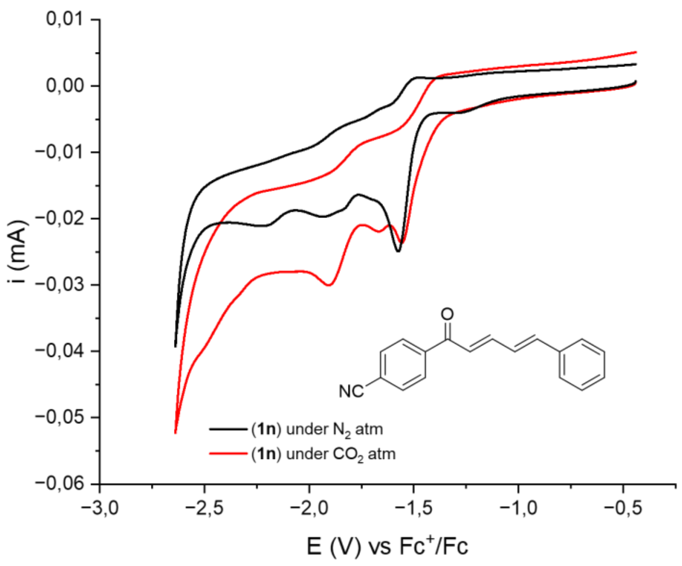

Figure S15. Cyclic voltammetry of **1n** (1 mM in CH_3CN) under N_2 (black trace) and CO_2 (red trace) atmosphere.

C.15 CV OF (1O)

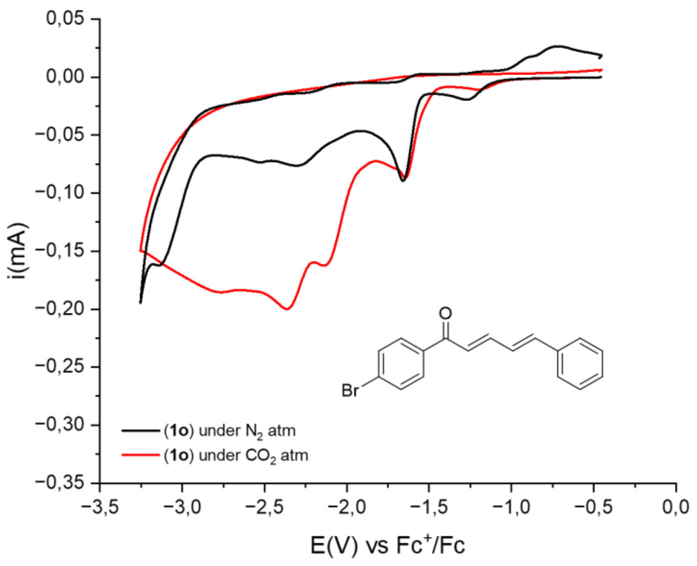

Figure S16. Cyclic voltammetry of **1o** (1 mM in CH_3CN) under N_2 (black trace) and CO_2 (red trace) atmosphere.

C.14 CV OF (1P)

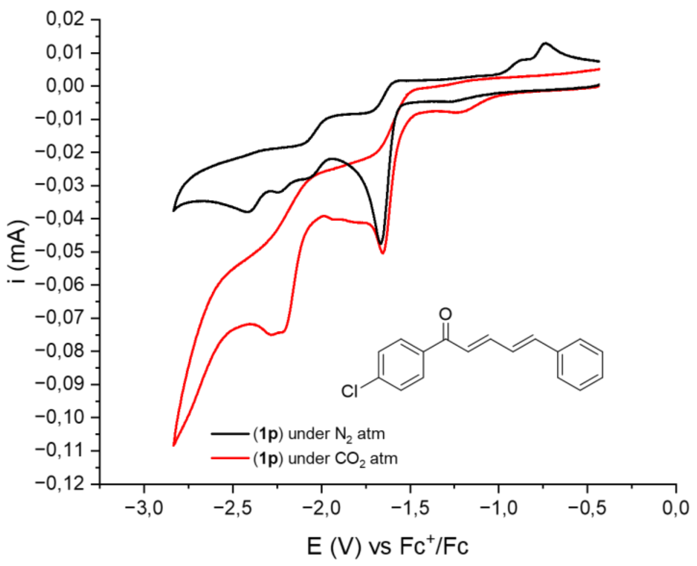

Figure S17. Cyclic voltammetry of **1p** (1 mM in CH_3CN) under N_2 (black trace) and CO_2 (red trace) atmosphere.

C.15 CV OF (1Q)

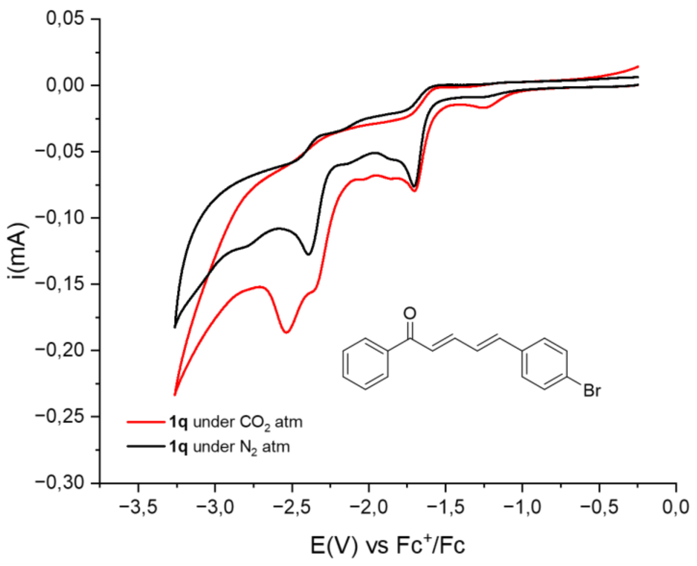

Figure S18. Cyclic voltammetry of **1q** (1 mM in CH_3CN) under N_2 (black trace) and CO_2 (red trace) atmosphere.

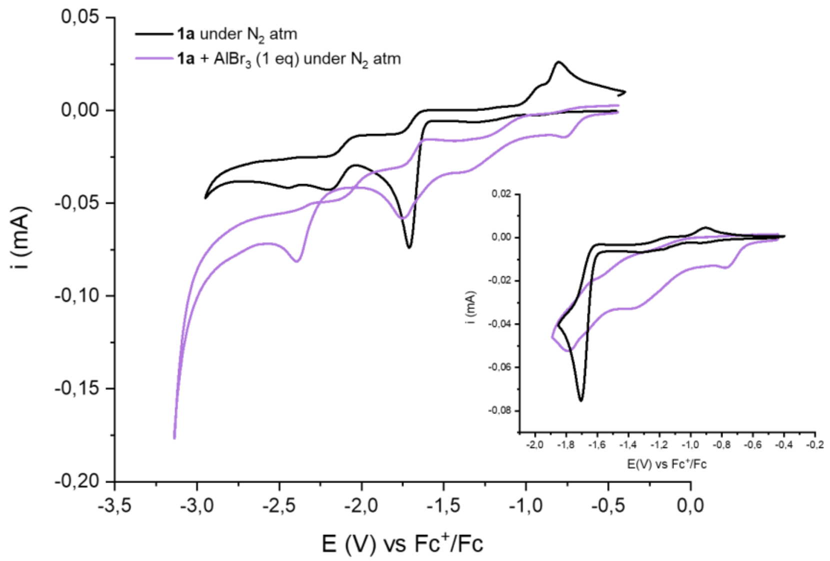

Figure S19. Cyclic voltammetry of **1a** (5 mM in CH_3CN) (black trace) and with 1 equivalent of $AlBr_3$ (violet trace) under N_2 atmosphere.

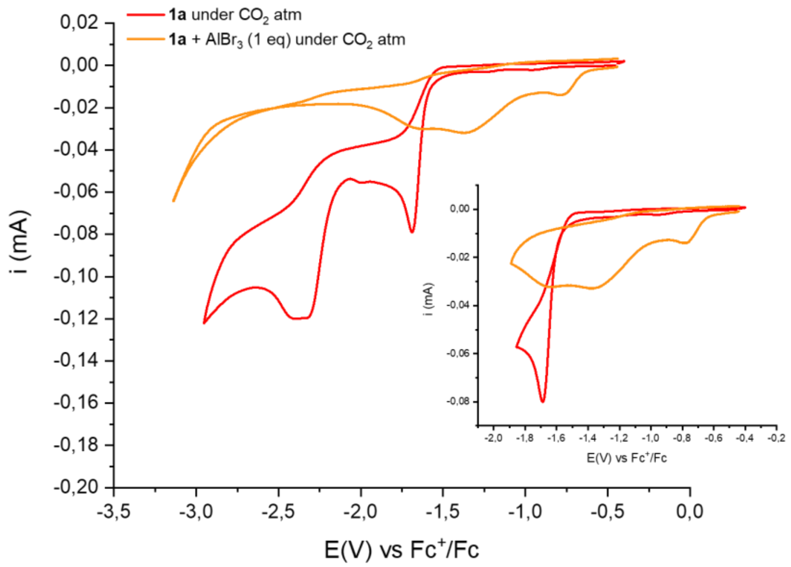

Figure S20. Cyclic voltammetry of **1a** (5 mM in CH_3CN) (black trace) and with 1 equivalent of $AlBr_3$ (yellow trace) under CO_2 atmosphere.

D. ELECTROCHEMICAL CARBOXYLATION OF CHALCONE

D.1 CV OF CHALCONE (S8)

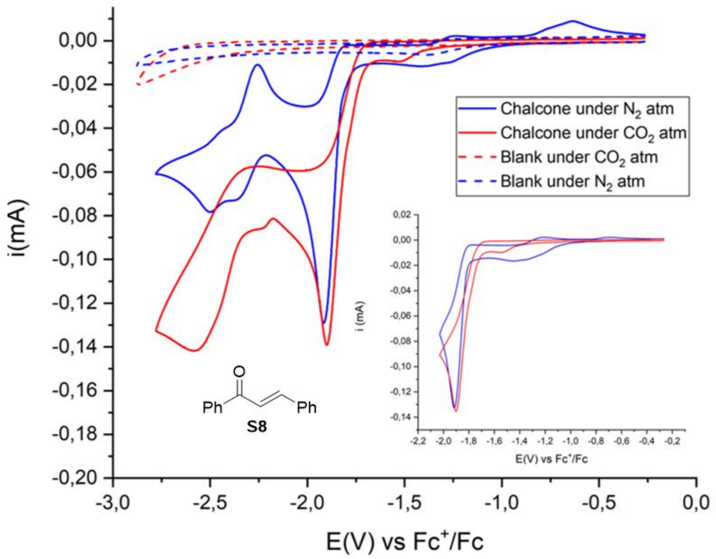

Figure S21. Cyclic voltammetry of **S8** (5 mM in CH_3CN) under N_2 (black trace) and CO_2 (red trace) atmosphere.

D.2 ELECTROCHEMICAL CARBOXYLATION

Constant potential electrolysis experiment was preliminarily performed on compound **S8** to ensure the applicability of the protocol to the compound **1a-q**. Further details on the electrochemical set-up are described below (**Procedure E**). The experiment started with an initial current of ca – 25 mA and was stopped at Q= −61 C (2 F/mol).

At the end of the reaction, the electrolysis solution was evaporated under vacuum, acidified with 2 mL of HCl (1 M) and extracted with EtOAc. The organic phase was dried with $MgSO_4$ and then concentrated under vacuum. NMR yield was obtained adding CH_2Br_2 as internal standard. Carboxylation products were purified by chromatographic column (petroleum ether : ethyl acetate).

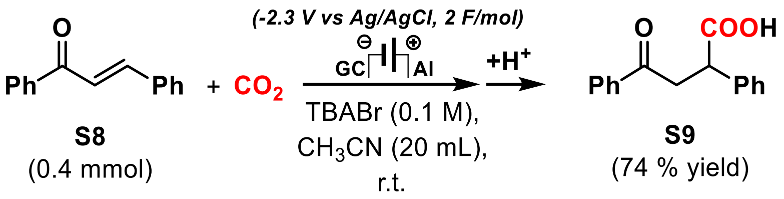

Scheme S1. Electrochemical carboxylation of **S8**.

Characterization data

4-oxo-2,4-diphenylbutanoic acid

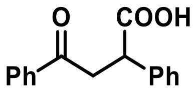

S9

S9 was synthesized according to the described electrosynthesis as a white solid (88 mg, 74% yield). **^{1}H-NMR** (300 MHz, $CDCl_3$): δ 7.94 (d, *J* = 7.6 Hz, 2H), 7.53 (d, *J* = 7.3 Hz, 1H), 7.43 (t, *J* = 7.6 Hz, 2H), 7.39 – 7.21 (m, 5H), 4.31 (dd, *J* = 10.0, 4.3 Hz, 1H), 3.89 (dd, *J* = 18.1, 10.0 Hz, 1H), 3.28 (dd, *J* = 18.0, 4.3 Hz, 1H) ppm. **^{13}C-NMR** (101 MHz, $CDCl_3$): δ 197.5, 179.1, 137.8, 136.4, 133.5, 129.1 (x2), 128.7 (x2), 128.2 (x2), 128.1 (x2), 127.9, 46.4, 42.4 ppm. The characterization data matched with the reported one.[22]

E. REACTION OPTIMIZATION

Optimized reaction procedure is descried in the following section. In Table S1, there is a sum-up of all the reaction optimisation experiments.

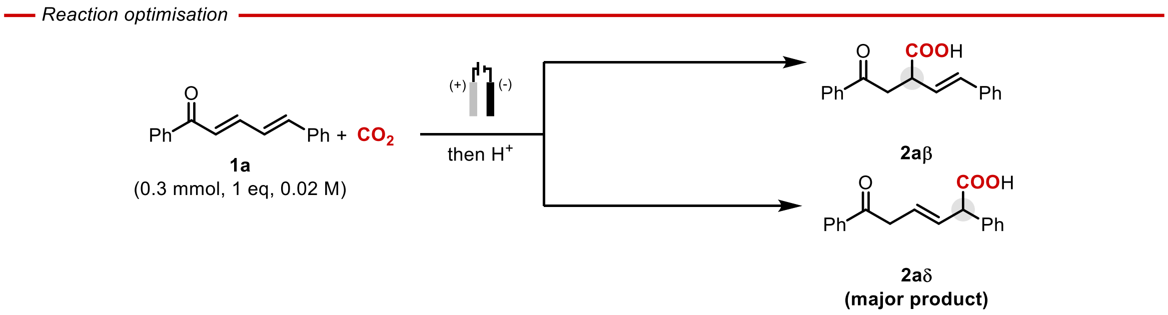

entry	CPE (E_{WE}, V)	CCE (i,mA)	Q (F/mol1_a)	cathode	anode	supporting electrolyte	variations	solvent	T (°C)	yield [a] 1aδ:1aβ (%)	s
1	-2.46	-	2	GC	Al	TBABr	-	ACN	r.t.	32:4	8:1
2	-2.46	-	2	GC	Al	TBABr	-	ACN	0	42:4	10.5:1
**3**	**-2.46**	**-**	**2**	**GC**	**Al**	**TBABr**	**-**	**ACN**	**-15**	**50:5**	**10:1**
4	-2.46	-	2	GC	Al	TBABr	-	ACN	-41	41:4	10.2:1
5	-2.46	-	2	GC	Al	TBABr	-	ACN	58	13:2	6.5:1
6	-2.46	-	1.6	GC	Al	TEABr	**1a** (0.05 M)	ACN	-15	32:4	8:1
7	-2.46	-	2	GC	Al	TBABr	TBD (0.5 eq)	ACN	0	19:3	6.3:1
8	-2.46	-	2	GC	Al	TBABr	-	DMF	0	11:4	2.7:1
9	-2.46	-	2	GC	Al	TBABr	-	THF	-15	n.d.	-
10[b]	-	10	4	GC	Al	TBABr	-	ACN	0	23:2	11.5:1
11[b]	-	10	4	Pt	Al	TBABr	-	ACN	0	n.d.	-
12[b]	10	-	2.5	SS	GC	TEAI	-	DMF	r.t.	n.d.	-
13	-2.46	-	1.5	SS	Al	TEABr	-	ACN	-15	42:4	10:1
14	-2.46	-	2	Ni	Al	TEABr	-	ACN	-15	7:3	2.3:1
15	-2.46	-	2	GC	SS	TEABr	-	ACN	-15	14:2	7:1
16	-2.46	-	1.7	GC	Zn	TEABr	-	ACN	-15	34:4	8.5:1
17	-2.46	-	2	GC	GC	TEABr	TEA (5 eq.)	ACN	-15	n.d.	-
**18**	**-2.46**	**-**	**2**	**GC**	**Al**	**TEABr**	**-**	**ACN**	**-15**	**(44-50):(4-5)**	**10:1**
**19**	**-2.46**	**-**	**2**	**GC**	**Al**	**TEABr**	**H_2O (2.5 eq)**	**ACN**	**-15**	**44:4**	**11:1**
20	-2.46	-	2	GC	Al	TEAI	-	ACN	0	n.d.	-
21[c]	-1.86	-	1.4	GC	Al	TEABr	-	ACN	-15	(31-33):(3-5)	8:1
22[d]	-2.46	-	0.1	GC	Al	TEABr	$AlBr_3$ (1 eq)	ACN	-15	n.d.	-
23	-2.46	-	2	GC	Al	TEABr	TMSCl (1 eq)	ACN	-15	11:n.d	-
24	-2.46	-	2	GC	Al	TEABr	no electricity	ACN	-15	n.d.	-
25	-2.46	-	2	GC	Al	TEABr	N_2, no CO_2	ACN	-15	n.d.	-

Table S1. Experiments for electrochemical carboxylation of **1a,** included in the reaction optimisation Table 1 (main text). [a]Determined by ^{1}H-NMR with CH_2Br_2 as internal standard after acidification (1M HCl) and extraction (EtOAc). [b] 2-electrode electrochemical cell set up. [c]Range of yields obtained from duplicated,

repeated experiments. Reaction conducted also with the consumption of 1 F/mol$_{\mathbf{1a}}$ yields the same results. [d]Reaction stopped because of rapid passivation of working electrode, probably due to the reduction of some aluminium complexes with substrates Al(**1a**)$_x^{3+}$.

E.1 2-ELECTRODE ELECTROCHEMICAL CELL SET UP

Constant potential electrolysis at 10 V and constant current electrolysis experiments were performed in a custom-made 1-necked glass cell under stirring conditions of the solution with a magnetic stirring bar.

The electrodes employed were: a homemade Pt-coated SS-grid, Al wire counter electrode (Sigma, 1.0 mm, 99.999%), glassy carbon plate working electrode (SIGRADUR, HTW, ca 1 cm^2 geometric area), a stainless steel rod 316L (ca 1 cm^2 geometric area).

An oven-dried electrochemical cell was fully equipped with the electrodes (see **Table S1**, for specific entries), filled with electrolyte solution and the substrate **1a**. The electrode was accommodated in the cell by a PTFE septum (**Figure S22**).

The cell was then placed in an ice batch at the medium temperature of 0 °C or leaved at room temperature (see **Table S1**, for specific entries). After saturating the electrolyte solution with CO_2 for ca 10 min, the specific potential or current was applied, and the CO_2 atmosphere was kept above the solutions during the electrolysis experiments.

The experiment was stopped at the consumption of the relative Q (2 F/mol) (see **Table S1**, for specific entries).

At the end of the reaction, the electrolysis solution was evaporated under vacuum, acidified with 2 mL of HCl (1 M) and extracted with EtOAc. The organic phase was dried with $MgSO_4$ and then concentrated under vacuum. NMR yield was obtained adding CH_2Br_2 as internal standard.

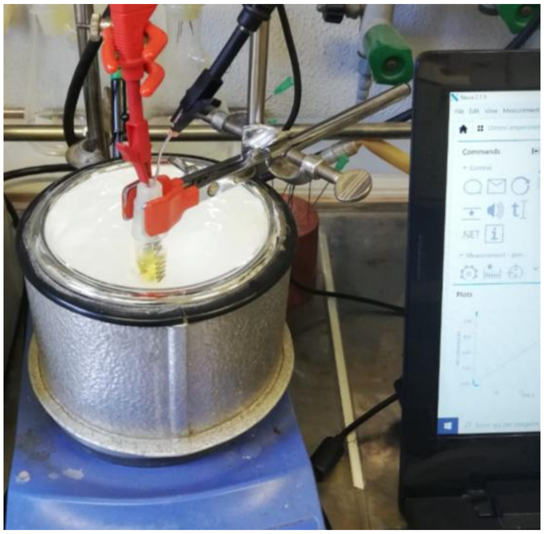

Figure S22. Example of the 2-electrode electrochemical cell set up.

F. ELECTROCHEMICAL CARBOXYLATION PROCEDURES

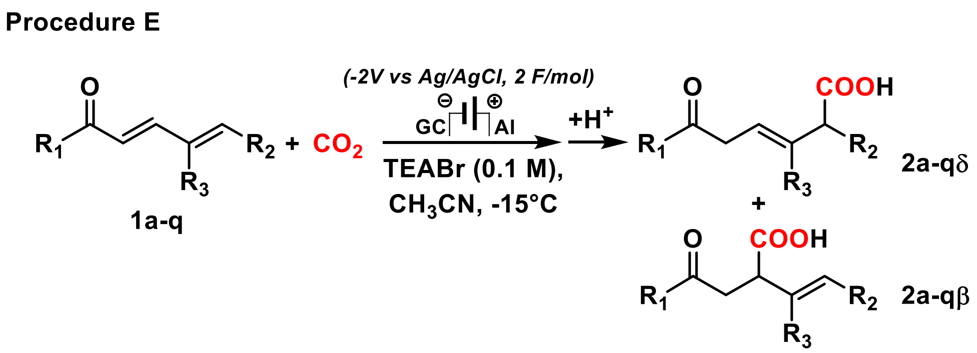

Scheme S2. Electrochemical carboxylation of dienones.

Constant potential electrolysis experiments were performed in a custom-made 4-necked glass cell under stirring conditions of the solution with a magnetic stirring bar.

An oven-dried electrochemical cell was fully equipped with a glassy carbon rod working electrode (SIGRADUR, HTW, ca 1.5 cm^2 geometric area), an Ag/AgCl (NaCl 3 M) – separated from the bulk solution by means of a salt bridge (equipped with a Coralpor frit), filled with electrolyte solution – as reference electrode and an Al wire counter electrode (Sigma, 1.0 mm Ø, 99.999%). Each electrode was accommodated in the cell by a PTFE O-ring and secured with a screw cap (**Figure S23**).

Other electrodes employed during the reaction optimization were: a Ni foam electrode (Sigma-Aldrich), stainless steel (SS) rod 316L (used as cathode, ca 1 cm^2 geometric area), a SS wire (used as anode, 316L SS, 0.6 mm Ø), a Zn wire (Carlo Erba, 1.0 mm Ø).

Prior to use, the Al, SS, and Zn electrodes were activated by immersing the wires in a 6 M HCl solution for approximately 15 minutes, followed by thorough rinsing with water and then with acetone.

The substrates **1a-p** (0.3 mmol, 1 eq) and the supporting electrolyte tetraethylammonium bromide, TEABr, 1.5 mmol, 5 eq) were added to the electrochemical cell. Then the cell was filled with anhydrous CH_3CN♣. The cell was then placed in a NaCl-ice batch at the medium temperature of -15 °C♣♣. After saturating the electrolyte solution with CO_2 for ca 20 min, the potential of -2 V vs Ag/AgCl was applied, and the CO_2 atmosphere was kept above the solutions during the electrolysis experiments.

The experiment generally started with an initial current of ca – 20 mA and was stopped at Q= −58 C (2 F/mol), after ca 2h (**Figure S24**).

♣ In the case of **1o-q** reaction was also repeated with anhydrous DMF and anhydrous mixture of DMF:ACN (1.5:1) at 0°C.

♣♣ In the case of **1p**, reaction was conducted under the optimized reaction conditions at room temperature instead of -15°C.

At the end of the reaction, the electrolysis solution was evaporated under vacuum, acidified with 2 mL of HCl (1 M) and extracted with EtOAc. The organic phase was dried with $MgSO_4$ and then concentrated under vacuum. NMR yield was obtained adding CH_2Br_2 as internal standard. Carboxylation products were directly injected in a preparative HPLC (flow rate = 17 mL/min, H_2O:CH_3CN gradient = 95:5 → 0:100 in 50 min).

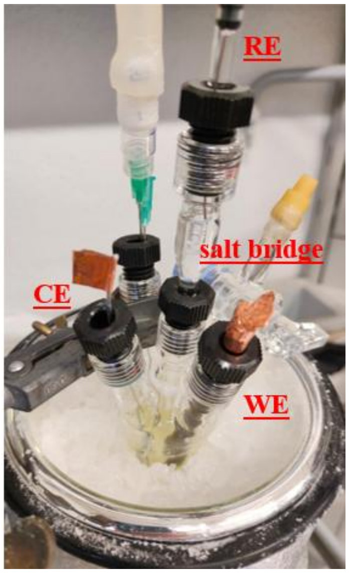

Fig. S23. The single compartment cell used for electrosynthesis experiments.

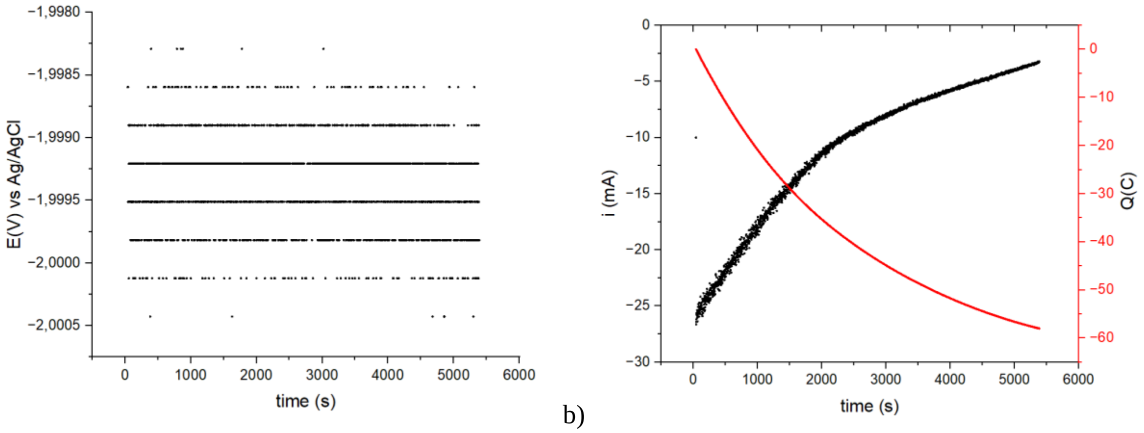

a) b)

Fig. S24. a) Potential (E, V) and b) Current (i, mA) / Charge (Q, C) profiles generally observed during the electrosynthesis experiments.

G. CHARACTERIZATION DATA

The nature of the carboxylation product **2aδ** was confirmed by multiple NMR techniques (^{1}H-NMR, ^{13}C-NMR) and mass spectrometry analysis. In particular, the structure of the major product **2aδ** was undoubtedly confirmed by Heteronuclear Multiple Bond Correlation (HMBC) experiment (**Figure S25**) and 2D-COSY, 2D-NOESY. The main ^{1}J correlations observed are those between H in blue (d_H 4.38) and C in grey (d_C 54.1), between the methylene hydrogens in violet (d_H 3.84) and the C in purple (d_C 41.7), while ^{2}J correlations are observed between H in green and orange (d_H 6.01 and 5.83, respectively) with the two carbons of the unsaturated bond (d_C 126.2 and 131.4, respectively). A further analysis of long-range correlations for hydrogen in blue and for methylene hydrogens in violet is diagnostic for the confirmation of the structure. In the case of H in blue (d_H 4.38), long-range correlations are observed with C in red (d_C 173.1: ^{2}J), with C of the phenyl ring (d_C 139.0: ^{2}J) and with the carbons of the double bond (d_C 126.2: ^{2}J and d_C 131.4: ^{3}J). As expected, no ^{4}J correlation is observed between this hydrogen and the carbon in purple (d_C 41.7). Analogously, the hydrogens in violet (d_H 3.84) give long range correlations with the carbon in brown of C=O (d_C 198.0: ^{2}J), and with the carbons of the double bond (d_C 131.4: ^{2}J and d_C 126.2: ^{3}J), while very weak correlation is observed with C in grey (d_C 54.1: ^{4}J).

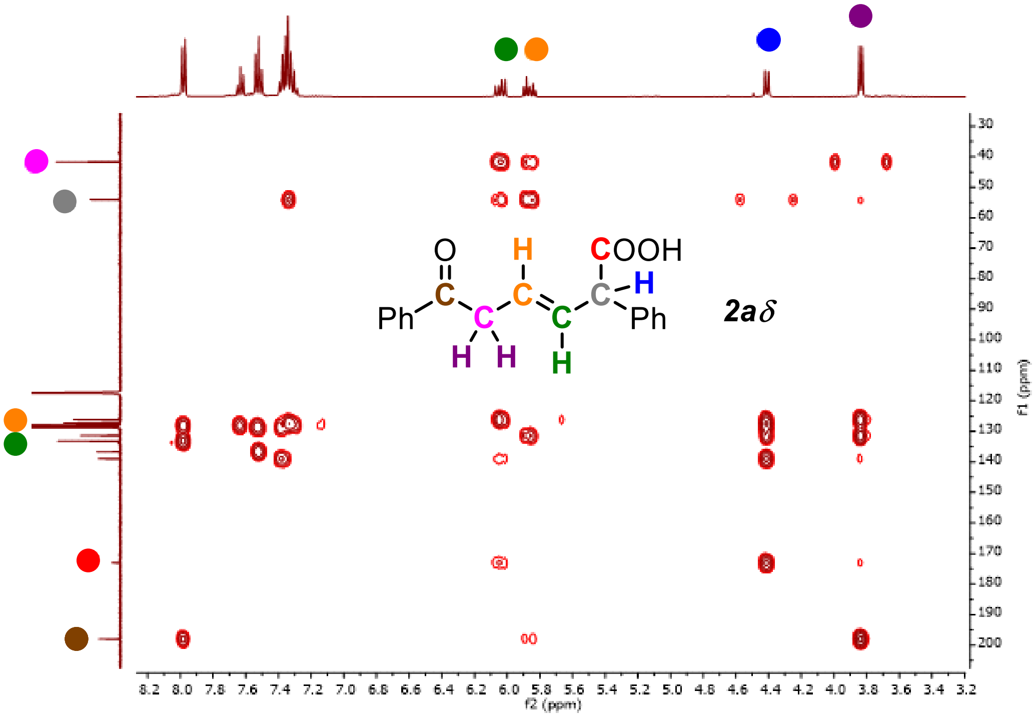

Figure S25. ^{1}H – ^{13}C HMBC spectrum of **2aδ** in CD_3CN. Nuclei giving diagnostic short and long range correlations peaks are highlighted with different colours in the structure, with the same colour code in the circles indicating the resonances on the one-dimensional projection spectra (^{1}H: horizontal axes, ^{13}C: vertical axes).

Characterization data

(*E*)-6-oxo-2,6-diphenylhex-3-enoic acid

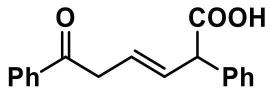

2aδ

2aδ was synthesized according to the electrosynthesis **Procedure E** as a white solid. NMRy: 50%. **^{1}H-NMR** (400 MHz, Acetonitrile-d_3): δ 8.04 – 7.88 (m, 2H), 7.65 – 7.55 (m, 1H), 7.49 (dd, J = 8.4, 7.0 Hz, 2H), 7.39 – 7.22 (m, 5H), 6.01 (ddt, J = 15.6, 8.5, 1.4 Hz, 1H), 5.83 (dt, J = 15.4, 6.7 Hz, 1H), 4.38 (d, J = 8.4 Hz, 1H), 3.84 – 3.75 (m, 2H) ppm. **^{13}C-NMR** (101 MHz, Acetonitrile-d_3): δ 198.0, 173.1, 138.9, 136.6, 133.1, 131.3, 128.7 (x2), 128.6 (x2), 128.1(x2), 127.9 (x2), 127.2, 126.1, 54.0, 41.7 ppm. **HRMS (ESI-MS)** calculated for $C_{18}H_{17}O_3^+$ $[M+H]^+$ 281.1172, found 281.1170.

(*E*)-2-(2-oxo-2-phenylethyl)-4-phenylbut-3-enoic acid

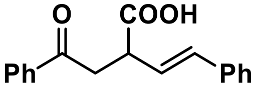

2aβ

2aβ was synthesized according to the electrosynthesis **Procedure E**. NMRy: 5%. The final (E)-6-oxo-2,6-diphenylhex-3-enoic acid (**2a**) was obtained in traces and as a mixture with unknown compound. **^{1}H-NMR** (400 MHz, Chloroform-d) 8.10 – 7.88 (m, 1H) δ 7.65 – 7.32 (m, 8H), 6.65 (d, J = 15.9 Hz, 1H), 6.31 (dd, J = 15.9, 8.4 Hz, 1H), 3.69 (dd, J = 17.9, 8.9 Hz, 1H), 3.32 (dd, J = 18.0, 4.6 Hz, 1H), 2.35 (t, J = 7.5 Hz, 1H) ppm.

(*E*)-6-(4-methoxyphenyl)-6-oxo-2-phenylhex-3-enoic acid

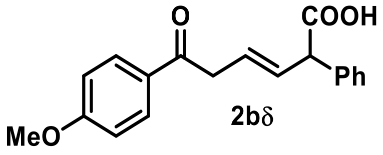

2bδ

2bδ was synthesized according to the electrosynthesis **Procedure E** as a white solid. NMRy: 38%. **^{1}H-NMR** (400 MHz, Acetonitrile-d$_3$): δ 7.92 (d, *J* = 8.9 Hz, 2H), 7.46 – 7.13 (m, 5H), 7.01 – 6.93 (m, 2H), 6.00 (ddt, *J* = 15.5, 8.4, 1.4 Hz, 1H), 5.91 – 5.74 (m, 1H), 4.38 (d, *J* = 8.4 Hz, 1H), 3.84 (s, 3H), 3.77 – 3.67 (m, 2H) ppm. **^{13}C-NMR** (101 MHz, Acetonitrile-d$_3$): δ 197.4, 174.2, 164.6, 139.9, 132.1, 131.4 (x2), 130.5, 129.7 (x2), 128.9 (x2), 128.2, 127.5, 114.7(x2), 56.3, 55.0, 42.4.ppm. **HRMS (ESI-MS)** calculated for $C_{19}H_{18}O_4Na^+$ [M+Na]$^+$ 333.1097, found 333.1107.

(*E*)-6-oxo-2-phenyl-6-(4-(trifluoromethyl)phenyl)hex-3-enoic acid

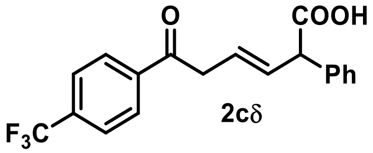

2cδ

2cδ was synthesized according to the electrosynthesis **Procedure E** (in this case E_{WE}= -1.8V vs Ag/AgCl was used to better ensure the dianion formation) as a yellow oil. NMRy: 25%. **^{1}H-NMR** (400 MHz, Acetonitrile-d$_3$): δ 8.08 (d, *J* = 8.1 Hz, 2H), 7.80 (d, *J* = 8.2 Hz, 2H), 7.42 – 7.24 (m, 5H), 6.03 (dd, *J* = 15.5, 8.4 Hz, 1H), 5.83 (dt, *J* = 15.5, 6.7 Hz, 1H), 4.39 (d, *J* = 8.4 Hz, 1H), 3.85 (d, *J* = 6.6 Hz, 2H) ppm. **^{19}F NMR** (377 MHz, Acetonitrile-*d*$_3$) δ -63.59 ppm.**^{13}C-NMR** (151 MHz, Acetonitrile-d$_3$): δ 198.2, 173.9, 140.5, 139.7, 134.3 (q, *J* = 32.3 Hz), 132.7, 129.6 (x4), 128.8 (x2), 128.1, 126.5 (x2) (q, *J* = 3.8 Hz), 126.4,124.8 (q, *J* = 271.7 Hz) 54.9, 42.8 ppm. **HRMS (ESI-MS)** calculated for $C_{19}H_{16}F_3O_3^+$ [M+H]$^+$ 349.1046, found 349.1048.

(*E*)-6-(naphthalen-2-yl)-6-oxo-2-phenylhex-3-enoic acid

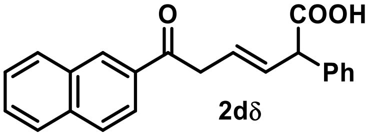

2dδ

2dδ was synthesized according to the electrosynthesis **Procedure E** (in this case E_{WE}= -1.9V vs Ag/AgCl was used to better ensure the dianion formation) as a white solid. NMRy: 52%. **^{1}H-NMR** (600 MHz, Acetonitrile-d_3): δ 8.53 (d, *J* = 1.8 Hz, 1H), 7.99 (d, *J* = 8.1 Hz, 1H), 7.94 (dd, *J* = 8.6, 1.8 Hz, 1H), 7.90 (dd, *J* = 8.6, 2.2 Hz, 2H), 7.61 (ddd, *J* = 8.2, 6.9, 1.3 Hz, 1H), 7.56 (ddd, *J* = 8.0, 6.8, 1.3 Hz, 1H), 7.34 – 7.26 (m, 4H), 7.24 (td, *J* = 6.0, 2.6 Hz, 1H), 6.09 – 5.97 (m, 1H), 5.86 (dtd, *J* = 15.5, 6.8, 0.9 Hz, 1H), 4.37 (d, *J* = 8.5 Hz, 1H), 3.88 (dd, *J* = 6.8, 1.3 Hz, 2H) ppm. **^{13}C-NMR** (151 MHz, Acetonitrile-d_3): δ 198.9, 174.1, 139.8, 136.3, 134.8, 133.4, 132.3, 131.0, 130.4, 129.6 (x2), 129.5, 129.2, 128.8 (x2), 128.5, 128.1, 127.8, 127.1, 124.5, 55.0, 42.7 ppm. **HRMS (ESI-MS)** calculated for $C_{22}H_{19}O_3H^+$ $[M+Na]^+$ 311.1329, found 311.1445.

(*E*)-6-(furan-2-yl)-6-oxo-2-phenylhex-3-enoic acid

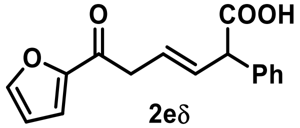

2eδ

2eδ was synthesized according to the electrosynthesis **Procedure E** as a yellow oil. NMRy: 40%. **^{1}H-NMR** (400 MHz, Acetonitrile-d_3): δ 7.71 (s, 1H), 7.32 (ddd, *J* = 16.4, 12.0, 5.3 Hz, 6H), 6.59 (dd, *J* = 3.6, 1.9 Hz, 1H), 6.04 (dd, *J* = 15.5, 8.5 Hz, 1H), 5.77 (dt, *J* = 14.9, 6.8 Hz, 1H), 4.37 (d, *J* = 8.5 Hz, 1H), 3.61 (d, *J* = 6.9 Hz, 2H) ppm. **^{13}C-NMR** (101 MHz, Acetonitrile-d_3): 187.3, 173.9, 152.9, 148.2, 139.7, 132.6, 129.6 (x2), 128.8 (x2), 128.1, 126.3, 119.0, 113.1, 54.8, 42.4 ppm. **HRMS (ESI-MS)** calculated for $C_{16}H_{15}O_4^+$ $[M+H]^+$ 271.0965, found 271.0965.

(*E*)-2-(2-(furan-2-yl)-2-oxoethyl)-4-phenylbut-3-enoic acid

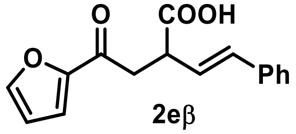

2eβ

2eβ was synthesized according to the electrosynthesis **Procedure E** as a white solid. NMRy: 10%. **^{1}H-NMR** (400 MHz, Acetonitrile-d_3): δ 7.73 (s, 1H), 7.40 (d, *J* = 7.7 Hz, 2H), 7.36 – 7.29 (m, 3H), 7.29 – 7.23 (m, 1H), 6.64 – 6.56 (m, 2H), 6.32 (dd, *J* = 15.9, 8.1 Hz, 1H), 3.75 (td, *J* = 8.5, 5.1 Hz, 1H), 3.44 (dd, *J* = 17.5, 8.9 Hz, 1H), 3.13 (dd, *J* = 17.5, 5.1 Hz, 1H) ppm. **^{13}C-NMR** (101 MHz, Acetonitrile-d_3): δ 187.3, 174.3, 153.1, 148.2, 137.6, 133.3, 129.6 (x2), 128.6, 127.3, 127.1 (x2), 118.8, 113.2, 44.3, 40.8 ppm. **HRMS (ESI-MS)** calculated for $C_{16}H_{15}O_4^+$ $[M+H]^+$ 271.0965, found 271.0965.

(*E*)-6-oxo-2-phenylhept-3-enoic acid

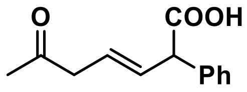

2fδ

2fδ was synthesized according to the electrosynthesis **Procedure E** as a pale-yellow oil. NMRy: 56%. **^{1}H-NMR** (400 MHz, Acetonitrile-d_3): δ 7.51 – 7.14 (m, 5H), 5.92 (dd, J = 15.5, 8.4 Hz, 1H), 5.68 (dt, J = 14.8, 7.0 Hz, 1H), 4.34 (d, J = 8.4 Hz, 1H), 3.20 (d, J = 7.0 Hz, 2H), 2.06 (s, 3H) ppm. **^{13}C-NMR** (101 MHz, Acetonitrile-d_3): δ 207.3, 174.0, 139.9, 132.3, 129.7, 128.9 (x2), 128.2 (x2), 126.8, 47.2, 29.7 ppm. **HRMS (ESI-MS)** calculated for $C_{13}H_{15}O_3^+$ $[M+H]^+$ 219.1016, found 219.1150.

(*E*)-2-(4-methoxyphenyl)-6-oxo-6-phenylhex-3-enoic acid

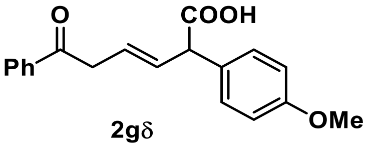

2gδ

2gδ was synthesized according to the electrosynthesis **Procedure E** as a light-red solid. NMRy: 54% **^{1}H-NMR** (400 MHz, Acetonitrile-d_3): δ 8.09 – 7.80 (m, 2H), 7.64 – 7.57 (m, 1H), 7.49 (dd, J = 8.3, 7.1 Hz, 2H), 7.21 (d, J = 8.7 Hz, 2H), 6.89 (d, J = 8.7 Hz, 2H), 6.04 – 5.92 (m, 1H), 5.80 (dt, J = 15.3, 6.7 Hz, 1H), 4.32 (d, J = 8.3 Hz, 1H), 3.80 (d, J = 6.7 Hz, 2H), 3.77 (s, 3H). **^{13}C-NMR** (101 MHz, Acetonitrile-d_3): δ 198.7, 173.0, 145.1, 137.5, 134.1, 133.4 (x2), 131.06, 129.9 (x2), 129.6 (x2), 129.0 (x2), 128.4, 119.5, 111.7, δ 54.8, 42.5. **HRMS (ESI-MS)** calculated for $C_{19}H_{19}O_4^+$ $[M+H]^+$ 311.1278, found 311.1274.

(*E*)-4-(4-methoxyphenyl)-2-(2-oxo-2-phenylethyl)but-3-enoic acid

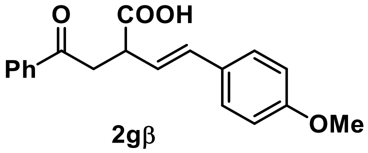

2gβ

2gβ was synthesized according to the electrosynthesis **Procedure E** as a white solid. NMRy: 13% **^{1}H-NMR** (400 MHz, Acetonitrile-d_3): δ 7.99 (d, *J* = 7.6 Hz, 2H), 7.61 (t, *J* = 7.4 Hz, 1H), 7.50 (t, *J* = 7.7 Hz, 2H), 7.32 (d, *J* = 8.2 Hz, 2H), 6.86 (d, *J* = 8.2 Hz, 2H), 6.55 (d, *J* = 15.9 Hz, 1H), 6.18 (dd, *J* = 15.9, 8.1 Hz, 1H), 3.76 (s, 3H), 3.73 (d, *J* = 4.0 Hz, 1H), 3.60 (dd, *J* = 17.8, 9.0 Hz, 1H), 3.26 (dd, *J* = 17.9, 4.5 Hz, 1H). **^{13}C-NMR** (75 MHz, Acetonitrile-d_3): δ 199.0, 175.10, 160.3, 137.6, 134.2, 132.6, 130.4, 129.6 (x3), 128.9 (x2), 128.4 (x3), 125.3, 55.86, 44.9, 41.6. **HRMS (ESI-MS)** calculated for $C_{19}H_{17}O_4^-$ $[M-H]^-$ 309.1132, found 309.1131.

(*E*)-2-(4-cyanophenyl)-6-oxo-6-phenylhex-3-enoic acid

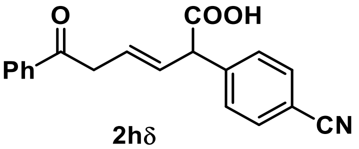

2hδ

2hδ was synthesized accordinfg to the electrosynthesis **Procedure E** as a pale-yellow oil. NMRy: 31% **^{1}H-NMR** (400 MHz, Acetonitrile-d_3): δ 7.97 – 7.85 (m, 2H), 7.69 – 7.61 (m, 2H), 7.61 – 7.55 (m, 1H), 7.50 – 7.42 (m, 4H), 5.95 (dd, *J* = 15.6, 8.1 Hz, 1H), 5.85 (dt, *J* = 15.6, 6.5 Hz, 1H), 4.47 (d, *J* = 8.1 Hz, 1H), 3.79 (d, *J* = 6.2 Hz, 2H). **^{13}C-NMR** (75 MHz, Acetonitrile-d_3): δ 198.8, 173.0f, 159.85, 137.62, 134.09, 132.57, 131.80, 129.94 (x2), 129.61 (x2), 129.03 (x2), 126.76, 114.97 (x2), 55.84, 54.08, 42.67. **HRMS (ESI-MS)** calculated for $C_{19}H_{16}NO_3^+$ $[M+H]^+$ 306.1125, found 306.1126.

(*E*)-2-ethyl-6-oxo-6-phenylhex-3-enoic acid

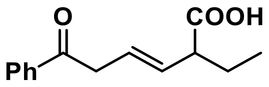

2iδ

2iδ was synthesized according to the electrosynthesis **Procedure E** as a pale-yellow oil. NMRy: 24% **^{1}H-NMR** (400 MHz, Acetonitrile-d_3): δ 8.02 – 7.89 (m, 2H), 7.65 – 7.56 (m, 1H), 7.50 (dd, *J* = 8.2, 6.8 Hz, 2H), 5.80 (dt, *J* = 15.4, 6.7 Hz, 1H), 5.59 (dd, *J* = 15.6, 8.6 Hz, 1H), 3.78 (dd, *J* = 6.7, 1.3 Hz, 2H), 2.93 (q, *J* = 7.7 Hz, 1H), 1.72 (dp, *J* = 14.4, 7.3 Hz, 1H), 1.51 (dp, *J* = 14.7, 7.4 Hz, 1H), 0.87 (t, *J* = 7.4 Hz, 3H). **^{13}C-NMR** (101 MHz, Acetonitrile-d_3): 199.1, 175.7, 137.5, 134.0, 132.55, 129.5 (x2), 129.0 (x2), 126.7, 51.1, 42.7, 26.2, 11.7. **HRMS (ESI-MS)** calculated for $C_{14}H_{17}O_3^+$ $[M+H]^+$ 233.1172, found 233.1193.

(*E*)-2-(2-oxo-2-phenylethyl)hex-3-enoic acid

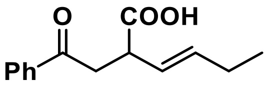

2iβ

2iβ was synthesized according to the electrosynthesis **Procedure E** as a white solid. NMRy: 24% **^{1}H-NMR** (400 MHz, Acetonitrile-d_3): δ 8.04 – 7.88 (m, 2H), 7.62 (td, *J* = 7.2, 1.4 Hz, 1H), 7.56 – 7.39 (m, 2H), 5.80 – 5.68 (m, 1H), 5.53 (ddt, *J* = 15.4, 7.6, 1.5 Hz, 1H), 3.60 – 3.45 (m, 2H), 3.22 – 3.10 (m, 1H), 2.11 – 1.98 (m, 2H), 0.96 (d, *J* = 14.9 Hz, 3H). **^{13}C-NMR** (101 MHz, Acetonitrile-d_3): δ 199.0, 175.2, 137.6, 136.2, 134.1, 129.6 (x2), 128.8 (x2), 126.4, 44.4, 41.5, 26.1, 13.7. **HRMS (ESI-MS)** calculated for $C_{14}H_{16}NaO_3^+$ $[M+Na]^+$ 255.0992, found 255.0990.

(*Z*)-6-oxo-2,3,6-triphenylhex-3-enoic acid

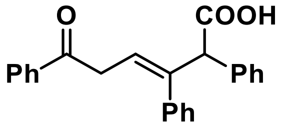

2jδ

2jδ was synthesized according to the electrosynthesis **Procedure E** as a white solid. NMRy: 36%. **^{1}H-NMR** (400 MHz, Acetonitrile-d_3): δ 7.82 – 7.75 (m, 2H), 7.62 – 7.54 (m, 1H), 7.43 (t, J = 7.7 Hz, 2H), 7.39 – 7.24 (m, 8H), 7.20 – 7.13 (m, 2H), 5.87 (td, J = 7.0, 1.4 Hz, 1H), 4.80 (s, 1H), 3.66 (ddd, J = 7.3, 2.8, 1.0 Hz, 2H). **^{13}C-NMR** (101 MHz, Acetonitrile-d_3): δ 198.9, 173.4, 142.4, 140.3, 137.9, 137.6, 134.1, 130.3 (x2), 129.7 (x2), 129.5 (x2), 129.3 (x2), 129.2 (x2), 128.9 (x2), 128.4, 128.3, 59.9, 39.8. **HRMS (ESI-MS)** calculated for $C_{24}H_{21}O_3^+$ $[M+H]^+$ 357.1485, found 357.1481.

(*E*)-6-ethoxy-6-oxo-2-phenylhex-3-enoic acid

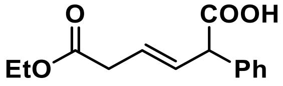

2kδ

2kδ was synthesized according to the electrosynthesis **Procedure E** (in this case E_{WE}= -1.9V vs Ag/AgCl was used to better ensure the dianion formation) as a pale-yellow oil. NMRy: 21% **^{1}H-NMR** (400 MHz, Acetonitrile-d_3): δ 7.37 – 7.25 (m, 5H), 5.96 (ddt, *J* = 15.4, 8.4, 1.4 Hz, 1H), 5.65 (dt, *J* = 14.8, 6.9 Hz, 1H), 4.33 (d, *J* = 8.5 Hz, 1H), 4.07 (q, *J* = 7.1 Hz, 2H), 3.07 (d, *J* = 7.1 Hz, 2H), 1.19 (t, *J* = 7.2 Hz, 3H). **^{13}C-NMR** (151 MHz, Acetonitrile-d_3): δ 174.0, 172.1, 140.0, 132.49, 129.6 (x2), 128.8 (x2), 128.1, 126.1, 61.2, 55.0, 38.1, 14.4. **HRMS (ESI-MS)** calculated for $C_{14}H_{17}O_4^+$ $[M+H]^+$ 249.1121, found 249.1148.

(*E*)-2-(2-ethoxy-2-oxoethyl)-4-phenylbut-3-enoic acid

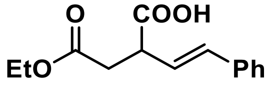

2kβ

2kβ was synthesized according to the electrosynthesis **Procedure E** (in this case E_{WE}= -1.9V vs Ag/AgCl was used to better ensure the dianion formation) as a white solid. NMRy: 12% **^{1}H-NMR** (400 MHz, Acetonitrile-d_3): δ 7.40 (dd, *J* = 8.2, 1.5 Hz, 2H), 7.36 – 7.29 (m, 2H), 7.29 – 7.23 (m, 1H), 6.58 (d, *J* = 16.0 Hz, 1H), 6.26 (dd, *J* = 16.0, 8.2 Hz, 1H), 4.10 (q, *J* = 7.1 Hz, 2H), 3.64 – 3.56 (m, 1H), 2.83 (dd, *J* = 16.7, 8.8 Hz, 1H), 2.60 (dd, *J* = 16.6, 5.7 Hz, 1H), 1.20 (t, *J* = 7.1 Hz, 3H). **^{13}C-NMR** (75 MHz, Acetonitrile-d_3): δ 174.2, 172.2, 137.6, 133.4, 129.6 (x2), 128.7, 127.1 (x2), 127.0, 61.3, 45.4, 36.8, 14.4. **HRMS (ESI-MS)** calculated for $C_{14}H_{17}O_4^+$ $[M+H]^+$ 249.1121, found 249.1148.

(*E*)-6-(dimethylamino)-6-oxo-2-phenylhex-3-enoic acid

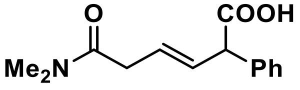

2lδ

2lδ was synthesized according to the electrosynthesis **Procedure E** as a white solid. NMRy: 32% **^{1}H-NMR** (600 MHz, Acetonitrile-d_3): δ 7.30 (td, *J* = 11.9, 11.0, 7.0 Hz, 5H), 5.89 (dd, *J* = 15.5, 8.5 Hz, 1H), 5.67 (dt, *J* = 15.1, 6.7 Hz, 1H), 4.31 (d, *J* = 8.5 Hz, 1H), 3.10 (d, *J* = 6.6 Hz, 2H), 2.94 (s, 3H), 2.83 (s, 3H). **^{13}C-NMR** (151 MHz, Acetonitrile-d_3): δ 174.6, 171.5, 140.4, 131.7, 129.5 (x2), 128.8 (x2), 127.9, 127.5, 55.5, 37.5, 37.5, 35.3. **HRMS (ESI-MS)** calculated for $C_{14}H_{18}NO_3^+$ $[M+H]^+$ 248.1281, found 249.1286.

(*E*)-6-oxo-2-phenyl-6-(phenylthio)hex-3-enoic acid

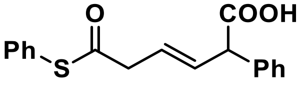

2mδ

2lδ was synthesized according to the electrosynthesis **Procedure E** as a white solid. NMRy: 24% **^{1}H-NMR** (400 MHz, Acetonitrile-d_3): δ 7.52 – 7.22 (m, 10H), 6.08 (dd, *J* = 15.5, 8.4 Hz, 1H), 5.71 (dt, *J* = 14.8, 7.1 Hz, 1H), 4.39 (d, *J* = 8.4 Hz, 1H), 3.43 (d, *J* = 7.1 Hz, 2H). **^{13}C-NMR** (151 MHz, Acetonitrile-d_3δ: 196.3, 173.6, 139.5, 135.6 (x2), 134.1, 130.4, 130.2 (x2), 129.7 (x2), 128.9 (x2), 128.7, 128.2, 125.2, 54.7, 47.2. **HRMS (ESI-MS)** calculated for $C_{18}H_{18}O_3Na^+$ $[M+Na]^+$ 335.0712, found 335.0703.

H. ELECTROCHEMICAL DIMER SYNTHESIS

Procedure F

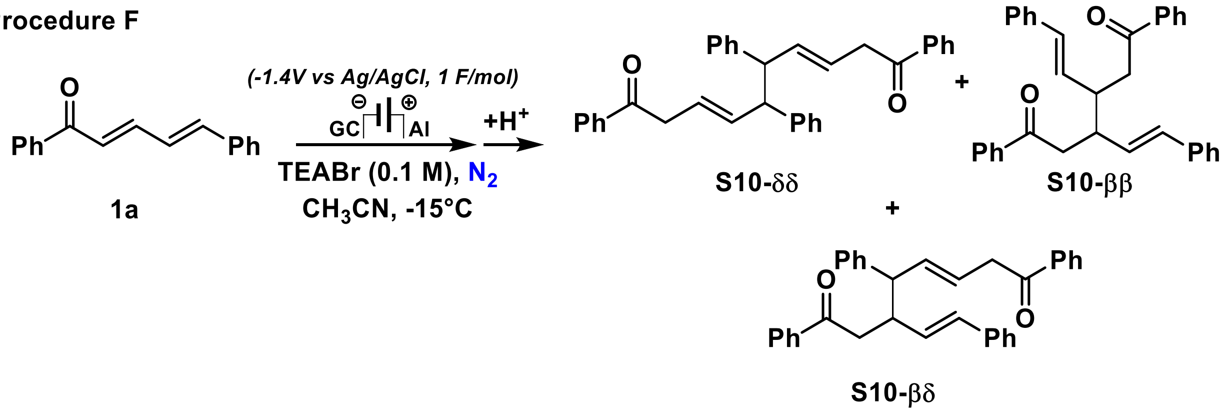

Scheme S3. Electrochemical dimerization of dienones and possible dimer structures **S10**.

Constant potential electrolysis experiment was performed in a custom-made 4-necked glass cell under stirring conditions of the solution with a magnetic stirring bar.

An oven-dried electrochemical cell was fully equipped with a glassy carbon rod working electrode (SIGRADUR, HTW, ca 1.5 cm^2 geometric area), an Ag/AgCl (NaCl 3 M) – separated from the bulk solution by means of a salt bridge (equipped with a Coralpor frit), filled with electrolyte solution – as reference electrode and an Al wire counter electrode (Sigma, 1.0 mm, 99.999%). Each electrode was accommodated in the cell by a PTFE O-ring and secured with a screw cap.

The substrate **1a** (0.3 mmol, 1 eq) and the supporting electrolyte tetraethylammonium bromide, TEABr, 1.5 mmol, 5 eq) were added to the electrochemical cell. Then the cell was filled with anhydrous CH_3CN. The cell was then placed in a NaCl-ice batch at the medium temperature of -15 °C. After saturating the electrolyte solution with N_2 for ca 30 min, the potential of -1.4 V vs Ag/AgCl was applied, and the N_2 atmosphere was kept above the solutions during the electrolysis experiments.

The experiment started with an initial current of ca – 3 mA and was stopped at Q= −25 C (0.86 F/mol), after ca. 3h.

At the end of the reaction, the electrolysis solution was evaporated under vacuum, acidified with 1 mL of HCl (1 M) and extracted with EtOAc. The organic phase was dried with $MgSO_4$ and then concentrated under vacuum. The crude extracted-mixture was analysed by mass spectrum, conducted in a Waters Acquity UPC^2 using CO_2/MeOH as mobile phase (**Figure S26**). Analysis of the mass information revealed the presence of a small amount of starting compound **1a** (min 2.7; $C_{17}H_{15}O^+$ $[M+H]^+$: 235 m/z) and of dimers **S10** ($C_{34}H_{31}O_2^+$ $[M+H]^+$: 471 m/z and $C_{34}H_{30}O_2Na^+$ $[M+Na]^+$: 493 m/z).

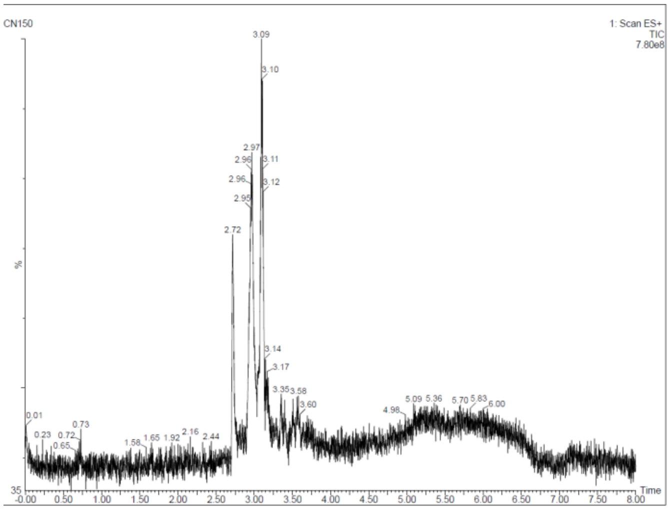

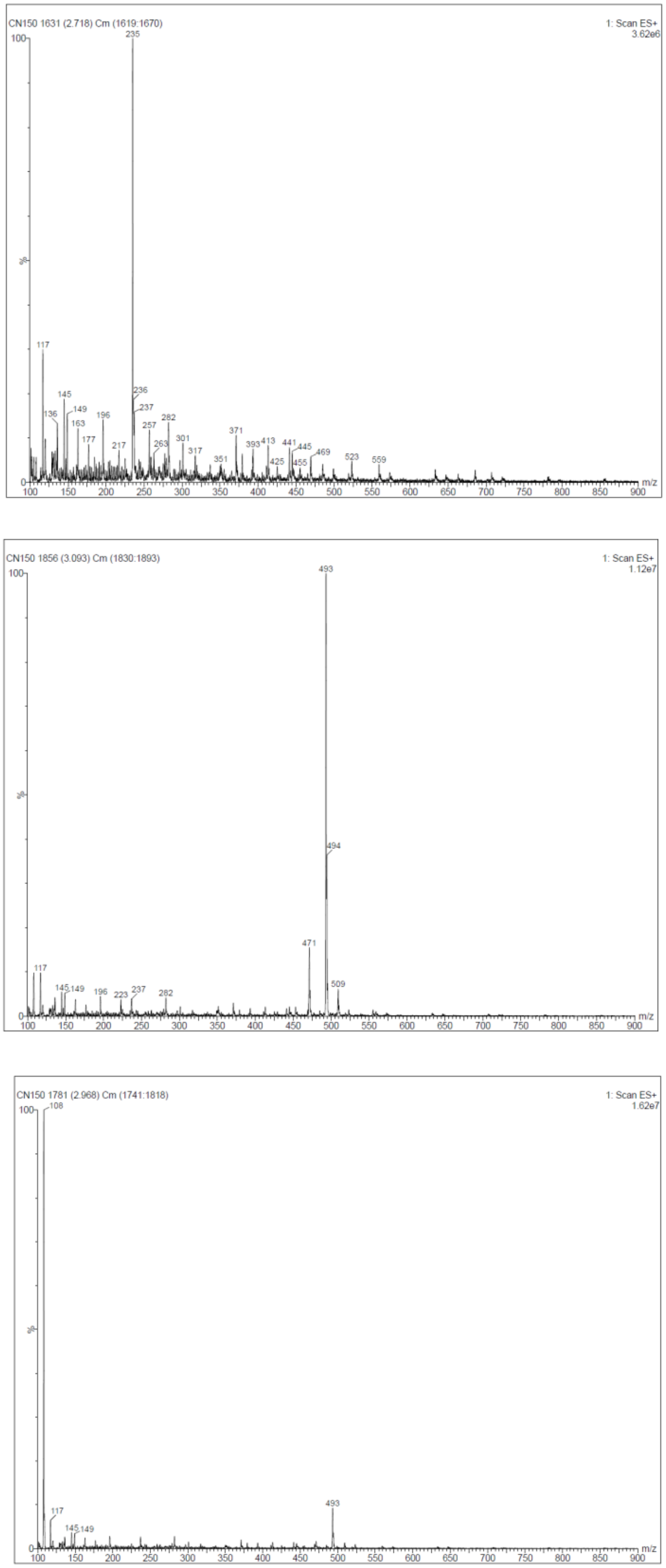

Figure S26. TIC and mass spectrum of the reaction crude electrosynthesis of dimers of compound **1a**.

I. CV ANALYSIS AND DIGITAL SIMULATION

I.1 CV ANALYSIS

The CV of model compound **1a** under argon at T = -15 °C (**Figure S1**) shows a shift to more negative potential of the first cathodic peak (E_{pc1}) increasing the scan rate.

The dependence of the cathodic peak potential (E_{pc1}) versus the logarithm of the scan rate in CV reveals a linear shift with a slope of -0.018 V; this is close to the theoretical value of -0.017 V expected for an electron transfer followed by a second order reaction of the reduced species, according to equation:

$$E_{\mathrm{pc},1} = E_{1/2} - 0.902\frac{RT}{nF} + \frac{RT}{3nF}\ln\left(\frac{2RTkC^*}{3nFv}\right)$$

The fitting provides a second order rate constant k = 1.17×10^5 $M^{-1}s^{-1}$ for **1a**$^{\bullet-}$ dimerization that occurs after first reduction of **1a**.

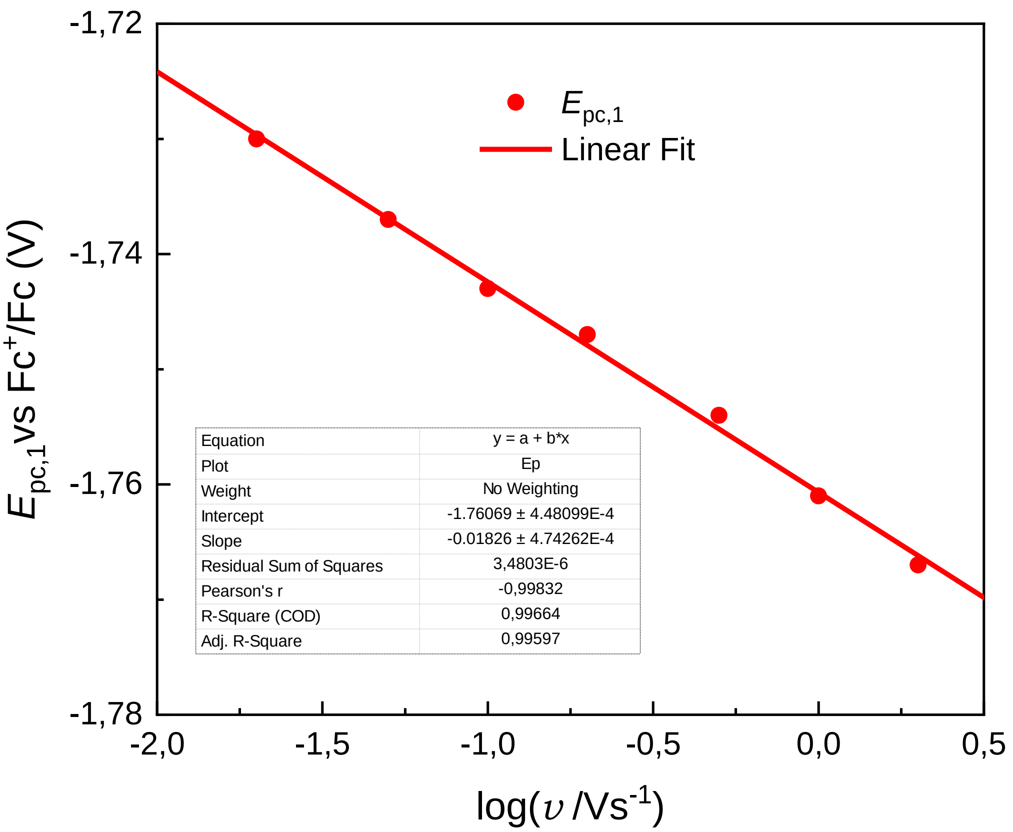

Figure S27. Linear fitting of the cathodic peak potential vs logarithm of the scan rate for CVs of **1a** under Ar.

I.1 Digital simulation of CV

The digital simulation of CV was performed using the software Digielch 3.0. First, the CV simulation of the voltammetric response of **1a** under an Ar atmosphere was conducted, considering the electron transfer and chemical reactions outlined in the following tables. The simulation was carried out by fitting the thermodynamic and kinetic parameters (highlighted in blue italics in the tables) to experimental CVs recorded at ca. 8 different scan rates, ranging from 0.1 to 20 V s^{-1} (examples of the experimental and simulated curves are presented in **Figure 1a** in the main text). The numbering of steps 1-5 corresponds to the one reported in the main text (Figure 1 and Figure 3). Additional steps are numbered 6-10.

Table S2. Electron transfer reactions for CV fitting under Ar.

	E^0	k^0	Step
**1a** + e^- ⇌ **1a**$^{\bullet-}$	*-1.74 V vs Fc^+/Fc*	*0.5*	1
**1a**$^{\bullet-}$ + e^- ⇌ **1a**$^{2-}$	*-2.30 V vs Fc^+/Fc*	*0.1*	6

Table S3. Chemical reactions for CV fitting under Ar.

	K	k	Step
**1a**$^{\bullet-}$ + **1a**$^{\bullet-}$ ⇌ dimers	Irrev.	*1.3×10^5 M^{-1} s^{-1}*	2
**1a**$^{2-}$ ⇌ **1a(H)**$^-$	Irrev.	10^5 s^{-1}	7

The protonation rate (**step 7)** was arbitrarily set to **10^5 s^{-1}**, as variations in this value had no significant effect on the simulation, except for altering the **E_0** value of **step 6**.

Additionally, the diffusion coefficient of **1a** was fitted to 1.1 × 10^{-5} cm^2 s^{-1}.

Next, CV measurements of **1a** at approximately 8 different scan rates were recorded in the presence of CO_2. In this case, all parameters identified in **Table S2** and **Table S3** remained constant. Alongside these parameters, additional reactions were considered, as outlined below (refer to **Scheme 3** in the main text for corresponding structures).

Table S4. Additional electron transfer reactions for CV fitting under CO_2.

	E^0	k^0	Step
**3a**$^{\bullet-}$ + e^- ⇌ **3a**$^{2-}$	*-1.68 V vs Fc^+/Fc*	*0.04*	4

Table S5. Additional chemical reactions for CV fitting under CO_2.

	K	k	Step
**$1a^{\bullet-} + CO_2 \rightleftharpoons 3a^{\bullet-}$**	*0.18*	*5×10^4 M^{-1} s^{-1}*	3
**$3a^{2-} + CO_2 \rightleftharpoons 4a^-$**	Irrev.	3×10^2 M^{-1} s^{-1}	5
**$1a^{2-} + CO_2 \rightleftharpoons 5a^-$**	Irrev.	10^7 M^{-1} s^{-1}	8
**$3a^{\bullet-} + 1a^{\bullet-} \rightleftharpoons 1a + 3a^{2-}$**	13.8	10^8 M^{-1} s^{-1}	9
**$3a^{\bullet-} + 3a^{\bullet-} \rightleftharpoons$ dimers**	Irrev.	*3.6×10^7 M^{-1} s^{-1}*	10

Step 4 represents the reduction of **$3a^{\bullet-}$**, the activated adduct between the radical anion and CO_2. The experimental potential is close to the value obtained by DFT (–1.80 V vs Fc^+/Fc).

The rate constant $k = 5 \times 10^4$ M^{-1} s^{-1} in **step 3** had small impact on the simulation. From this rate constant, a $\Delta G^\ddagger = 8.1$ kcal mol^{-1} was calculated from the Arrhenius equation, $k = Z \exp(-\Delta G^\ddagger/RT)$, assuming a pre-exponential factor $Z = 5\times10^{11}$ s^{-1}. This value is close to $\Delta G^\ddagger = 10.0$ kcal mol^{-1} obtained from DFT calculations.

The equilibrium constant K = 0.18 for step 3 was determined with high accuracy due to its significant impact on simulations. This value aligns with literature data for CO_2 adducts with oxygen-bearing radical anions. Reported constants for CO_2 adduct formation with benzophenone and acetophenone radical anions range from 0.5 to 20.[23] Since these radical anions have a less delocalized negative charge than dienone **$1a^{\bullet-}$**, a larger equilibrium constant was expected.

A lower limit for the rate constants for **step 5** was determined as $k > 10^3$ M^{-1} s^{-1}, corresponding to $\Delta G^\ddagger < 10.1$ kcal mol^{-1}. Within experimental and computation error, this value is compatible to the $\Delta G^\ddagger$ value determined by DFT ($\Delta G^\ddagger < 10.7$ kcal mol^{-1}).

$k = 4\times10^6$ M^{-1} s^{-1} for **step 8** were calculated using the activation free energies $\Delta G^\ddagger = 5.9$ kcal mol^{-1} obtained from DFT calculations. Changing this value ± two orders of magnitudes had no effect on the simulations.

Step 9 represents the homogeneous electron transfer between the two reduced species **$3a^{\bullet-}$** + **$1a^{\bullet-}$**. This rate constant was automatically determined by the software, as it depends on the standard reduction potentials of the two species. A high rate constant of $k = 10^8$ M^{-1} s^{-1} was arbitrarily assigned to this reaction, as it represents a simple electron transfer with minimal driving force. Variation of this value of two orders of magnitude had no effect on the simulation.

Step 10 describes the coupling of two **$3a^{\bullet-}$** adducts. The simulation software determined a high rate constant of $k = 3.6 \times 10^7$ M^{-1} s^{-1}, approximately two orders of magnitude higher than the coupling rate for **$1a^{\bullet-}$**. This increase in coupling rate was expected, as the negative charge is significantly more shielded in the **$3a^{\bullet-}$** adducts compared to the simpler radical anion **$1a^{\bullet-}$**. A similar trend was observed for the electrocarboxylative dimerization of olefins.[24]

The onset of a small reduction wave is observed at -2.3 V vs SCE. This might be ascribed to the direct reduction of residual **1a**$^{\bullet-}$.

J. DFT CALCULATIONS

J.1 REACTION PROFILE FOR 1A$^{\bullet-}$ CARBOXYLATION

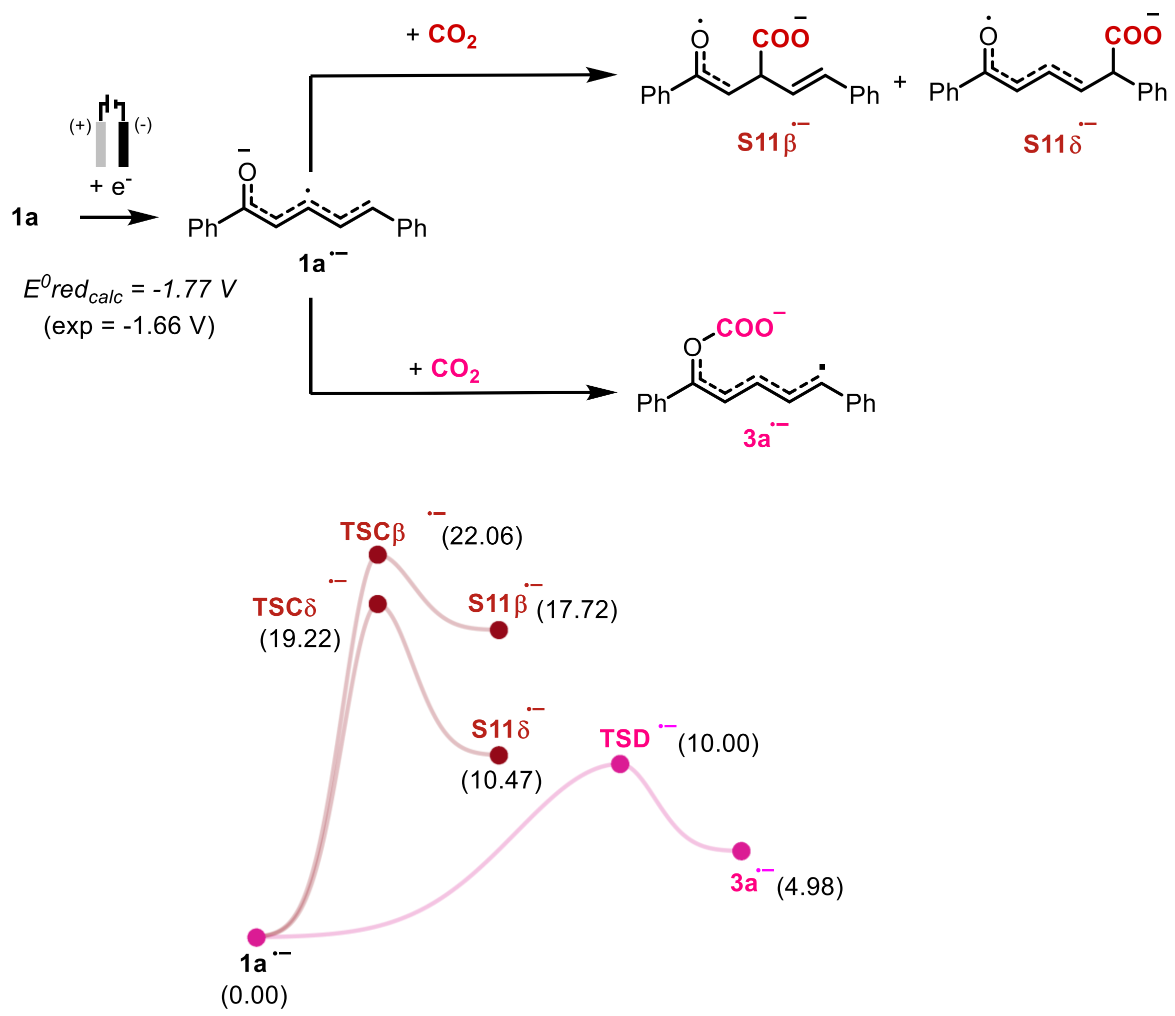

Scheme S4. – Possible carboxylation pathways for **1a**$^{\bullet-}$ occurring at C-sites (dark red lines), leading to the formation of **S11β**$^{\bullet-}$ and **S11δ**$^{\bullet-}$, and occurring at O-site (violet line), leading to formation of intermediate **3a**$^{\bullet-}$. Energies are expressed in kcalmol^{-1}.

J.2 OPTIMIZED GEOMETRIES OF RADICAL ANIONS

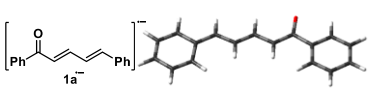

```
-1 2
C 3.70808200 1.21358500 -0.35011600
C 3.62836900 -0.14601300 -0.03608300
C 4.80648400 -0.79796000 0.33377400
C 6.01268800 -0.11919900 0.40809900
C 6.07365500 1.23320100 0.10025900
C 4.91425900 1.89414200 -0.28369400
H 2.82436200 1.74928400 -0.67175300
H 4.75530900 -1.85424600 0.56323100
H 6.91031900 -0.64769800 0.70632900
H 7.01512800 1.76571700 0.15250400
H 4.95006900 2.94606300 -0.54025300
O 2.45511200 -2.20207500 -0.17731300
C 2.35430700 -0.94612900 -0.09914900
C 1.12503100 -0.25710400 -0.03868000
H 1.13645900 0.82116400 0.07281500
C -0.11693400 -0.89089500 -0.08737600
H -0.12034400 -1.97311900 -0.19275400
C -1.35347700 -0.23832000 -0.01539300
H -1.32048900 0.84478700 0.08783300
C -2.58089100 -0.85863100 -0.06766100
H -2.59204500 -1.94127000 -0.16926900
C -3.87646500 -0.22494400 -0.00453300
C -4.06248300 1.16558800 0.12148200
C -5.03860600 -1.01657700 -0.07166100
C -5.32872000 1.71947600 0.17570500
H -3.20325600 1.82207900 0.17697000
C -6.30336100 -0.45881300 -0.01707200
H -4.93274500 -2.09154600 -0.16897500
C -6.46266100 0.91661000 0.10729700
H -5.43439900 2.79370100 0.27255100
H -7.17372000 -1.10203100 -0.07192100
H -7.45119300 1.35561800 0.15000000
```

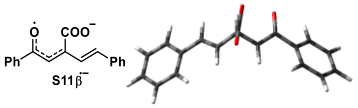

```
-1 2
C 3.77025600 -0.36976600 1.13051000
C 3.41247300 -0.48115800 -0.21135400
C 4.22193100 -1.22586800 -1.06674400
C 5.35654500 -1.85951600 -0.58903600
C 5.70626300 -1.74286200 0.75002300
C 4.91441000 -0.99226800 1.60657600
H 3.17193800 0.22488400 1.80884800
H 3.94768800 -1.30480200 -2.11064000
H 5.97135700 -2.44453400 -1.26127200
H 6.59607400 -2.23352600 1.12380000
H 5.18854900 -0.88792100 2.64858800
O 2.17653900 0.49872900 -1.97887400
C 2.20162600 0.19789300 -0.77901900
C 1.08897600 0.45208800 0.08369800
H 1.13064200 0.12162600 1.11371600
C -1.37229300 0.56954400 0.14921500
H -1.44111200 0.45810600 1.22630300
C -2.36976700 0.19055300 -0.64532100
H -2.24987100 0.31883600 -1.71834200
C -3.64920000 -0.40703500 -0.23212700
C -4.02069000 -0.57629100 1.10495000
C -4.54293200 -0.82978700 -1.21762200
C -5.23489000 -1.15153700 1.43767100
H -3.35858000 -0.25235600 1.89795300
C -5.76030800 -1.40608000 -0.88627800
H -4.27603000 -0.70464000 -2.26084700
C -6.11195700 -1.57093400 0.44448000
H -5.50140200 -1.27189200 2.48045400
H -6.43456900 -1.72601500 -1.67099200
H -7.06121700 -2.01956700 0.70841200
C -0.10411600 1.18785800 -0.34903000
H -0.11222700 1.28765600 -1.43214200
C 0.04912600 2.67162100 0.26004700
O -0.13199200 2.76760300 1.48391000
O 0.34245700 3.54559300 -0.56724500
```

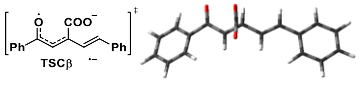

```
-1 2
C -3.79477700 -0.89501800 0.89442300
C -3.61526700 -0.24868500 -0.32836100
C -4.65983000 -0.27307200 -1.25154100
C -5.84168000 -0.93999900 -0.97229700
C -6.00704100 -1.58386300 0.24701300
C -4.98137100 -1.55367800 1.18112800
H -3.01350200 -0.86737200 1.64333000
H -4.52842500 0.23917600 -2.19589200
H -6.63770100 -0.95728100 -1.70658400
H -6.93174100 -2.10136700 0.46977000
H -5.10655200 -2.04061500 2.14030600
O -2.43382500 1.43461500 -1.50588100
C -2.35761800 0.49861100 -0.68117900
C -1.14086700 0.08273100 -0.08486400
H -1.15682900 -0.75400900 0.60238600
C 1.35300000 0.07231000 -0.03392400
H 1.32532300 -0.70186900 0.72816100
C 2.51136200 0.37391000 -0.63677600
H 2.50234100 1.14080700 -1.40746300
C 3.81833800 -0.23643300 -0.37882800
C 4.05421800 -1.13623200 0.66733600
C 4.89421700 0.08962200 -1.21001900
C 5.30758100 -1.68996200 0.86234000
H 3.25268300 -1.40295200 1.34458300
C 6.15045700 -0.46336300 -1.01468600
H 4.73767600 0.78848500 -2.02394000
C 6.36432100 -1.35905900 0.02244400
H 5.46362900 -2.38289200 1.68008500
H 6.96471300 -0.19251600 -1.67550400
H 7.34383300 -1.79254100 0.17926800
C 0.08742100 0.73907500 -0.30215500
H 0.09836000 1.46033900 -1.11207200
C 0.10055000 2.05727100 1.23630400
O 0.08288400 3.14422500 0.73255900
O 0.12945600 1.46611100 2.27774900
```

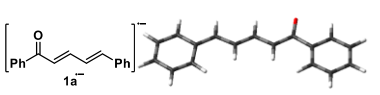


```
-1 2
C 3.70808200 1.21358500 -0.35011600
C 3.62836900 -0.14601300 -0.03608300
C 4.80648400 -0.79796000 0.33377400
C 6.01268800 -0.11919900 0.40809900
C 6.07365500 1.23320100 0.10025900
C 4.91425900 1.89414200 -0.28369400
H 2.82436200 1.74928400 -0.67175300
H 4.75530900 -1.85424600 0.56323100
H 6.91031900 -0.64769800 0.70632900
H 7.01512800 1.76571700 0.15250400
H 4.95006900 2.94606300 -0.54025300
O 2.45511200 -2.20207500 -0.17731300
C 2.35430700 -0.94612900 -0.09914900
C 1.12503100 -0.25710400 -0.03868000
H 1.13645900 0.82116400 0.07281500
C -0.11693400 -0.89089500 -0.08737600
H -0.12034400 -1.97311900 -0.19275400
C -1.35347700 -0.23832000 -0.01539300
H -1.32048900 0.84478700 0.08783300
C -2.58089100 -0.85863100 -0.06766100
H -2.59204500 -1.94127000 -0.16926900
C -3.87646500 -0.22494400 -0.00453300
C -4.06248300 1.16558800 0.12148200
C -5.03860600 -1.01657700 -0.07166100
C -5.32872000 1.71947600 0.17570500
H -3.20325600 1.82207900 0.17697000
C -6.30336100 -0.45881300 -0.01707200
H -4.93274500 -2.09154600 -0.16897500
C -6.46266100 0.91661000 0.10729700
H -5.43439900 2.79370100 0.27255100
H -7.17372000 -1.10203100 -0.07192100
H -7.45119300 1.35561800 0.15000000
```


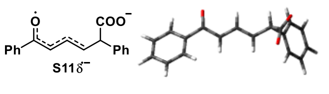

```
-1 2
C -4.96117400 -0.80072800 -0.86908600
C -3.93757100 -0.13464300 -0.19732400
C -4.18739100 0.38280200 1.07213500
C -5.43648800 0.23682900 1.65652400
C -6.44427000 -0.43943800 0.98507900
C -6.20353000 -0.95999800 -0.27998500
H -4.76693900 -1.19549900 -1.85771500
H -3.41775100 0.92469200 1.60594000
H -5.62216400 0.65376800 2.63802600
H -7.41694700 -0.55909600 1.44526900
H -6.98719200 -1.48931000 -0.80693700
O -2.55728000 -0.04691200 -2.11364100
C -2.61496400 0.02019100 -0.88617300
C -1.43064100 0.22042600 -0.07822100
H -1.51571800 0.19171100 1.00025600
C -0.17243800 0.41490800 -0.64547200
H -0.10793500 0.43659800 -1.72914000
C 0.96653600 0.57737400 0.09214300
H 0.89848700 0.56150800 1.17512000
C 2.92895800 2.17258700 -0.05016600
O 2.61707600 2.59289500 1.08121700
O 3.69306400 2.69973200 -0.87984900
C 3.26744200 -0.32056100 -0.09125500
C 3.84174100 -1.13366600 -1.06279500
C 3.58676000 -0.55279300 1.24574700
C 4.71778400 -2.15234700 -0.71208200
H 3.60117900 -0.96759600 -2.10644300
C 4.45902700 -1.57044100 1.59855900
H 3.15782500 0.08179900 2.01145100
C 5.02880400 -2.37545900 0.62055400
H 5.15624600 -2.77290400 -1.48391400
H 4.69740400 -1.73497200 2.64225800
H 5.71027500 -3.17020100 0.89676900
C 2.31572900 0.79307800 -0.49058700
H 2.24446600 0.80506600 -1.57832400
```

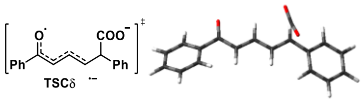

```
-1 2
C -5.15527900 0.29185900 -0.95733500
C -4.01855700 -0.01371800 -0.21019800
C -4.14192600 -0.91115800 0.84998000
C -5.36619600 -1.49028600 1.14964300
C -6.48655500 -1.18866800 0.38861100
C -6.37666600 -0.29425000 -0.66792800
H -5.06459200 0.99772400 -1.77253600
H -3.28381800 -1.14789800 1.46556800
H -5.44538500 -2.17677400 1.98339900
H -7.44107600 -1.64441300 0.62032200
H -7.24639000 -0.05238700 -1.26629800
O -2.76861900 1.74352100 -1.18126900
C -2.72007100 0.66106900 -0.56824600
C -1.50168500 0.01438900 -0.20842400
H -1.54687700 -0.95778800 0.26716400
C -0.25489400 0.56339500 -0.47249700
H -0.22942900 1.54006700 -0.94821600
C 0.95620400 -0.04439200 -0.17301900
H 0.91328000 -1.02414200 0.29594100
C 2.31112100 1.60083500 1.43955100
O 2.76243800 0.86911100 2.26298300
O 1.88058700 2.68229500 1.18250100
C 3.47134800 -0.21147700 -0.41755700
C 4.57770500 0.33262600 -1.08005200
C 3.63760300 -1.45237000 0.20694800
C 5.79191100 -0.33244500 -1.12547400
H 4.47656900 1.29537600 -1.56882600
C 4.85189200 -2.11833300 0.16206000
H 2.81332500 -1.90267200 0.74478700
C 5.93784700 -1.56569200 -0.50411000
H 6.62824800 0.11399700 -1.64974500
H 4.95166100 -3.07832600 0.65431400
H 6.88494000 -2.08911400 -0.53785400
C 2.21562900 0.54688900 -0.37890500
H 2.21769300 1.43729400 -1.00003500
```

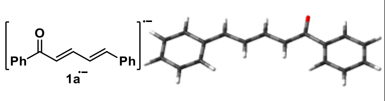


```
-1 2
C 3.70808200 1.21358500 -0.35011600
C 3.62836900 -0.14601300 -0.03608300
C 4.80648400 -0.79796000 0.33377400
C 6.01268800 -0.11919900 0.40809900
C 6.07365500 1.23320100 0.10025900
C 4.91425900 1.89414200 -0.28369400
H 2.82436200 1.74928400 -0.67175300
H 4.75530900 -1.85424600 0.56323100
H 6.91031900 -0.64769800 0.70632900
H 7.01512800 1.76571700 0.15250400
```


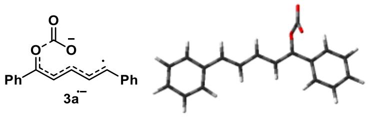

```
-1 2
C 3.17950400 -1.91237100 0.38350900
C 3.15569300 -0.62984400 -0.17868400
C 4.35825800 -0.10118900 -0.66088100
C 5.53366000 -0.83079600 -0.59687900
C 5.53982900 -2.10448600 -0.04602800
C 4.35524900 -2.63890800 0.44551800
H 2.27678200 -2.34182300 0.79753800
H 4.36241800 0.88946900 -1.09547500
H 6.45087900 -0.40158000 -0.98065600
H 6.45905000 -2.67382200 0.00658200
```

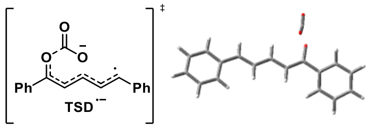

```
-1 2
C 3.29640100 -2.03744600 -0.19304600
C 3.29056500 -0.63774000 -0.22182600
C 4.51088100 0.02201400 -0.04642200
C 5.68408600 -0.68287000 0.17019700
C 5.67094000 -2.07047500 0.20637200
C 4.46936100 -2.74259000 0.01919800
H 2.37952300 -2.58858100 -0.35709100
H 4.52003600 1.10320300 -0.08158400
H 6.61461300 -0.14625900 0.31127000
H 6.58698600 -2.62361900 0.37159200
```

```
H 4.95006900 2.94606300 -0.54025300
O 2.45511200 -2.20207500 -0.17731300
C 2.35430700 -0.94612900 -0.09914900
C 1.12503100 -0.25710400 -0.03868000
H 1.13645900 0.82116400 0.07281500
C -0.11693400 -0.89089500 -0.08737600
H -0.12034400 -1.97311900 -0.19275400
C -1.35347700 -0.23832000 -0.01539300
H -1.32048900 0.84478700 0.08783300
C -2.58089100 -0.85863100 -0.06766100
H -2.59204500 -1.94127000 -0.16926900
C -3.87646500 -0.22494400 -0.00453300
C -4.06248300 1.16558800 0.12148200
C -5.03860600 -1.01657700 -0.07166100
C -5.32872000 1.71947600 0.17570500
H -3.20325600 1.82207900 0.17697000
C -6.30336100 -0.45881300 -0.01707200
H -4.93274500 -2.09154600 -0.16897500
C -6.46266100 0.91661000 0.10729700
H -5.43439900 2.79370100 0.27255100
H -7.17372000 -1.10203100 -0.07192100
H -7.45119300 1.35561800 0.15000000
```

```
H 4.34956900 -3.62633400 0.89005200
C 1.92531400 0.15962300 -0.26965100
C 0.66116900 -0.36298800 -0.16190200
H 0.56300800 -1.43376400 -0.02662200
C -0.51471800 0.39126500 -0.23317800
H -0.43013600 1.46341900 -0.37568700
C -1.79857800 -0.16722000 -0.12792500
H -1.85479000 -1.24293800 0.01537100
C -2.95167100 0.56280400 -0.19529900
H -2.85899800 1.63480300 -0.34697500
C -4.30937600 0.06060400 -0.08790300
C -4.61819300 -1.28859900 0.14307200
C -5.37737800 0.95958800 -0.21747900
C -5.93153300 -1.71145100 0.23639100
H -3.82460300 -2.01625800 0.25420300
C -6.69167800 0.53439300 -0.12427500
H -5.16389100 2.00742000 -0.39493300
C -6.97736200 -0.80482500 0.10323700
H -6.14377400 -2.75813900 0.41613500
H -7.49650000 1.25132100 -0.22921000
H -8.00375400 -1.14062900 0.17749700
O 2.06317100 1.47439600 -0.58536200
C 2.50983800 2.39069600 0.43814700
O 2.58222900 1.93373500 1.57952400
O 2.73292300 3.51075500 -0.01998100
```

```
H 4.44716700 -3.82553900 0.03128500
C 2.06400000 0.17277900 -0.43840100
C 0.80178300 -0.38507800 -0.23557100
H 0.73595300 -1.41136900 0.10731700
C -0.39184200 0.30887200 -0.42221800
H -0.33405600 1.33496500 -0.77375700
C -1.66617600 -0.23404200 -0.18404600
H -1.69604100 -1.26299000 0.16724000
C -2.84192300 0.44161700 -0.36280000
H -2.78298200 1.46916300 -0.71258900
C -4.18163100 -0.06356200 -0.13179000
C -4.45416800 -1.36627300 0.31800500
C -5.27961900 0.77980000 -0.36331600
C -5.75334200 -1.79437400 0.52337400
H -3.64062500 -2.05420600 0.51012300
C -6.57910700 0.34933200 -0.15718600
H -5.09962400 1.79085900 -0.71108200
C -6.82691400 -0.94237100 0.28845900
H -5.93238300 -2.80468300 0.87099300
H -7.40382500 1.02599500 -0.34528400
H -7.84178800 -1.28217200 0.45084900
O 2.22773800 1.40977200 -0.78811400
C 1.88153600 2.81490500 0.49829900
O 1.72038500 2.26909300 1.53198600
O 1.96163500 3.78116600 -0.17424300
```

J.3 Optimized geometries of dianions

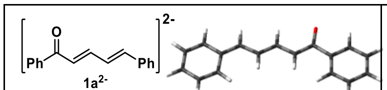

$1a^{2-}$

```
-2 1
C 3.66836800 1.24111900 -0.30120900
C 3.61892200 -0.13530200 -0.03975900
C 4.82799300 -0.76168100 0.27923300
C 6.01936500 -0.05423200 0.35913600
C 6.04369700 1.31028100 0.10972600
C 4.85548800 1.95101700 -0.22565400
H 2.76716300 1.76833500 -0.58711800
H 4.80637800 -1.82763200 0.46336700
H 6.93580800 -0.57343600 0.61634200
H 6.97170200 1.86594100 0.16550000
H 4.85623100 3.01373800 -0.43940100
O 2.53829100 -2.25516100 -0.14198900
C 2.36825400 -0.97668400 -0.09926900
C 1.14787200 -0.33658900 -0.06584000
H 1.12692100 0.74607800 0.00651600
C -0.13526900 -1.00024700 -0.08977700
H -0.11659800 -2.08710200 -0.15699900
C -1.34611900 -0.37481800 -0.03672900
H -1.30766600 0.71445500 0.02926400
C -2.64381700 -0.96270500 -0.05538100
H -2.69559400 -2.04881400 -0.11843800
C -3.86899300 -0.27819300 -0.00223200
C -4.01021000 1.14673500 0.08036400
C -5.11451500 -0.98898100 -0.02874600
C -5.24499400 1.76294200 0.12957600
H -3.12354900 1.76941900 0.10566100
C -6.33605000 -0.35542100 0.02098500
H -5.08307500 -2.07310200 -0.09101900
C -6.43725600 1.03806700 0.10124100
H -5.28167200 2.84685100 0.19177200
H -7.23946000 -0.95780400 -0.00338200
H -7.39932300 1.53249600 0.13984700
```

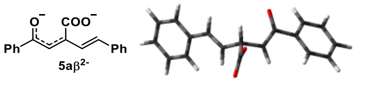

$5a\beta^{2-}$

```
-2 1
C 3.93540800 -0.02746100 0.75042700
C 3.11528900 -0.59992700 -0.22425200
C 3.47084400 -1.85825100 -0.70961200
C 4.58099300 -2.53407100 -0.22358100
C 5.37833800 -1.95595700 0.75458400
C 5.05121700 -0.69451600 1.23481900
H 3.70833300 0.96329500 1.12391800
H 2.85464400 -2.29860900 -1.48304500
H 4.82660700 -3.51598500 -0.61125800
H 6.24961900 -2.47705700 1.13208300
H 5.67414000 -0.22266700 1.98569200
O 1.53890800 -0.26317900 -1.97489800
C 1.87906100 0.07626300 -0.78646700
C 1.21815100 0.95983600 0.02703000
H 1.55829500 1.12633200 1.04171100
C -1.24280200 0.93297400 0.13138800
H -1.31689000 0.89852800 1.21439800
C -2.15685300 0.32708100 -0.62742900
H -2.05257100 0.39464100 -1.70819700
C -3.32229000 -0.44258200 -0.16622800
C -3.59669700 -0.68960500 1.18329300
C -4.21221000 -0.95732500 -1.11235700
C -4.71340100 -1.41370200 1.56429200
H -2.92861900 -0.31369800 1.94790500
C -5.33135500 -1.68392600 -0.73318500
H -4.01997500 -0.78211000 -2.16498900
C -5.58960400 -1.91576700 0.60926700
H -4.90200100 -1.58981600 2.61639700
H -6.00280500 -2.06978400 -1.49056400
H -6.46163000 -2.48229600 0.91054300
C -0.04816500 1.66888800 -0.39300400
H -0.08856100 1.67340000 -1.48295300
C -0.01511100 3.13660900 0.09822100
O -0.33407400 3.35234400 1.29220500
O 0.35723200 4.00139300 -0.72916400
```

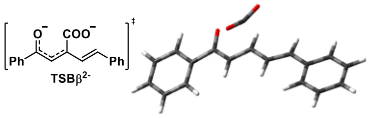

$TSB\beta^{2-}$

```
-2 1
C -3.75728600 -1.60952000 -0.13105400
C -3.68912100 -0.22313300 -0.32019300
C -4.88069100 0.49580300 -0.18730100
C -6.07540600 -0.12755800 0.14377600
C -6.11897700 -1.50038500 0.34180400
C -4.94839100 -2.23699200 0.19733500
H -2.86767800 -2.21292500 -0.26160200
H -4.84182300 1.56486100 -0.35096100
H -6.97907600 0.46261100 0.24671000
H -7.05024500 -1.99222200 0.59466400
H -4.96653400 -3.31243400 0.33189300
O -2.58274300 1.70896400 -1.16584500
C -2.42976900 0.52856400 -0.66902900
C -1.21740700 -0.06471900 -0.40025600
H -1.20244800 -1.03465600 0.08563000
C 1.28398900 -0.04015400 -0.37947200
H 1.22733000 -0.92094200 0.26321200
C 2.55254800 0.33499600 -0.82355000
H 2.61577700 1.16246100 -1.52688700
C 3.79192900 -0.23667600 -0.41491100
C 3.92379200 -1.28015600 0.54101800
C 5.01486500 0.22859600 -0.96937800
C 5.15491300 -1.80812800 0.88383500
H 3.03926000 -1.67804300 1.02393500
C 6.23926700 -0.30255300 -0.61611600
H 4.97611900 1.03028100 -1.70048000
C 6.33540300 -1.33555500 0.31551100
H 5.19616900 -2.60610000 1.61829300
H 7.13895800 0.09415000 -1.07516200
H 7.29529700 -1.75240600 0.59216700
C 0.06237700 0.55930900 -0.65509200
H 0.05790100 1.42297200 -1.31489000
C 0.41923000 2.05394500 1.21558600
O 1.07478400 2.89416800 0.71575700
O -0.19286500 1.54165400 2.07988800
```

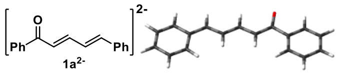

$1a^{2-}$

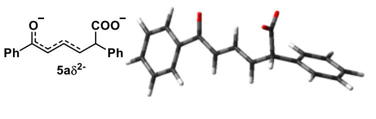

$5a\delta^{2-}$

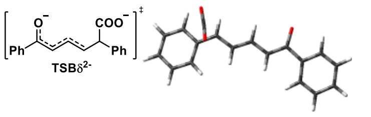

$TSB\delta^{2-}$

```
-2 1
C 3.66836800 1.24111900 -0.30120900
C 3.61892200 -0.13530200 -0.03975900
C 4.82799300 -0.76168100 0.27923300
C 6.01936500 -0.05423200 0.35913600
C 6.04369700 1.31028100 0.10972600
C 4.85548800 1.95101700 -0.22565400
H 2.76716300 1.76833500 -0.58711800
H 4.80637800 -1.82763200 0.46336700
H 6.93580800 -0.57343600 0.61634200
H 6.97170200 1.86594100 0.16550000
H 4.85623100 3.01373800 -0.43940100
O 2.53829100 -2.25516100 -0.14198900
C 2.36825400 -0.97668400 -0.09926900
C 1.14787200 -0.33658900 -0.06584000
H 1.12692100 0.74607800 0.00651600
C -0.13526900 -1.00024700 -0.08977700
H -0.11659800 -2.08710200 -0.15699900
C -1.34611900 -0.37481800 -0.03672900
H -1.30766600 0.71445500 0.02926400
C -2.64381700 -0.96270500 -0.05538100
H -2.69559400 -2.04881400 -0.11843800
C -3.86899300 -0.27819300 -0.00223200
C -4.01021000 1.14673500 0.08036400
C -5.11451500 -0.98898100 -0.02874600
C -5.24499400 1.76294200 0.12957600
H -3.12354900 1.76941900 0.10566100
C -6.33605000 -0.35542100 0.02098500
H -5.08307500 -2.07310200 -0.09101900
C -6.43725600 1.03806700 0.10124100
H -5.28167200 2.84685100 0.19177200
H -7.23946000 -0.95780400 -0.00338200
H -7.39932300 1.53249600 0.13984700
```

```
-2 1
C 4.81405100 0.14082400 1.05501000
C 3.86049800 -0.00014400 0.04734300
C 4.21382200 -0.73987000 -1.08266300
C 5.46477300 -1.32985800 -1.19186600
C 6.39515900 -1.19565500 -0.16990400
C 6.06290300 -0.45451100 0.95590700
H 4.55253500 0.73237600 1.92290100
H 3.50987200 -0.84360700 -1.89861500
H 5.71659900 -1.89232500 -2.08308200
H 7.37132700 -1.65719300 -0.25435300
H 6.78118200 -0.33781500 1.75892900
O 2.48142600 1.67320000 1.00901900
C 2.51143900 0.67092300 0.22820000
C 1.42754500 0.12111400 -0.43779300
H 1.57484900 -0.76087300 -1.05040000
C 0.09054800 0.64086400 -0.32783100
H -0.02095300 1.50514100 0.32356600
C -1.00657000 0.16068100 -0.93339600
H -0.90372300 -0.71356400 -1.57669800
C -3.39120600 -0.35108300 -0.31671300
C -3.03563500 -1.27763500 0.65999900
C -4.69597600 -0.38212600 -0.80397800
C -3.95520100 -2.19714200 1.14301000
H -2.02193800 -1.27124000 1.04133100
C -5.62163200 -1.29941300 -0.32599700
H -4.99120200 0.32643400 -1.57075700
C -5.25455200 -2.21183000 0.65315400
H -3.65650400 -2.90678200 1.90550300
H -6.63028100 -1.30356100 -0.72163600
H -5.97264500 -2.93074800 1.02795300
C -2.49246800 2.00436700 0.01174300
O -2.46194200 3.07272900 -0.64082900
O -2.56846200 1.89755700 1.25403800
C -2.41561200 0.69056300 -0.82595500
H -2.74861900 0.96546000 -1.83207800
```

```
-2 1
C 5.25724100 0.75718600 -0.21776700
C 4.09259700 -0.01469300 -0.22926300
C 4.20965200 -1.35093600 0.17269700
C 5.42334600 -1.87831100 0.58440000
C 6.56663100 -1.08740000 0.60550500
C 6.47400700 0.23574300 0.19836200
H 5.18053200 1.78461300 -0.54847100
H 3.34318800 -1.99935300 0.14774800
H 5.48001100 -2.91862500 0.88330100
H 7.51505300 -1.50103200 0.92562500
H 7.35594400 0.86617800 0.20249000
O 2.92556300 1.73091000 -1.34839900
C 2.80667700 0.63233800 -0.69035300
C 1.61534200 0.02833500 -0.34682600
H 1.64044200 -0.88222700 0.24254500
C 0.30700000 0.53693800 -0.68933200
H 0.28511500 1.46551300 -1.25893400
C -0.87735300 -0.03961100 -0.35784800
H -0.81258600 -0.96588400 0.21469400
C -3.39500200 -0.28658000 -0.49287000
C -3.49432400 -1.54288700 0.17654200
C -4.63096500 0.21617600 -1.00150300
C -4.70207200 -2.19692000 0.33488600
H -2.60157600 -2.00267700 0.58291800
C -5.82707100 -0.44789300 -0.83429700
H -4.61891800 1.16174000 -1.53455900
C -5.89362200 -1.66951100 -0.15882100
H -4.71476600 -3.14907800 0.85673800
H -6.73273700 -0.01042400 -1.24306300
H -6.83364500 -2.19129200 -0.03313100
C -2.70393500 1.79049200 1.43990200
O -3.20333600 2.71422100 0.92369600
O -2.27553300 1.08066500 2.26385300
C -2.19520200 0.44996600 -0.64172900
H -2.25730200 1.34159300 -1.26122100
```

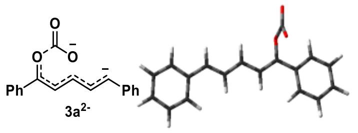

$3a^{2-}$

```
-2 1
C 3.08164700 -2.02751500 0.14886800
C 3.14812100 -0.65843200 -0.18173000
C 4.43482200 -0.12673500 -0.38702400
C 5.57271400 -0.91033400 -0.27781900
C 5.48281000 -2.25763700 0.04238100
C 4.22026600 -2.80360800 0.25504300
H 2.12260600 -2.49430900 0.33332100
H 4.53364100 0.91987600 -0.64153200
H 6.54381000 -0.45837900 -0.44564300
H 6.37208900 -2.86885600 0.12908000
H 4.12210200 -3.85199700 0.51292000
C 1.97527700 0.18707900 -0.30756300
C 0.67973200 -0.22375800 -0.20947000
H 0.51740700 -1.28277600 -0.02685200
C -0.48604500 0.56104800 -0.31574000
H -0.38066300 1.62734300 -0.49425000
C -1.76675900 0.02184600 -0.18645200
H -1.79498500 -1.05309600 -0.00228600
C -2.97531400 0.67980100 -0.25983700
H -2.96001200 1.75133200 -0.44748800
C -4.28009200 0.09122900 -0.10511600
C -4.51276000 -1.27724900 0.15885400
C -5.43272200 0.90051700 -0.21361900
C -5.79224300 -1.78425200 0.30036300
H -3.67457400 -1.95627300 0.25783900
C -6.70949600 0.38834600 -0.07084500
H -5.30511400 1.95907000 -0.41545600
C -6.91016000 -0.96351700 0.18848000
H -5.92012700 -2.84188300 0.50361000
H -7.56126100 1.05320700 -0.16299000
H -7.90853500 -1.36636400 0.30127100
O 2.21682800 1.51491700 -0.63944400
C 2.44303400 2.45532300 0.38329400
O 2.41288700 2.05840000 1.55565900
O 2.65236400 3.59545700 -0.06295900
```

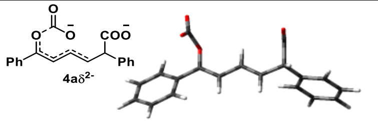

$4a\delta^{2-}$

```
-2 1
C 4.35653000 -0.60077600 0.97206200
C 3.38912000 -0.76570300 -0.01968600
C 3.66334200 -1.63771800 -1.07452100
C 4.85688400 -2.33891400 -1.12345200
C 5.80677100 -2.17653700 -0.12384500
C 5.55176100 -1.30111700 0.92185700
H 4.16439400 0.08045600 1.79071300
H 2.94648300 -1.75333200 -1.87742300
H 5.05178100 -3.00650100 -1.95359200
H 6.74155800 -2.72115000 -0.16535900
H 6.28737600 -1.16186500 1.70438800
C 2.10942900 -0.02909900 0.06713900
C 0.95894500 -0.47251400 -0.45763500
H 0.96232100 -1.43498600 -0.95623900
C -0.30654400 0.23278500 -0.39050500
H -0.30923700 1.19569100 0.10888000
C -1.43862000 -0.24909500 -0.90531500
H -1.41250800 -1.22610200 -1.38590500
C -3.85243300 -0.44308200 -0.26949200
C -3.58751300 -1.23620000 0.84365400
C -5.14977100 -0.43356900 -0.77694900
C -4.58902200 -1.99202300 1.43592000
H -2.58443400 -1.25800400 1.25153900
C -6.15585100 -1.18558200 -0.18797800
H -5.37393000 0.17360600 -1.64726800
C -5.87866500 -1.96949200 0.92333800
H -4.36076100 -2.60213500 2.30154300
H -7.15718600 -1.16259000 -0.60058500
H -6.66036300 -2.56087300 1.38366300
C -2.74152300 1.85702800 -0.31264900
O -2.66226000 2.78891100 -1.14231600
O -2.76955400 1.96131300 0.93041100
C -2.78708500 0.41711200 -0.91545200
H -3.06934700 0.53986600 -1.96524200
O 2.11587000 1.09805700 0.83852800
C 2.82816000 2.24620600 0.35695100
O 3.21366500 2.19758100 -0.81367800
O 2.92357300 3.12509600 1.21583400
```

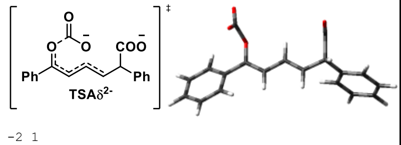

$TSA\delta^{2-}$

```
-2 1
C 4.80086200 -0.07333800 0.36770500
C 3.56065300 -0.65689700 0.07654200
C 3.55163100 -2.03176800 -0.20729500
C 4.71518700 -2.77917500 -0.18295300
C 5.93576800 -2.18412300 0.11627100
C 5.96633500 -0.82452800 0.38769100
H 4.84399300 0.98522300 0.58596700
H 2.62396800 -2.52706000 -0.46328400
H 4.67047200 -3.83805700 -0.40856600
H 6.84542200 -2.77095500 0.13048900
H 6.90734300 -0.33924800 0.61865900
C 2.34569400 0.16200400 0.07371900
C 1.08279200 -0.30878600 -0.00552900
H 0.95440300 -1.38539400 -0.06195900
C -0.11640300 0.46497000 -0.01995600
H -0.02823500 1.54546600 0.03948000
C -1.36217800 -0.10136900 -0.09809600
H -1.38326400 -1.18883800 -0.16906200
C -3.86474900 -0.02764500 -0.45652500
C -4.07859900 -1.41262300 -0.59740200
C -4.98264300 0.79985200 -0.68740900
C -5.31819200 -1.92492400 -0.94307400
H -3.26216500 -2.10340100 -0.42799500
C -6.21908900 0.28485300 -1.03150900
H -4.86231700 1.87391900 -0.59026800
C -6.40497700 -1.08760700 -1.16454600
H -5.43726600 -2.99836100 -1.04066600
H -7.04881800 0.96203600 -1.20082600
H -7.37140000 -1.49304700 -1.43588500
C -2.76662300 0.45329900 2.28350000
O -2.34104800 1.51167100 2.57397200
O -3.21921900 -0.61344000 2.48514000
C -2.60542400 0.56041100 -0.05895500
H -2.57563400 1.64590500 -0.08873500
O 2.54010000 1.51757900 0.26468100
C 2.82493500 2.33306600 -0.85646800
O 2.81904600 1.79351400 -1.96904000
O 3.04282400 3.51039800 -0.53745600
```

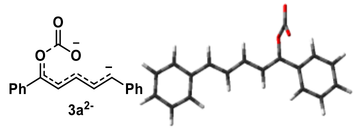

$3a^{2-}$

```
-2 1
C 3.08164700 -2.02751500 0.14886800
C 3.14812100 -0.65843200 -0.18173000
C 4.43482200 -0.12673500 -0.38702400
C 5.57271400 -0.91033400 -0.27781900
C 5.48281000 -2.25763700 0.04238100
C 4.22026600 -2.80360800 0.25504300
H 2.12260600 -2.49430900 0.33332100
H 4.53364100 0.91987600 -0.64153200
H 6.54381000 -0.45837900 -0.44564300
H 6.37208900 -2.86885600 0.12908000
H 4.12210200 -3.85199700 0.51292000
C 1.97527700 0.18707900 -0.30756300
C 0.67973200 -0.22375800 -0.20947000
H 0.51740700 -1.28277600 -0.02685200
C -0.48604500 0.56104800 -0.31574000
H -0.38066300 1.62734300 -0.49425000
C -1.76675900 0.02184600 -0.18645200
H -1.79498500 -1.05309600 -0.00228600
C -2.97531400 0.67980100 -0.25983700
H -2.96001200 1.75133200 -0.44748800
C -4.28009200 0.09122900 -0.10511600
C -4.51276000 -1.27724900 0.15885400
C -5.43272200 0.90051700 -0.21361900
C -5.79224300 -1.78425200 0.30036300
H -3.67457400 -1.95627300 0.25783900
C -6.70949600 0.38834600 -0.07084500
H -5.30511400 1.95907000 -0.41545600
C -6.91016000 -0.96351700 0.18848000
H -5.92012700 -2.84188300 0.50361000
H -7.56126100 1.05320700 -0.16299000
H -7.90853500 -1.36636400 0.30127100
O 2.21682800 1.51491700 -0.63944400
C 2.44303400 2.45532300 0.38329400
O 2.41288700 2.05840000 1.55565900
O 2.65236400 3.59545700 -0.06295900
```

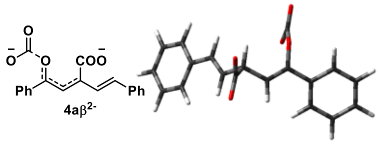

$4a\beta^{2-}$

```
-2 1
C -3.02178500 -0.42837400 -1.83930600
C -3.02595300 -0.24323300 -0.45567100
C -4.17390900 -0.59543100 0.25401300
C -5.28997100 -1.09571900 -0.39997800
C -5.27949600 -1.25906900 -1.77730500
C -4.13746000 -0.92460900 -2.49323100
H -2.13053100 -0.20043600 -2.40998500
H -4.18798400 -0.47183300 1.32886600
H -6.17196900 -1.35890500 0.17092000
H -6.14902500 -1.65254400 -2.28856000
H -4.11051300 -1.06332700 -3.56703200
C -1.85235900 0.30797500 0.26184900
C -0.94166000 1.10168700 -0.29994000
H -1.06225300 1.38376600 -1.33870300
C 1.50367900 1.03026200 -0.20463000
H 1.67579700 1.27299300 -1.24884200
C 2.34422700 0.24190500 0.46127900
H 2.12618700 0.02281900 1.50388800
C 3.56660700 -0.38823700 -0.06186900
C 4.02516700 -0.20463700 -1.37004100
C 4.31514300 -1.21082700 0.78211100
C 5.18370600 -0.82028200 -1.81121500
H 3.47362500 0.42755900 -2.05428400
C 5.47652600 -1.82880400 0.34210300
H 3.97829200 -1.36730000 1.80059600
C 5.91720500 -1.63633100 -0.95818600
H 5.51870200 -0.66193900 -2.82898100
H 6.03754000 -2.46183900 1.01846000
H 6.82330600 -2.11584400 -1.30628500
C 0.27290600 1.64413300 0.39413700
H 0.22527400 1.39820300 1.45319200
C 0.32009000 3.19768900 0.24681000
O 0.43649200 3.64287400 -0.91538100
O 0.23387200 3.85777900 1.30290800
O -1.80790200 0.04456300 1.60765200
C -1.39000600 -1.25221900 2.02957000
O -0.96291200 -2.00879300 1.15225800
O -1.51484900 -1.40725400 3.24803000
```

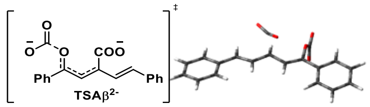

$TSA\beta^{2-}$

```
-2 1
C 3.17093000 -2.12934600 -0.27401200
C 3.23119700 -0.74108300 -0.07034100
C 4.50202400 -0.17956300 0.11557000
C 5.64626300 -0.96302800 0.11498500
C 5.56492000 -2.33476000 -0.07281800
C 4.31352800 -2.90875300 -0.26894100
H 2.21713800 -2.60920000 -0.45191700
H 4.58667500 0.88794200 0.26830400
H 6.61151600 -0.49338000 0.26417000
H 6.45806900 -2.94659000 -0.07429300
H 4.22761300 -3.97704600 -0.42936800
C 2.03990700 0.10857300 -0.04844600
C 0.76393500 -0.32828900 0.00358600
H 0.61315800 -1.40277900 0.04951200
C -1.69573400 -0.11605000 -0.07385300
H -1.71722200 -1.19919400 0.05043800
C -2.87642900 0.51566700 -0.31938900
H -2.85680100 1.59110300 -0.47727000
C -4.18844300 -0.10124700 -0.38088000
C -4.43122900 -1.46552600 -0.13454000
C -5.30977600 0.68552000 -0.69985900
C -5.70560400 -1.99981000 -0.21202300
H -3.60949600 -2.12053400 0.12779800
C -6.58458800 0.14973700 -0.77355100
H -5.16527000 1.74308500 -0.89347000
C -6.79825000 -1.20141300 -0.53199500
H -5.84958600 -3.05606200 -0.01502000
H -7.41986400 0.79416600 -1.02263400
H -7.79347600 -1.62360000 -0.58845700
C -0.41515500 0.47395600 0.07549600
H -0.31510700 1.53029500 -0.15083800
C -0.57107600 0.98900700 2.34258300
O 0.12320000 0.20241900 2.88048300
O -1.30124300 1.91448400 2.31425700
O 2.27241100 1.46942100 0.04948800
C 2.53301800 2.21490700 -1.12102100
O 2.50927300 1.61227500 -2.20124900
O 2.75586200 3.41024800 -0.87801100
```

K. NMR SPECTRA

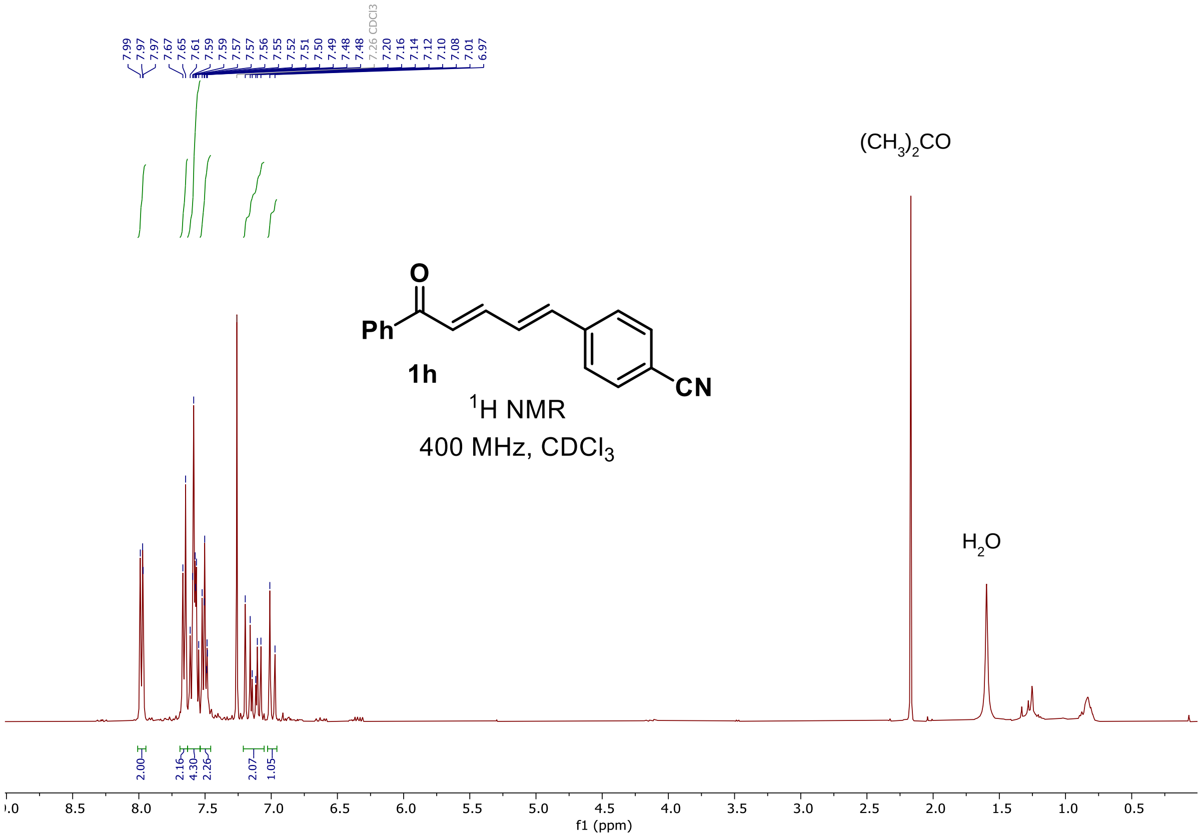

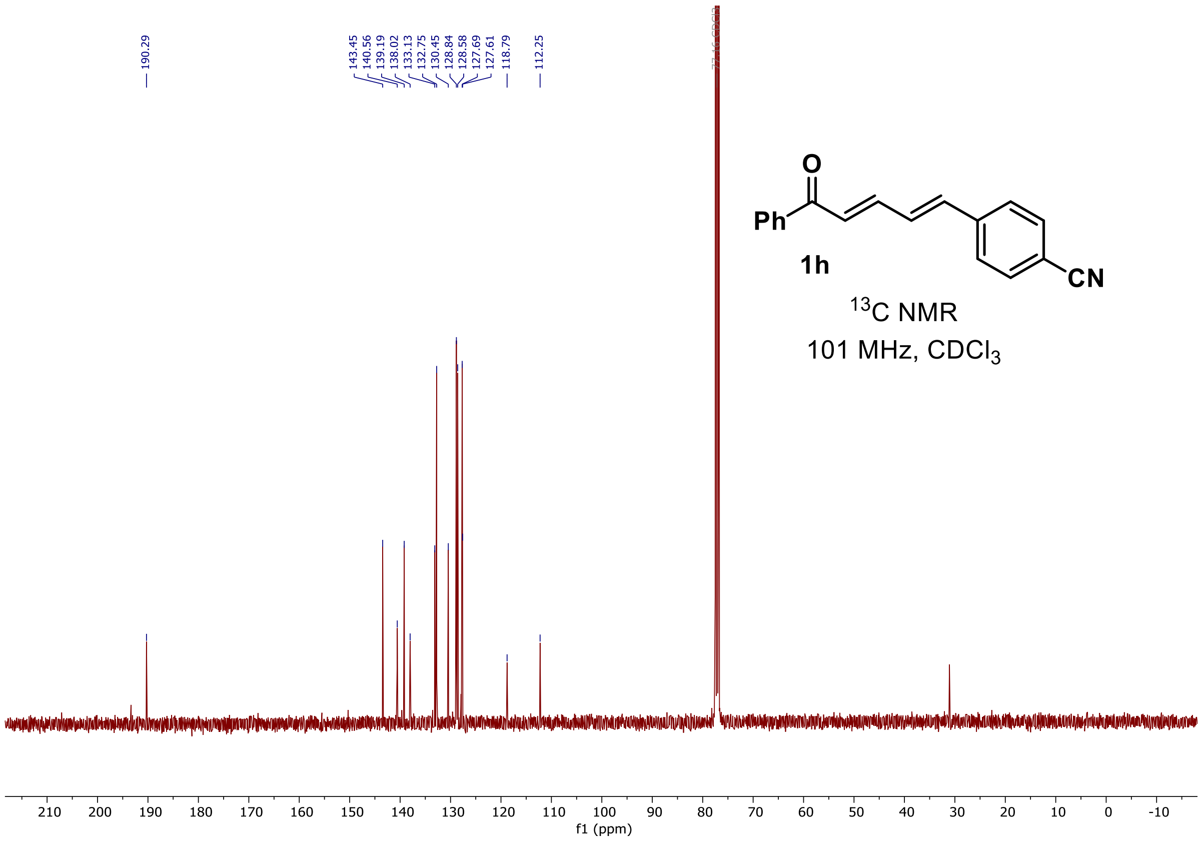
190.29
143.45
140.56
139.19
138.02
133.13
132.75
130.45
128.84
128.58
127.69
127.61
118.79
112.25
O
Ph
CN
1h
13C NMR
101 MHz, CDCl3
210 200 190 180 170 160 150 140 130 120 110 100 90 80 70 60 50 40 30 20 10 0 -10
f1 (ppm)

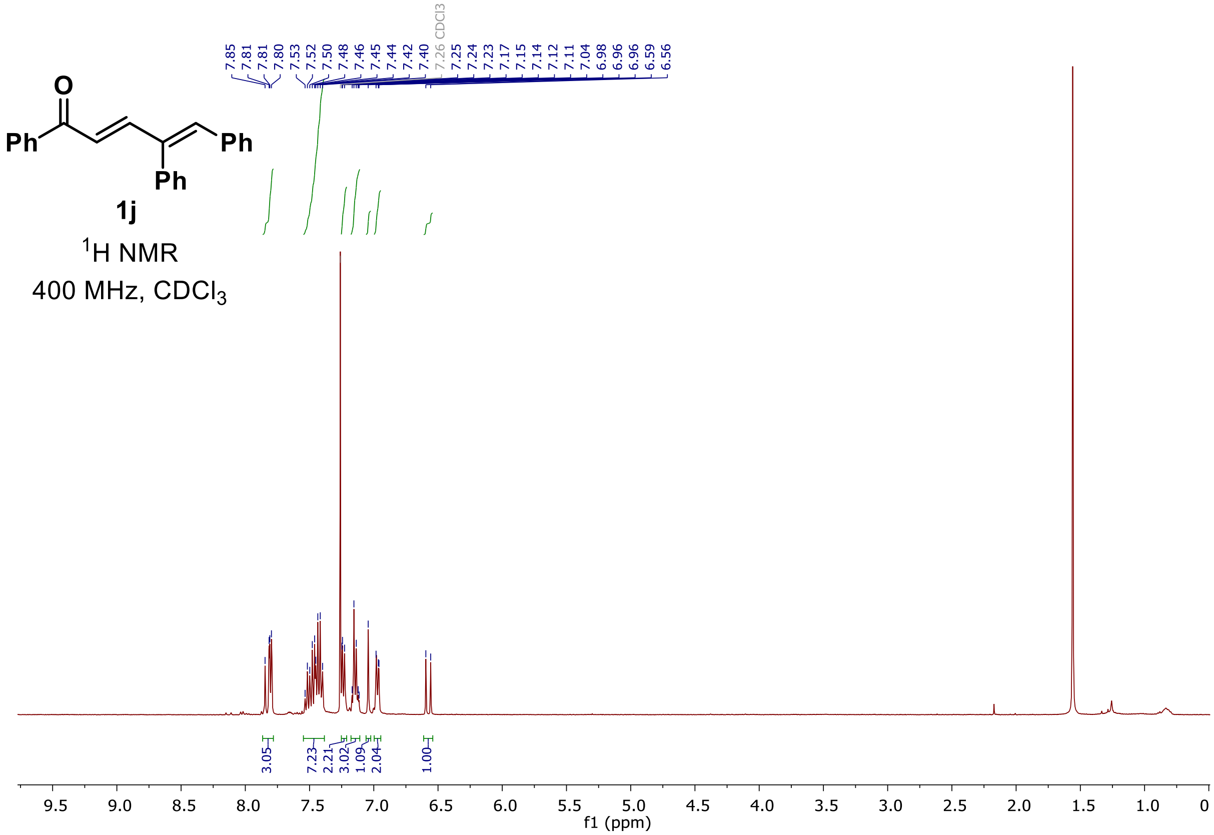
7.85
7.81
7.81
7.80
7.53
7.52
7.50
7.48
7.46
7.45
7.44
7.42
7.40
7.26 CDCl3
7.25
7.24
7.23
7.17
7.15
7.14
7.12
7.11
7.04
6.98
6.96
6.96
6.59
6.56
O
Ph
Ph
Ph
1j
1H NMR
400 MHz, CDCl3
3.05
7.23
2.21
3.02
1.09
2.04
1.00
9.5 9.0 8.5 8.0 7.5 7.0 6.5 6.0 5.5 5.0 4.5 4.0 3.5 3.0 2.5 2.0 1.5 1.0 0
f1 (ppm)

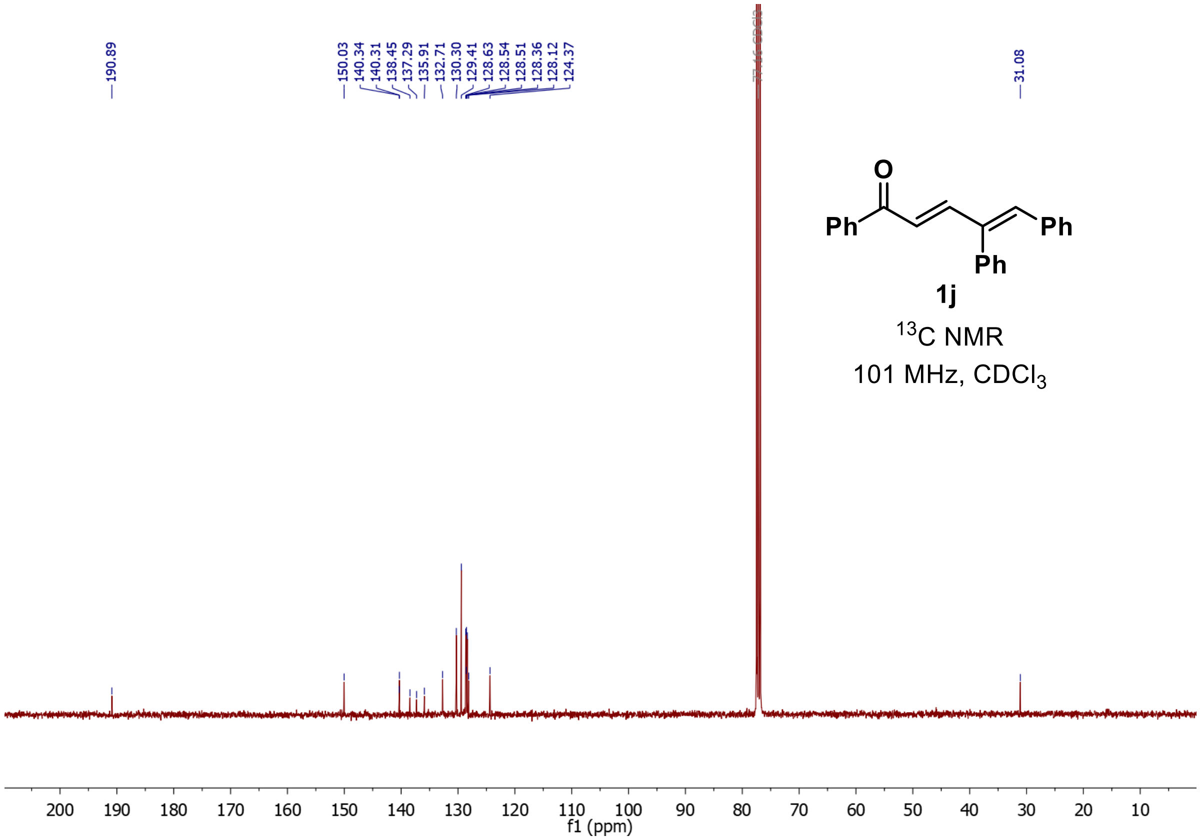

1j

^{13}C NMR

101 MHz, $CDCl_3$

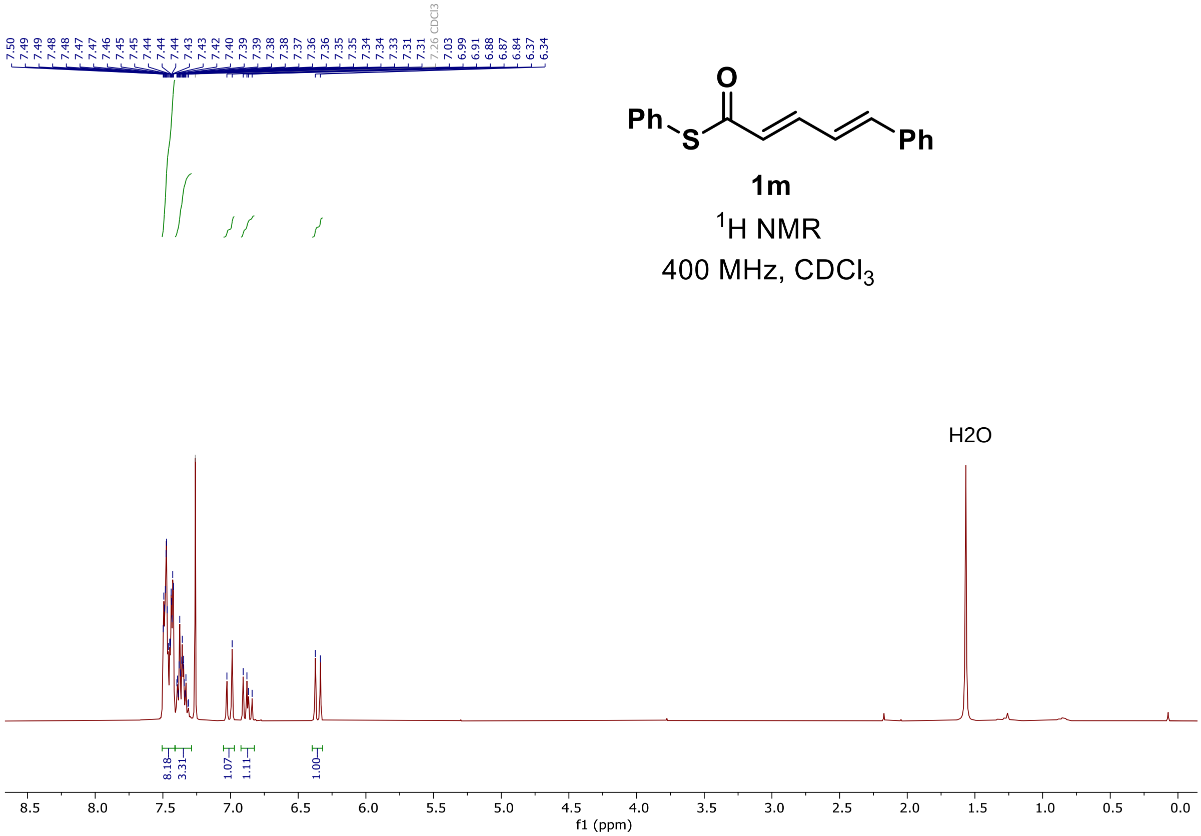

1m

^{1}H NMR

400 MHz, $CDCl_3$

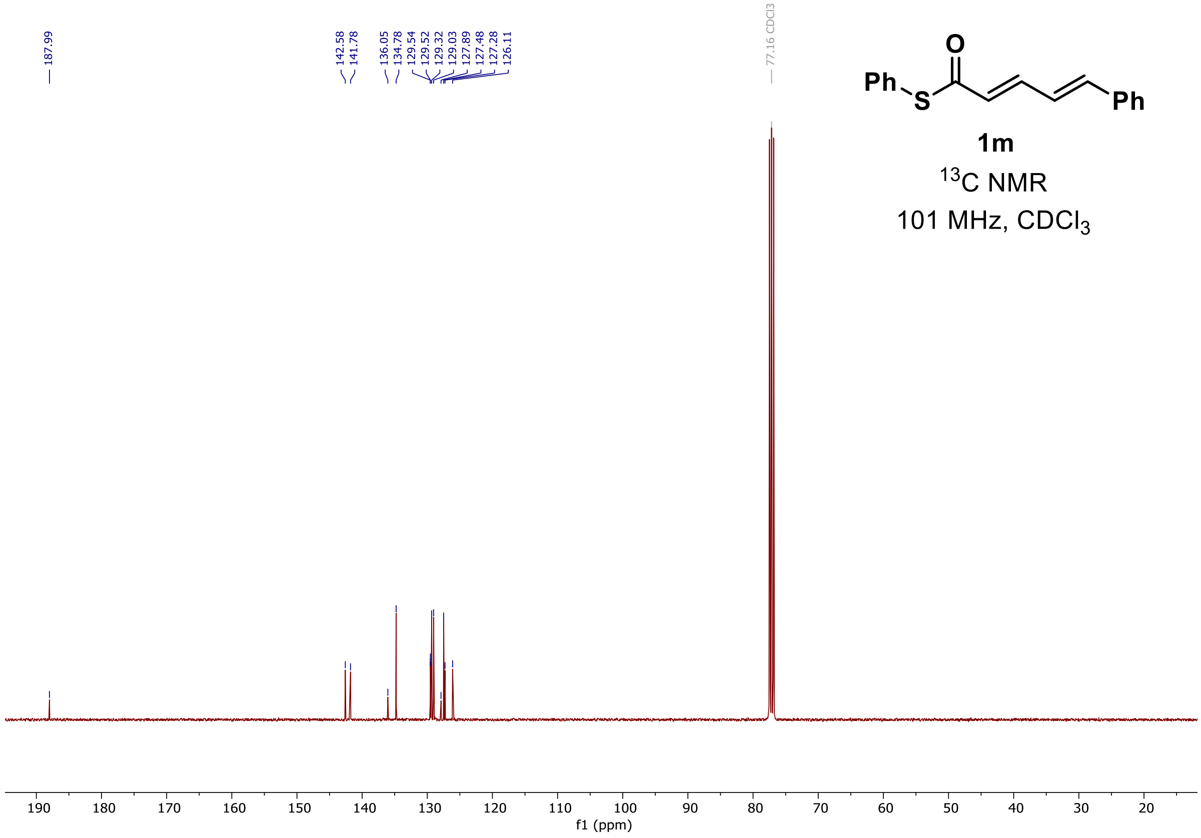

1m

^{13}C NMR

101 MHz, $CDCl_3$

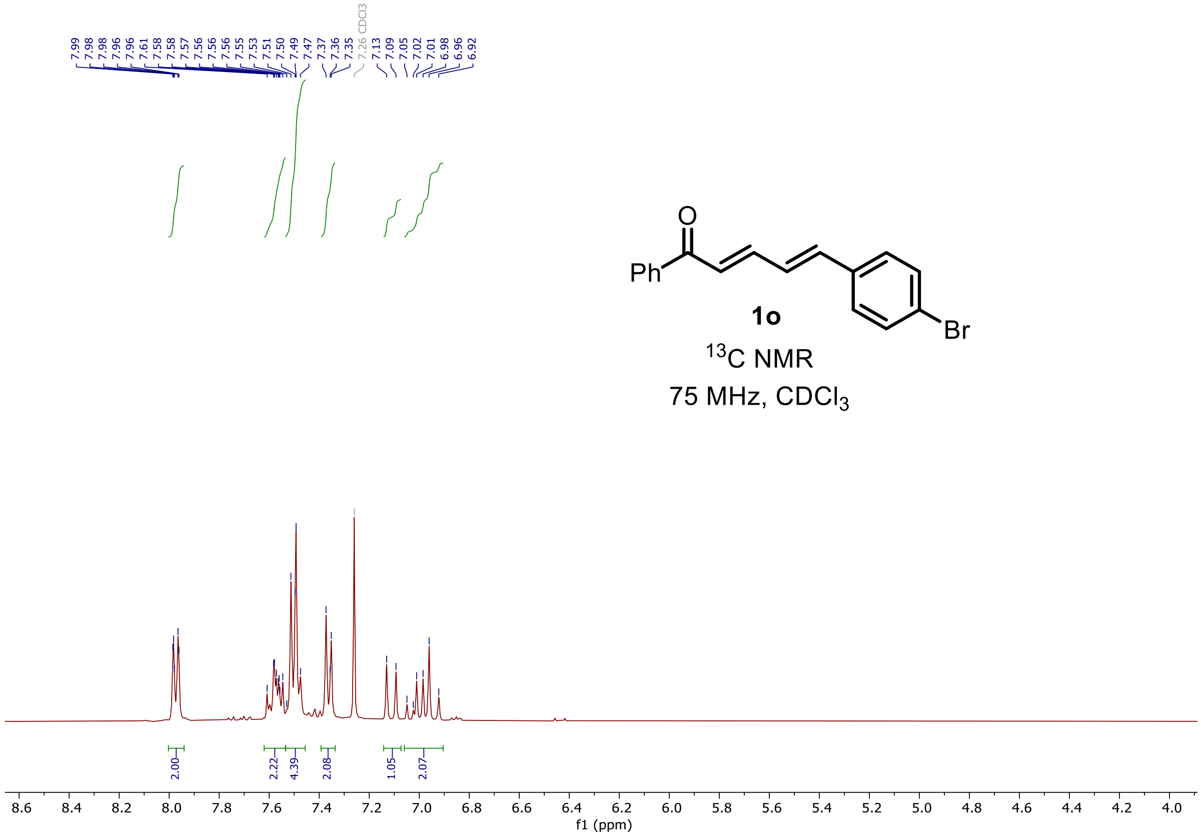

1o

^{13}C NMR

75 MHz, $CDCl_3$

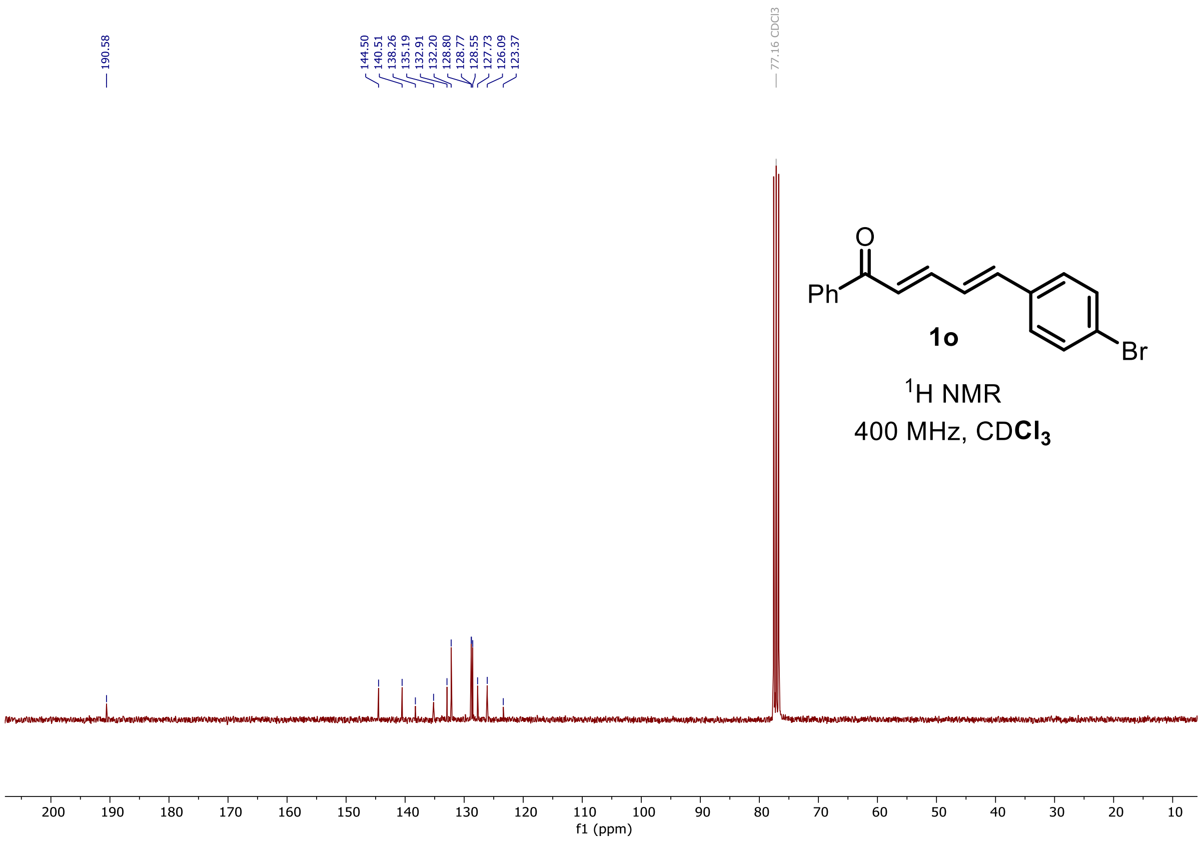

190.58
144.50
140.51
138.26
135.19
132.91
132.20
128.80
128.77
128.55
127.73
126.09
123.37
77.16 CDCl3
O
Ph
1o
Br
1H NMR
400 MHz, CDCl3
200 190 180 170 160 150 140 130 120 110 100 90 80 70 60 50 40 30 20 10
f1 (ppm)

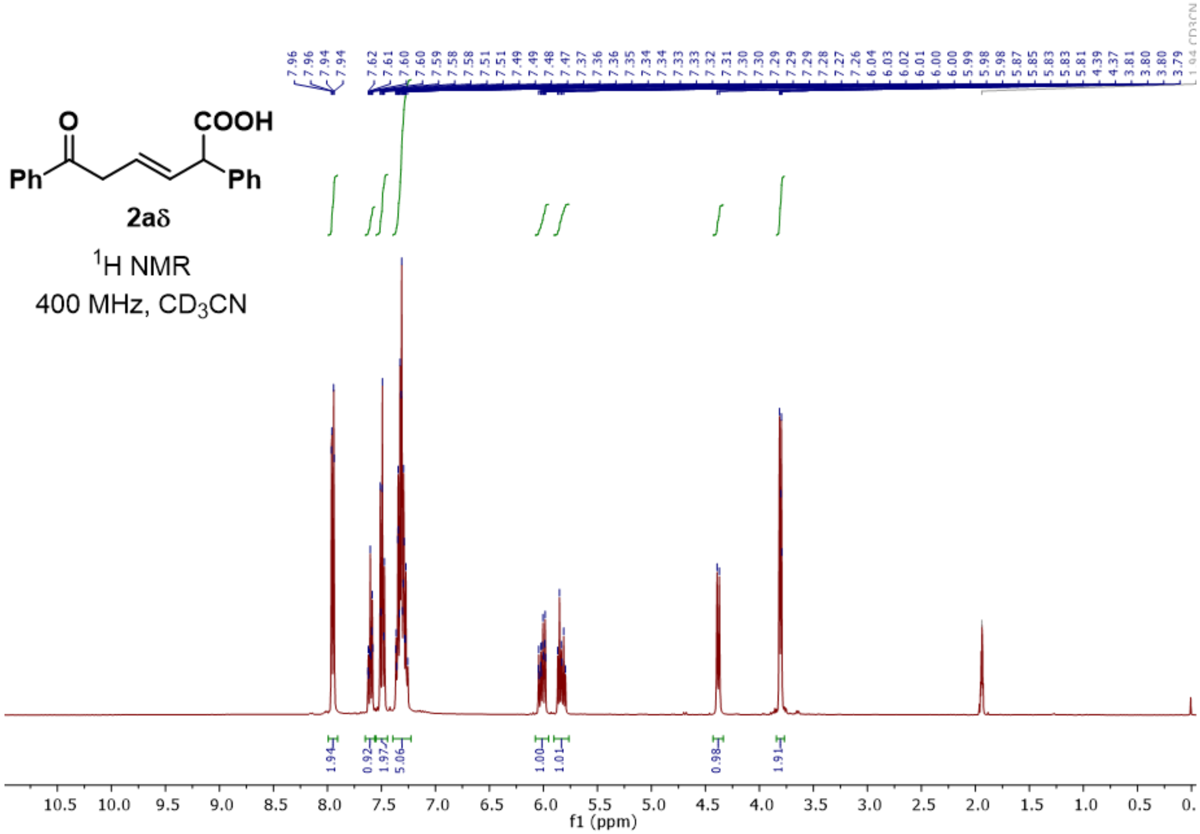
O
COOH
Ph
Ph
2aδ
1H NMR
400 MHz, CD3CN
f1 (ppm)

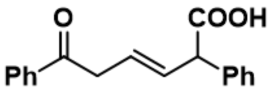

2aδ

^{13}C NMR

101 MHz, CD_3CN

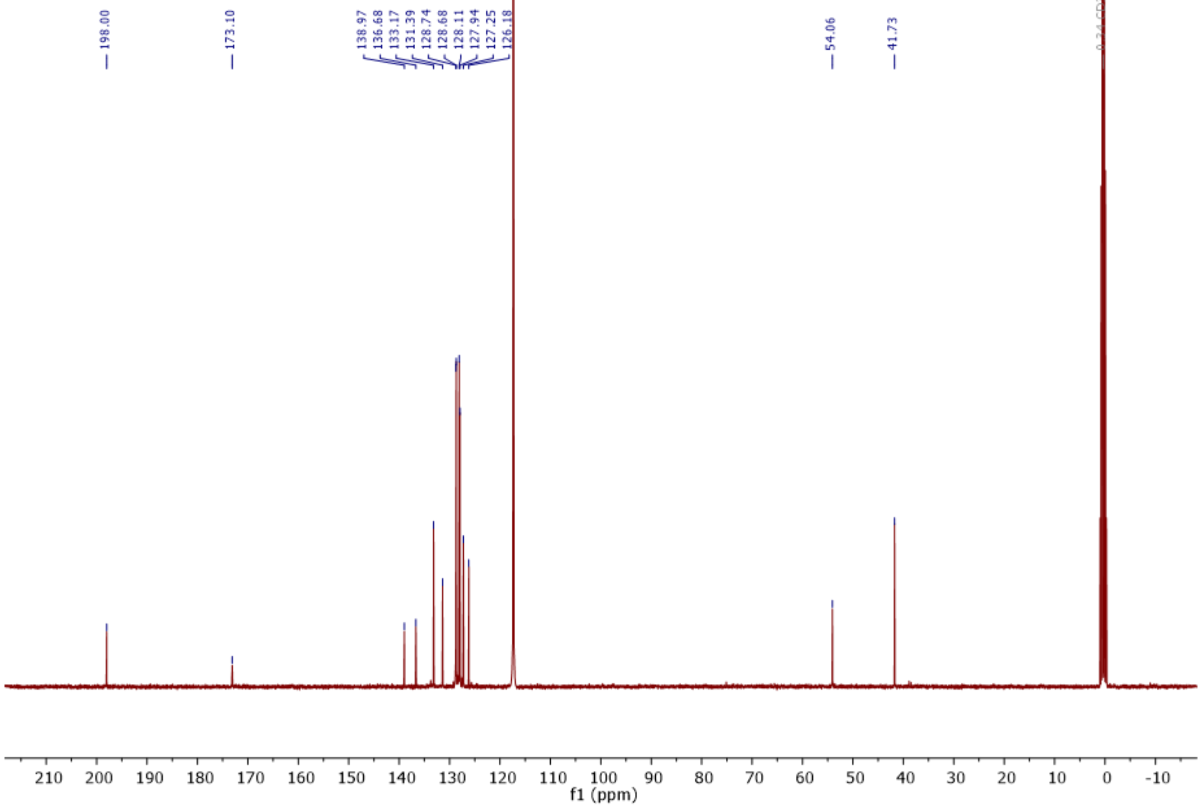

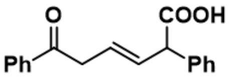

2aδ

^{1}H-^{1}H COSY-NMR
400 MHz, CD_3CN

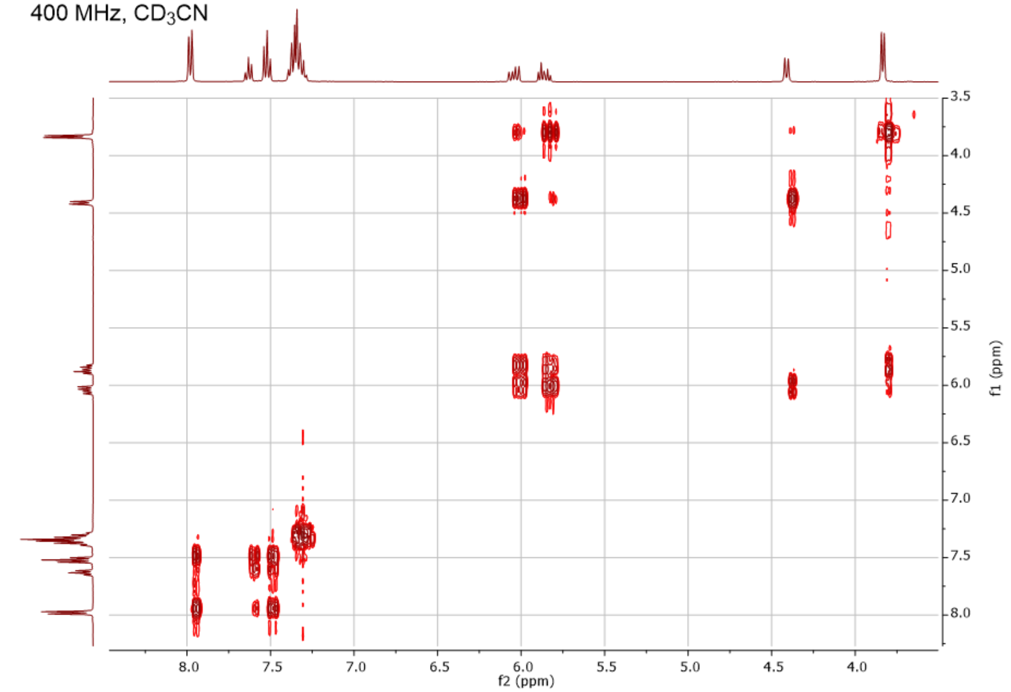

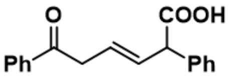

2aδ

^{1}H-^{13}C HMBC-NMR
CD_3CN

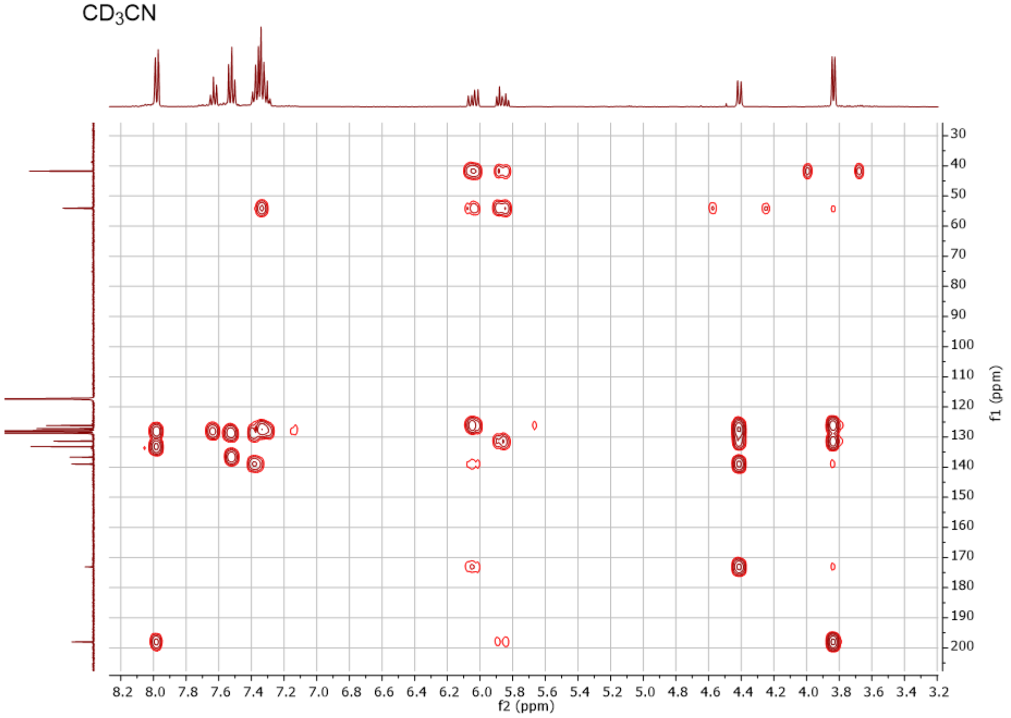

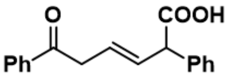

2aδ

^{1}H-^{1}H NOESY-NMR

400 MHz, CD_3CN

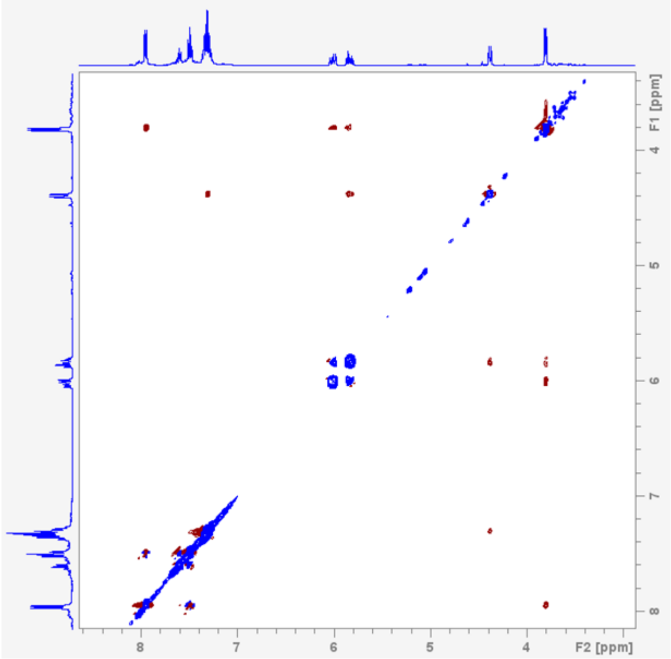

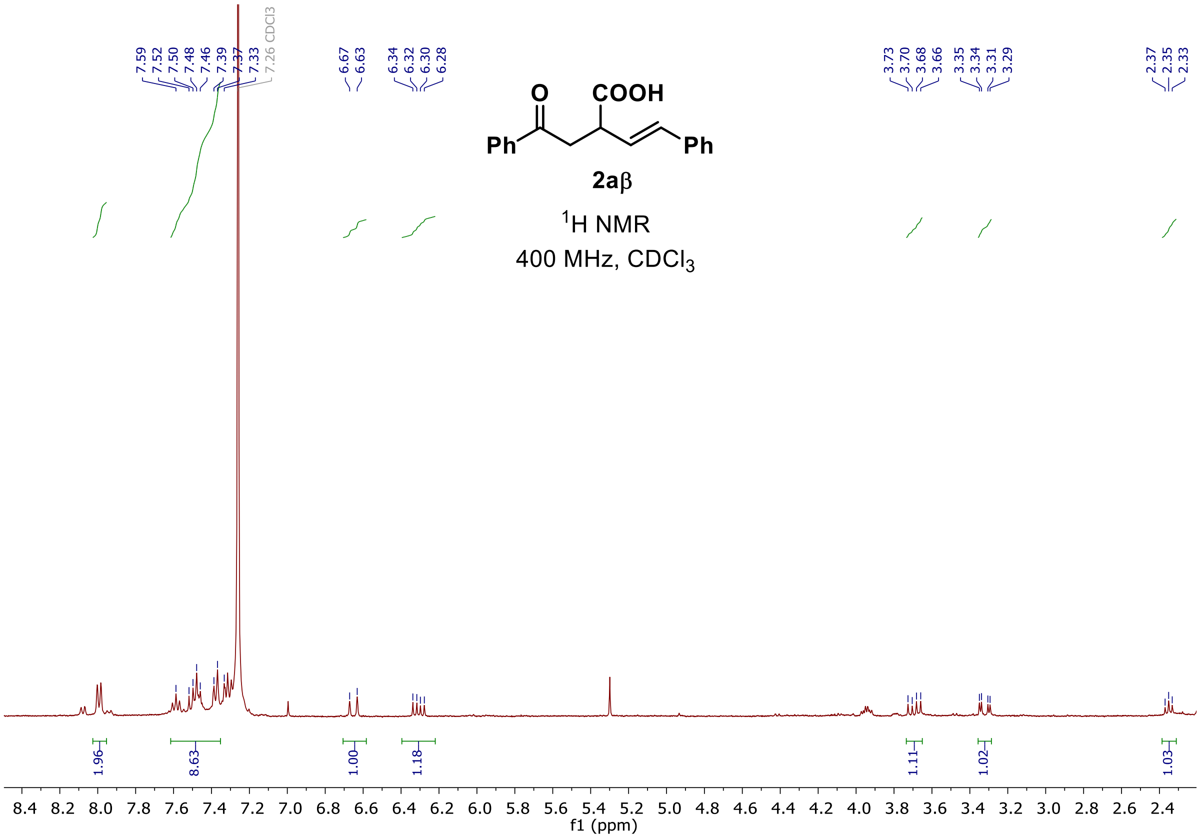

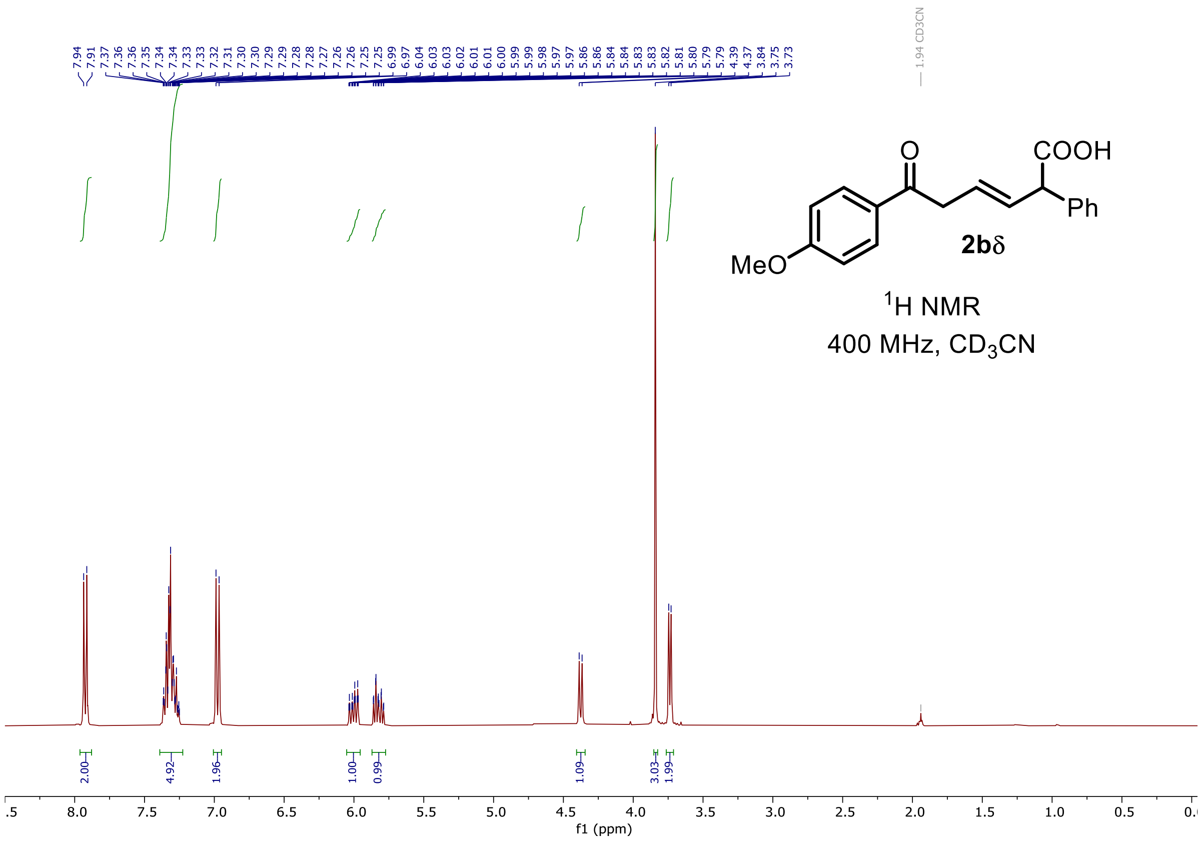
2bδ
^{1}H NMR
400 MHz, CD_3CN
f1 (ppm)

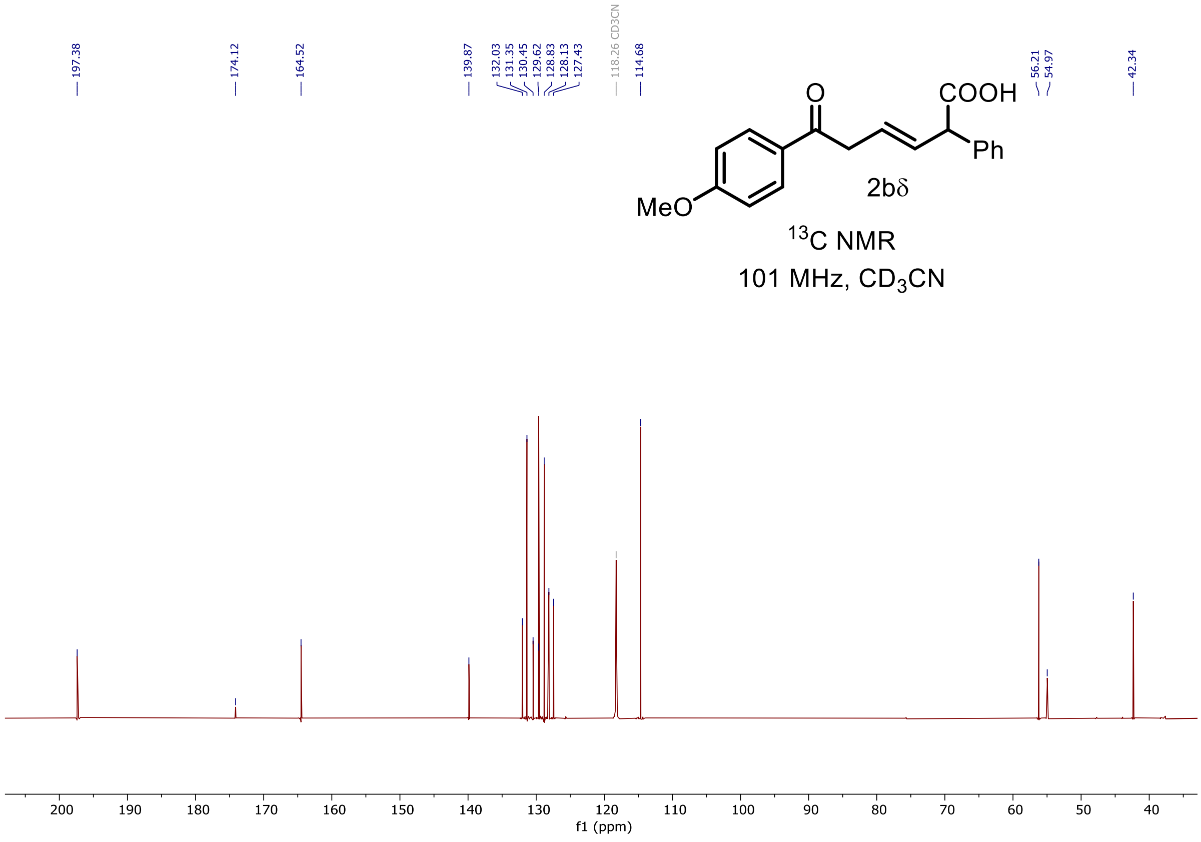
2bδ
^{13}C NMR
101 MHz, CD_3CN
f1 (ppm)

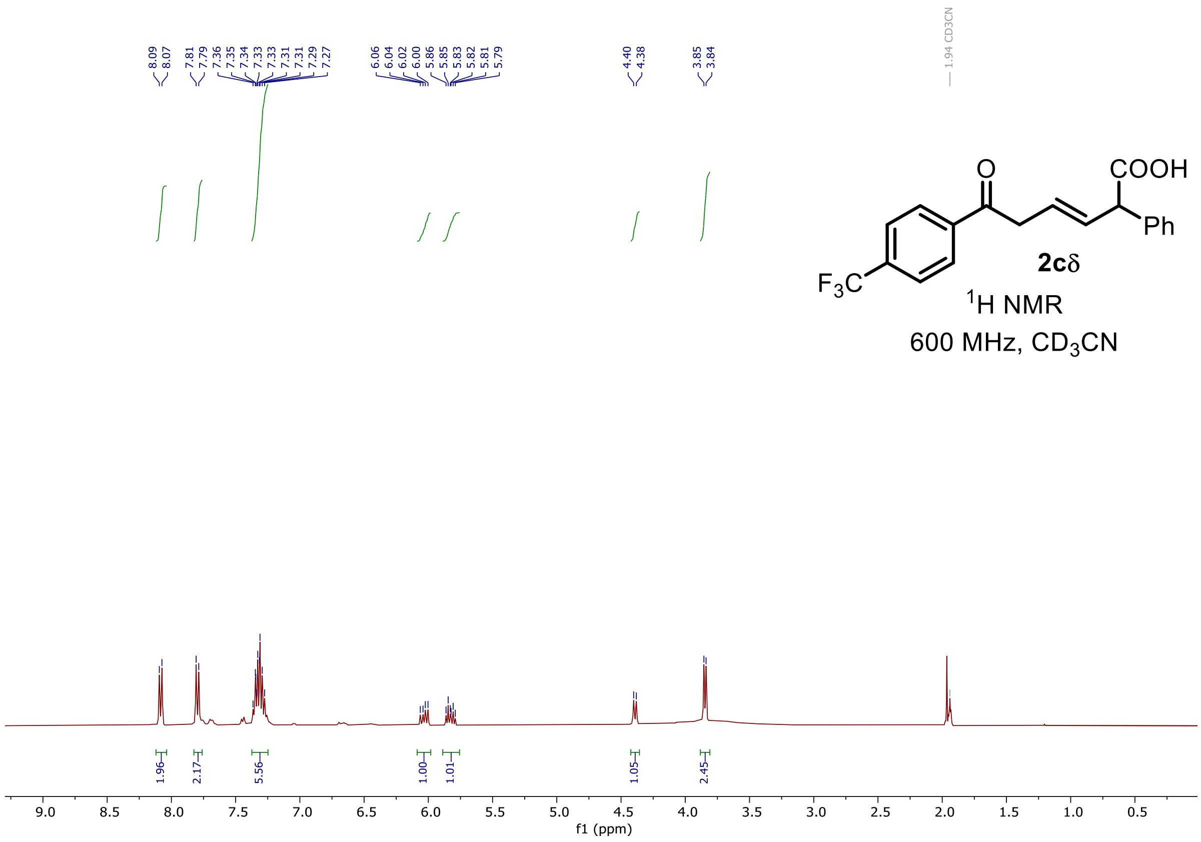

8.09
8.07
7.81
7.79
7.36
7.35
7.34
7.33
7.33
7.33
7.31
7.31
7.29
7.27
6.06
6.04
6.02
6.00
5.86
5.85
5.83
5.82
5.81
5.79
4.40
4.38
3.85
3.84
1.94 CD3CN
2cδ
^{1}H NMR
600 MHz, CD_3CN
1.96
2.17
5.56
1.00
1.01
1.05
2.45
f1 (ppm)

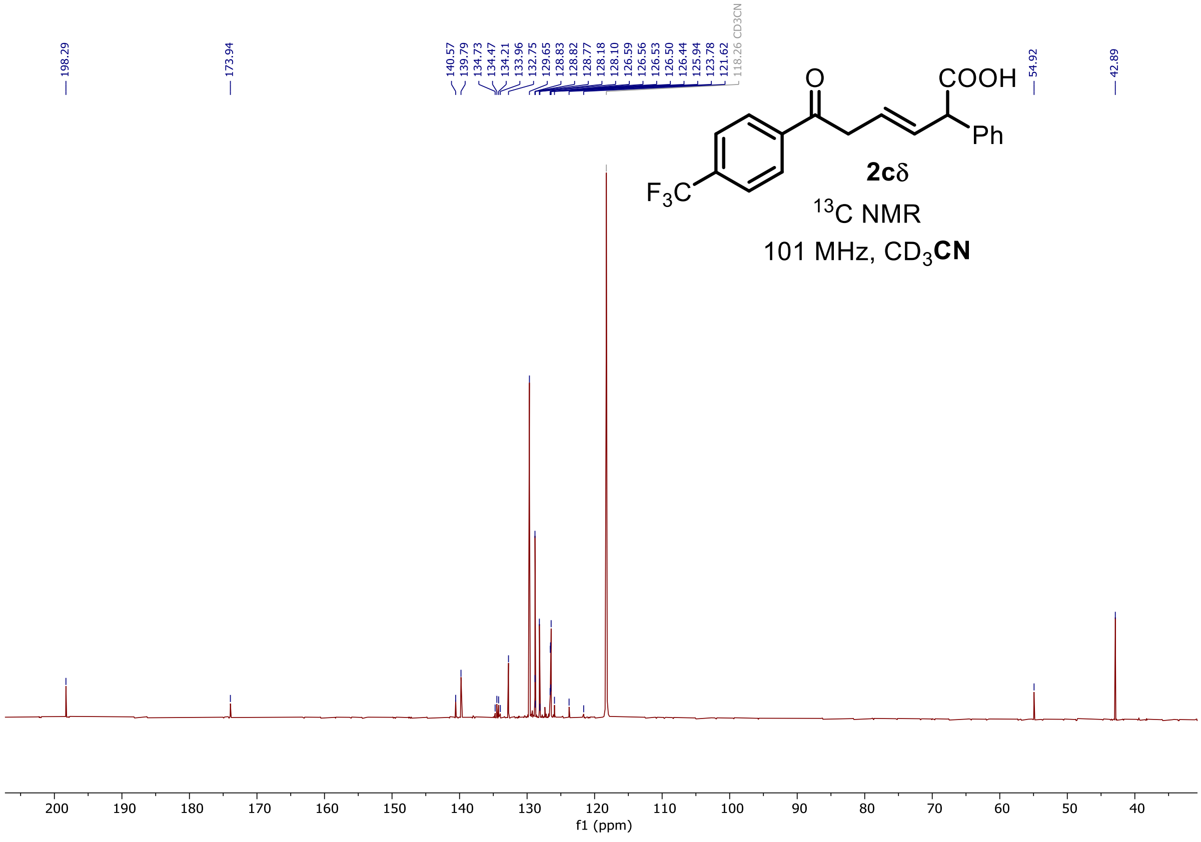

198.29
173.94
140.57
139.79
134.73
134.47
134.21
133.96
132.75
129.65
128.83
128.82
128.77
128.18
128.10
126.59
126.56
126.53
126.50
126.44
125.94
123.78
121.62
118.26 CD3CN
54.92
42.89
2cδ
^{13}C NMR
101 MHz, CD_3**CN**
f1 (ppm)

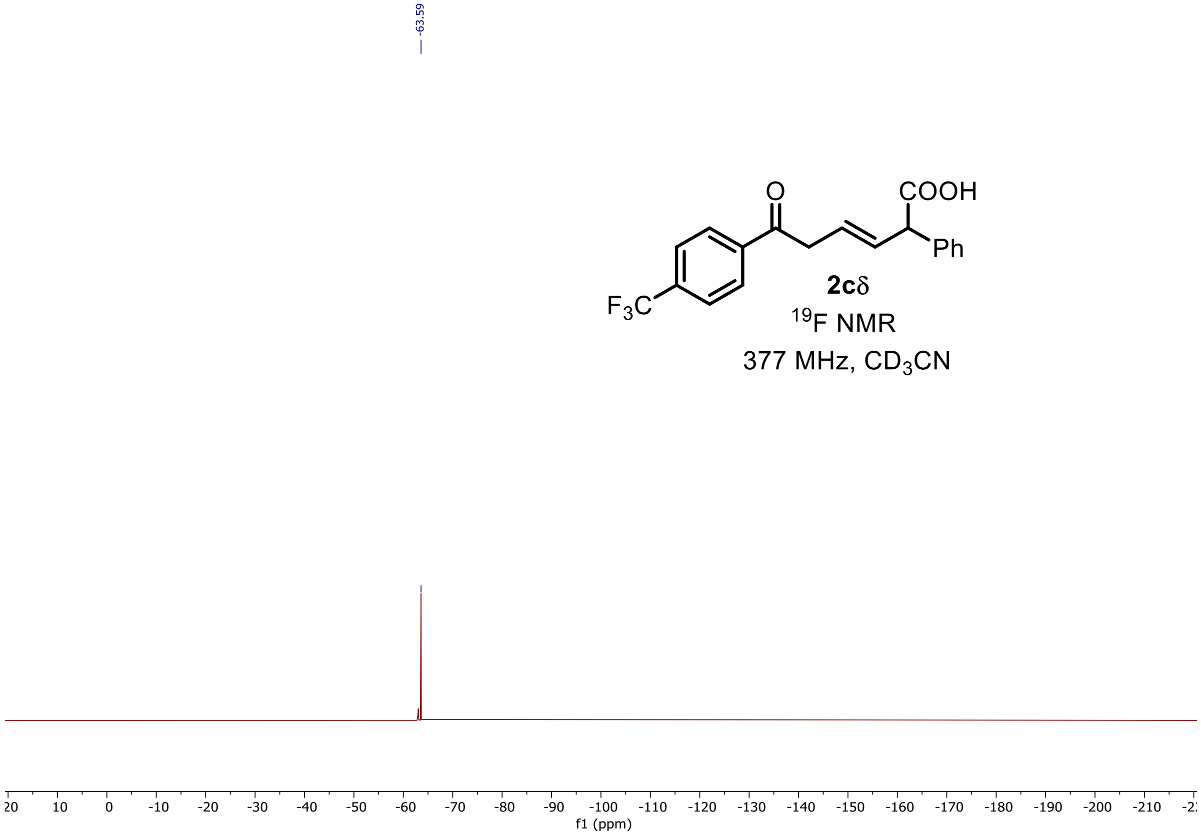

-63.59
O
COOH
Ph
F3C
2cδ
19F NMR
377 MHz, CD3CN
20 10 0 -10 -20 -30 -40 -50 -60 -70 -80 -90 -100 -110 -120 -130 -140 -150 -160 -170 -180 -190 -200 -210
f1 (ppm)

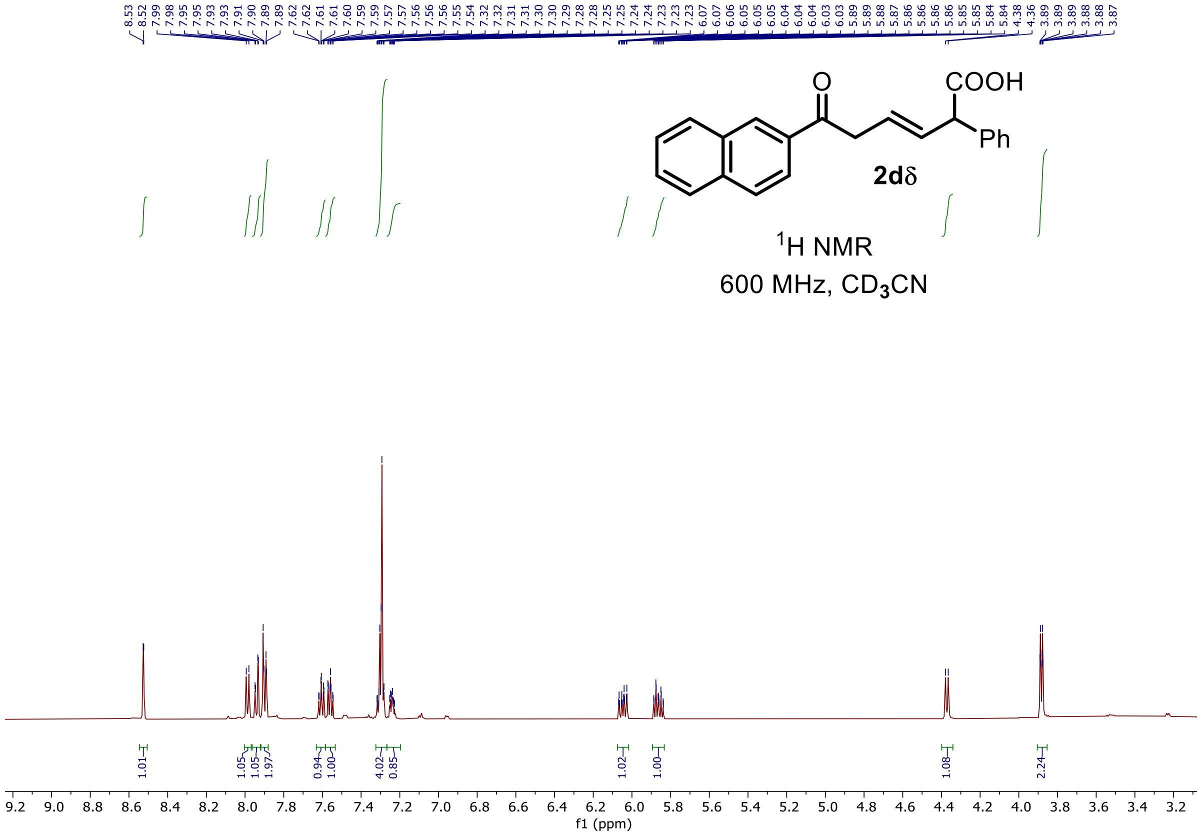

8.53 8.52 7.99 7.98 7.95 7.95 7.93 7.93 7.91 7.90 7.89 7.89 7.62 7.61 7.61 7.60 7.59 7.59 7.57 7.57 7.56 7.56 7.55 7.54 7.32 7.32 7.31 7.31 7.30 7.29 7.28 7.28 7.25 7.25 7.24 7.24 7.23 7.23 6.07 6.07 6.06 6.05 6.05 6.04 6.04 6.03 5.89 5.89 5.88 5.87 5.86 5.86 5.86 5.85 5.84 5.84 4.38 4.36 3.89 3.89 3.89 3.88 3.88 3.87
O
COOH
Ph
2dδ
1H NMR
600 MHz, CD3CN
1.01 1.05 1.05 1.97 0.94 1.00 4.02 0.85 1.02 1.00 1.08 2.24
9.2 9.0 8.8 8.6 8.4 8.2 8.0 7.8 7.6 7.4 7.2 7.0 6.8 6.6 6.4 6.2 6.0 5.8 5.6 5.4 5.2 5.0 4.8 4.6 4.4 4.2 4.0 3.8 3.6 3.4 3.2
f1 (ppm)

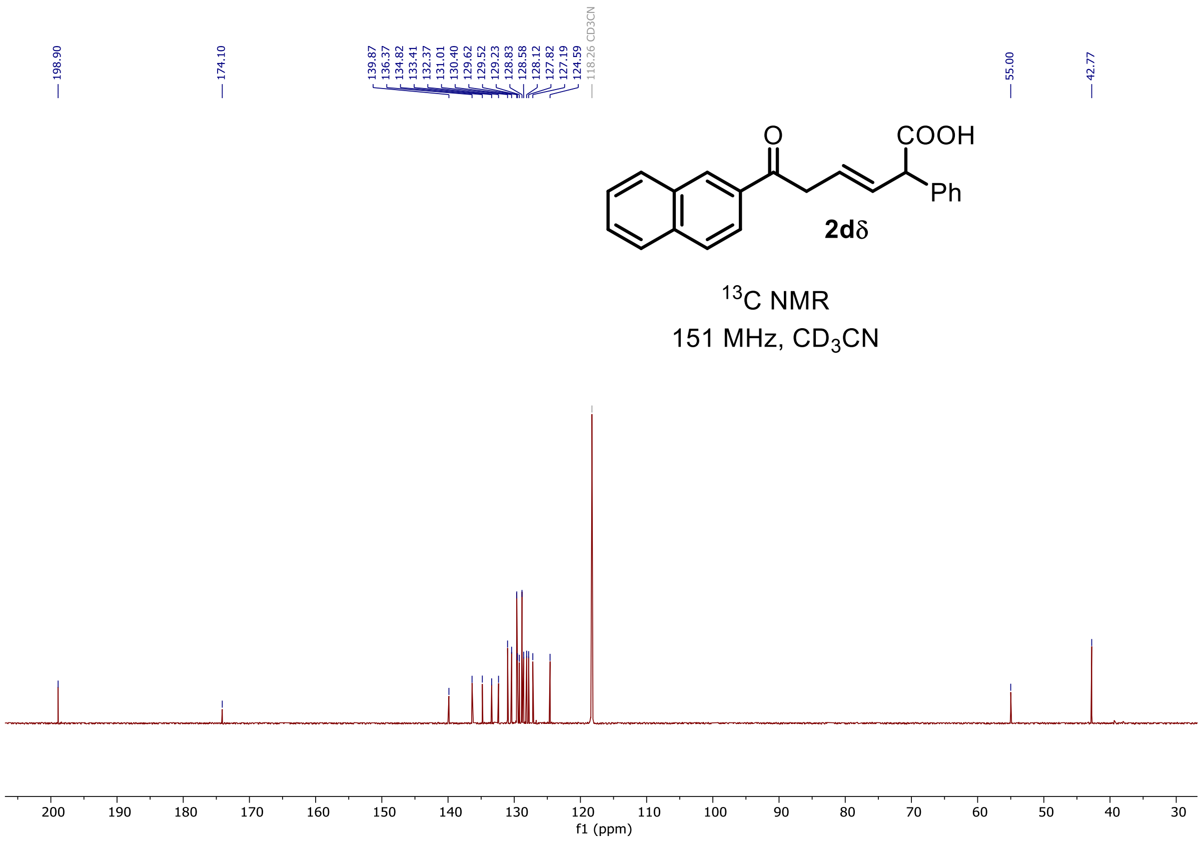
198.90
174.10
139.87
136.37
134.82
133.41
132.37
131.01
130.40
129.62
129.52
129.23
128.83
128.58
128.12
127.82
127.19
124.59
118.26 CD3CN
55.00
42.77
O
COOH
Ph
2dδ
^{13}C NMR
151 MHz, CD_3CN
f1 (ppm)

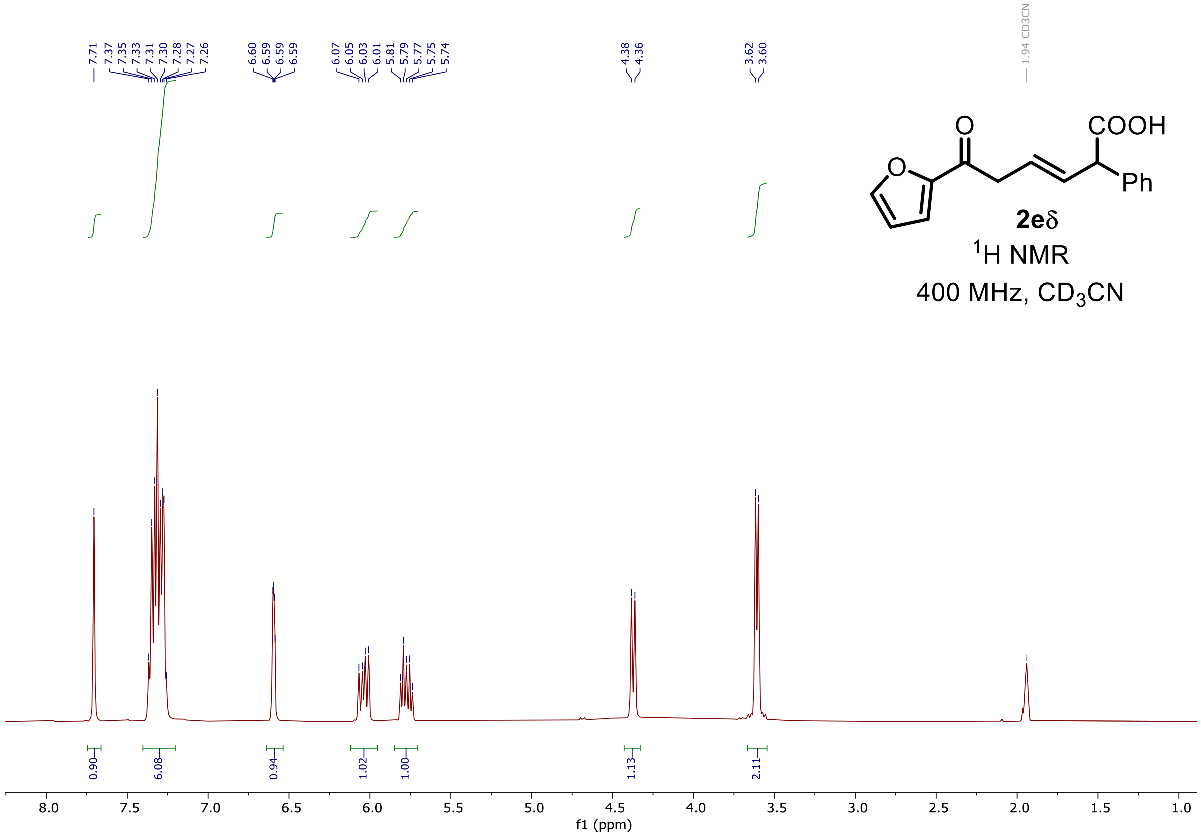
O
COOH
Ph
2eδ
^{1}H NMR
400 MHz, CD$_3$CN
7.71
7.37
7.35
7.33
7.31
7.30
7.28
7.27
7.26
6.60
6.59
6.59
6.59
6.07
6.05
6.03
6.01
5.81
5.79
5.77
5.75
5.74
4.38
4.36
3.62
3.60
1.94 CD3CN
0.90
6.08
0.94
1.02
1.00
1.13
2.11
8.0 7.5 7.0 6.5 6.0 5.5 5.0 4.5 4.0 3.5 3.0 2.5 2.0 1.5 1.0
f1 (ppm)

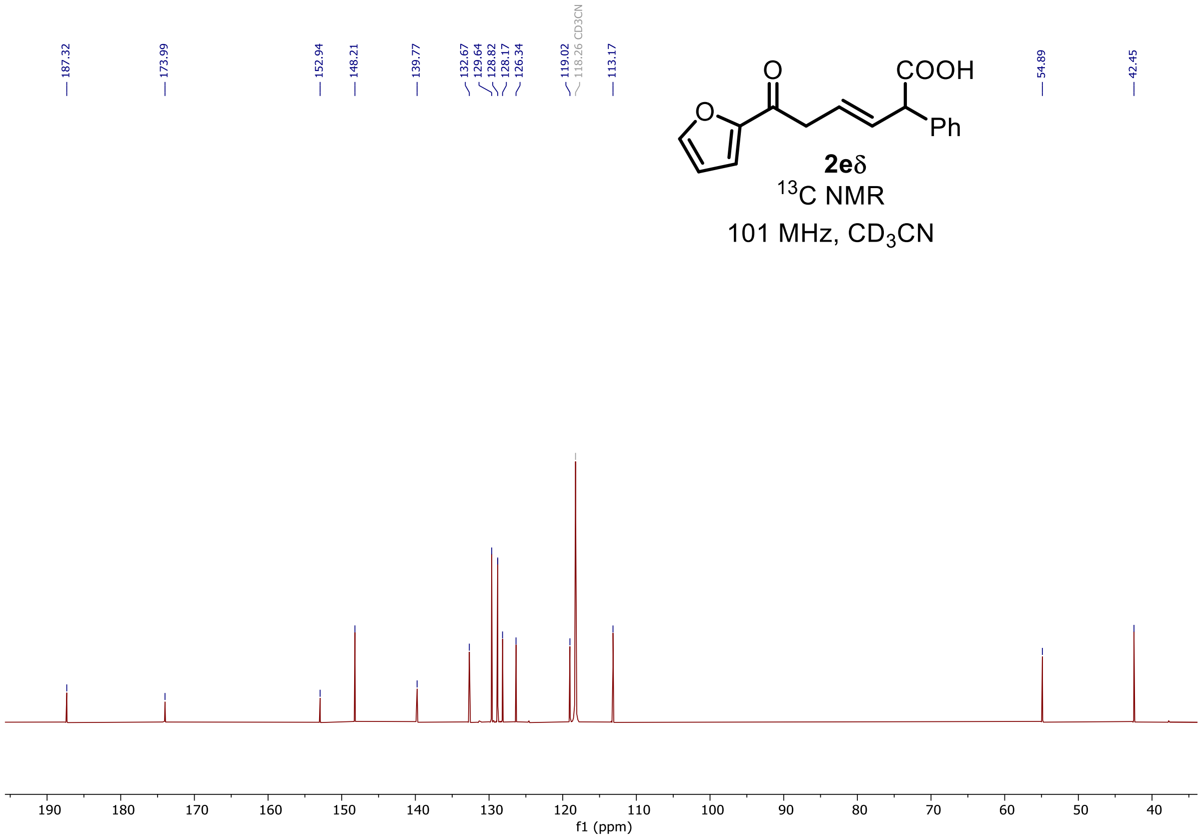
O
COOH
Ph
2eδ
^{13}C NMR
101 MHz, CD$_3$CN
187.32
173.99
152.94
148.21
139.77
132.67
129.64
128.82
128.17
126.34
119.02
118.26 CD3CN
113.17
54.89
42.45
190 180 170 160 150 140 130 120 110 100 90 80 70 60 50 40
f1 (ppm)

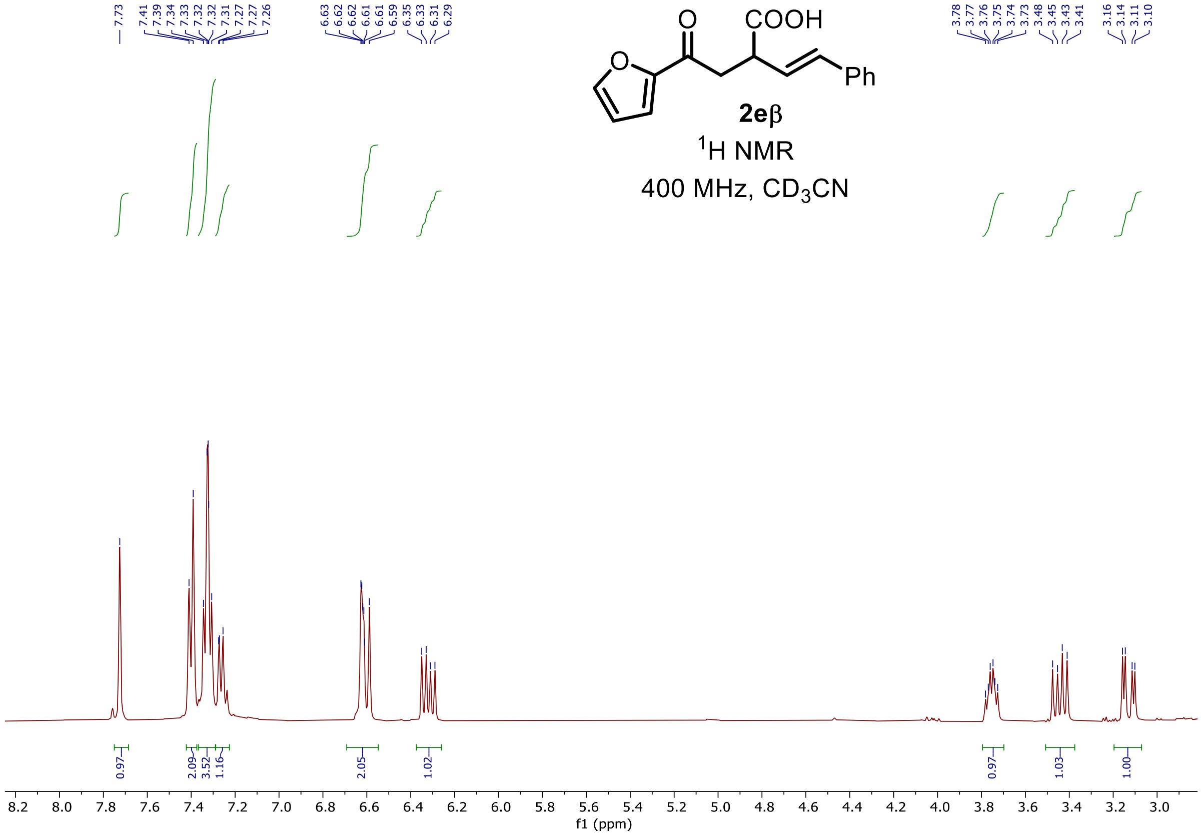

O
COOH
O
Ph
2eβ
1H NMR
400 MHz, CD3CN
7.73
7.41 7.39 7.34 7.33 7.32 7.32 7.31 7.27 7.27 7.26
6.63 6.62 6.62 6.61 6.61 6.59 6.35 6.33 6.31 6.29
3.78 3.77 3.76 3.75 3.74 3.73 3.48 3.45 3.43 3.41
3.16 3.14 3.11 3.10
0.97
2.09
3.52
1.16
2.05
1.02
0.97
1.03
1.00
f1 (ppm)

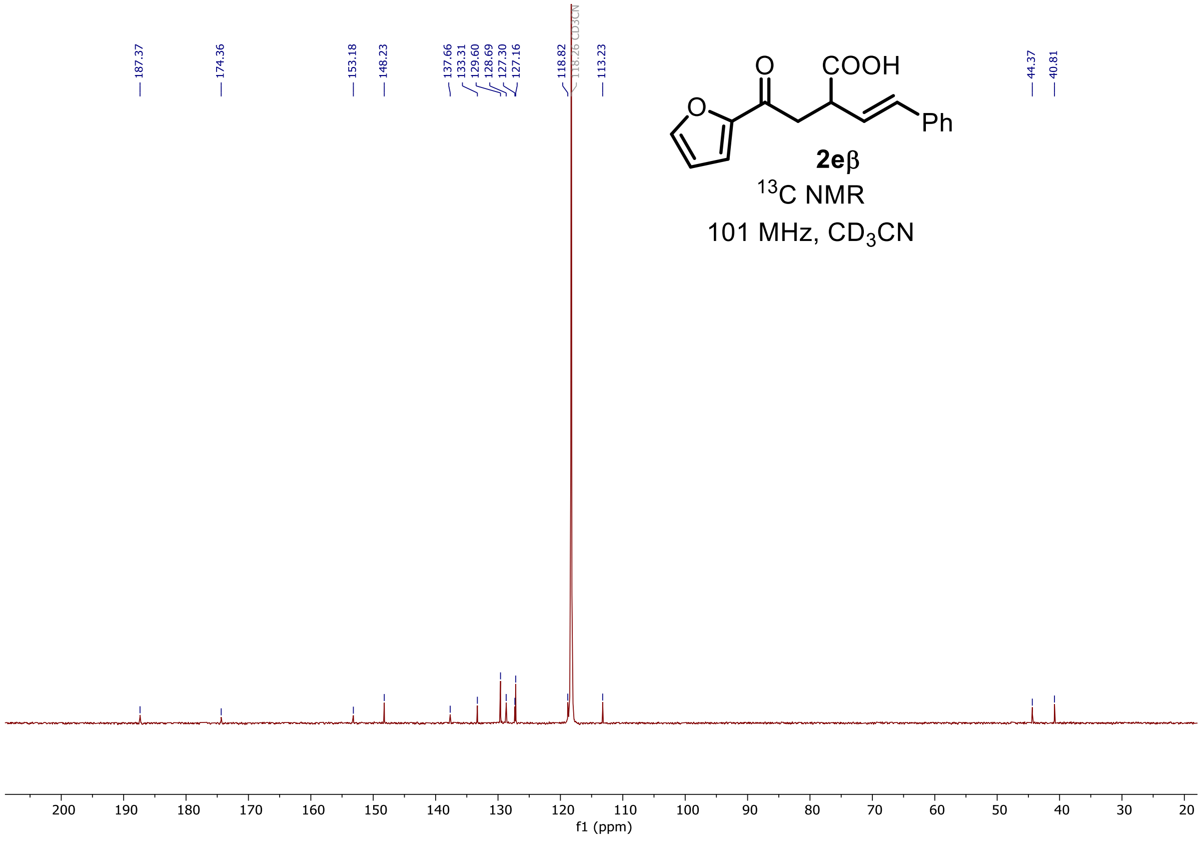

187.37
174.36
153.18
148.23
137.66 133.31 129.60 128.69 127.30 127.16
118.82
118.26 CD3CN
113.23
44.37
40.81
O
COOH
O
Ph
2eβ
13C NMR
101 MHz, CD3CN
f1 (ppm)

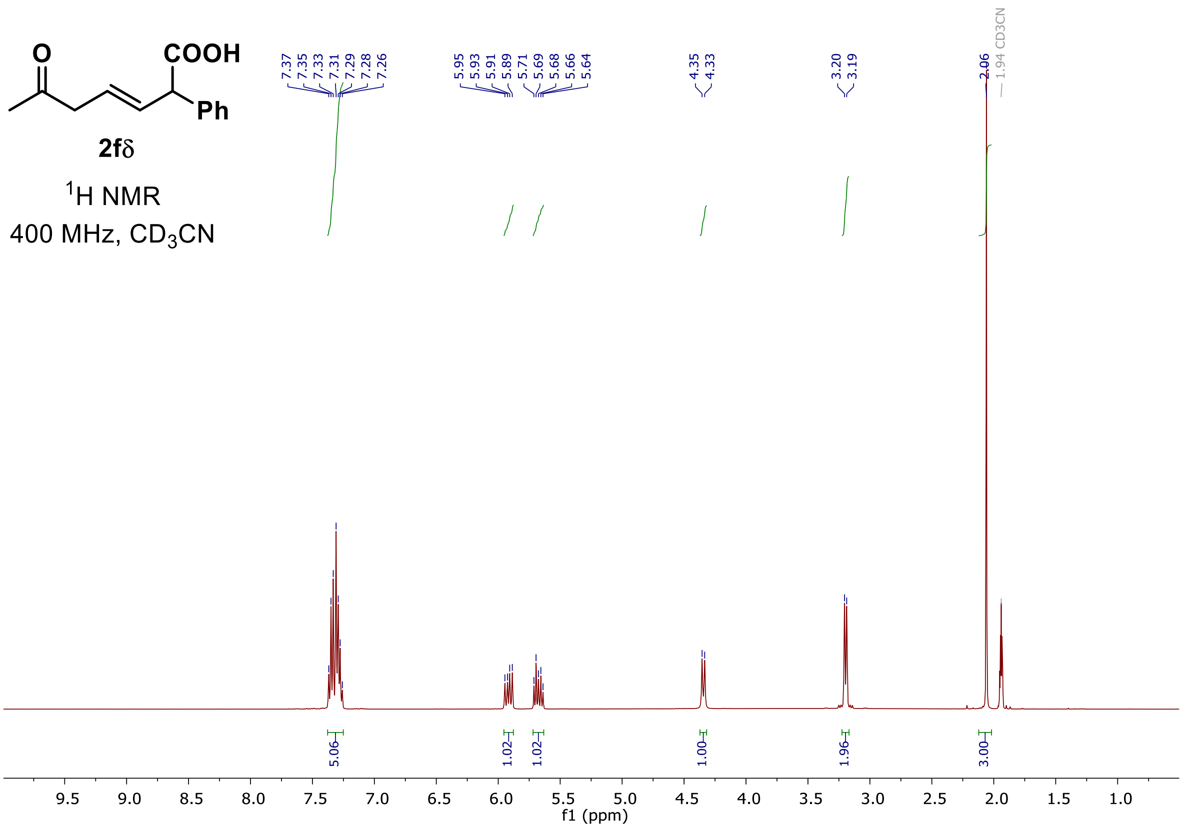
O
COOH
Ph
2fδ
1H NMR
400 MHz, CD3CN
7.37
7.35
7.33
7.31
7.29
7.28
7.26
5.95
5.93
5.91
5.89
5.71
5.69
5.68
5.66
5.64
4.35
4.33
3.20
3.19
2.06
1.94 CD3CN
5.06
1.02
1.02
1.00
1.96
3.00
f1 (ppm)

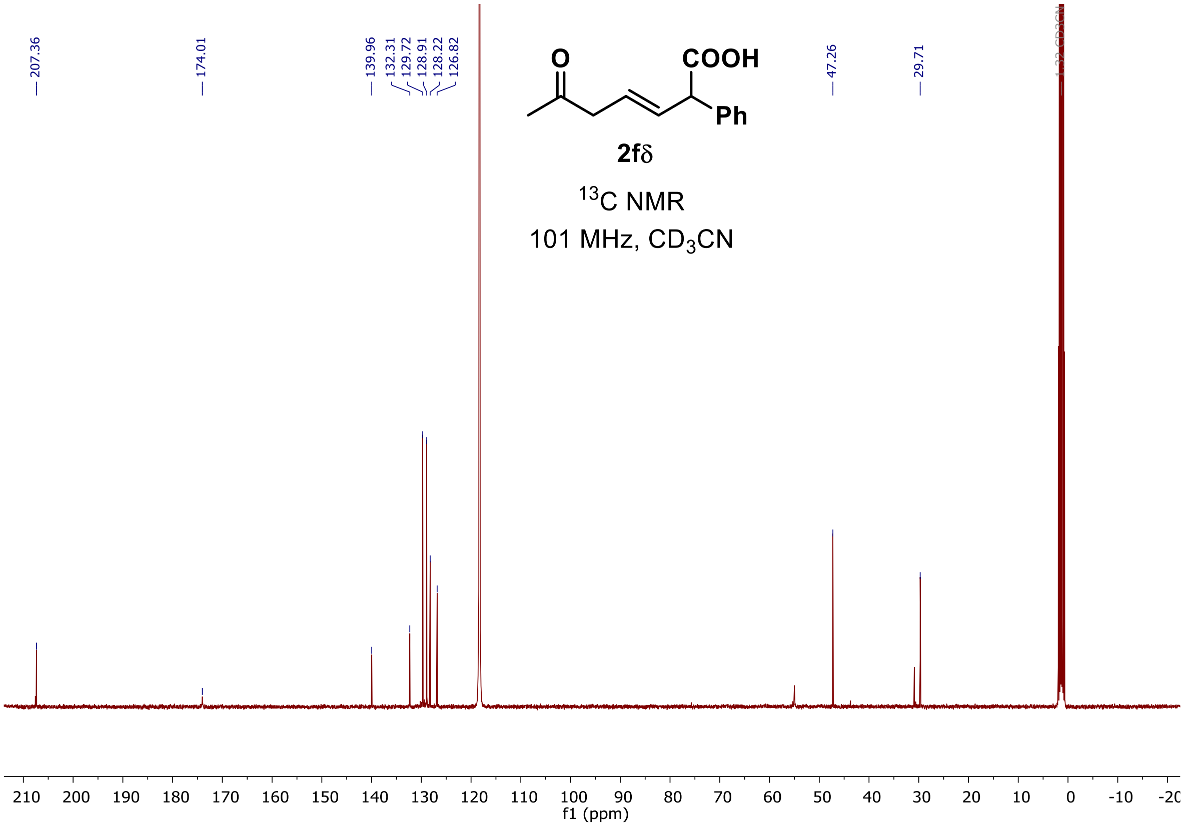
207.36
174.01
139.96
132.31
129.72
128.91
128.22
126.82
O
COOH
Ph
2fδ
13C NMR
101 MHz, CD3CN
47.26
29.71
f1 (ppm)

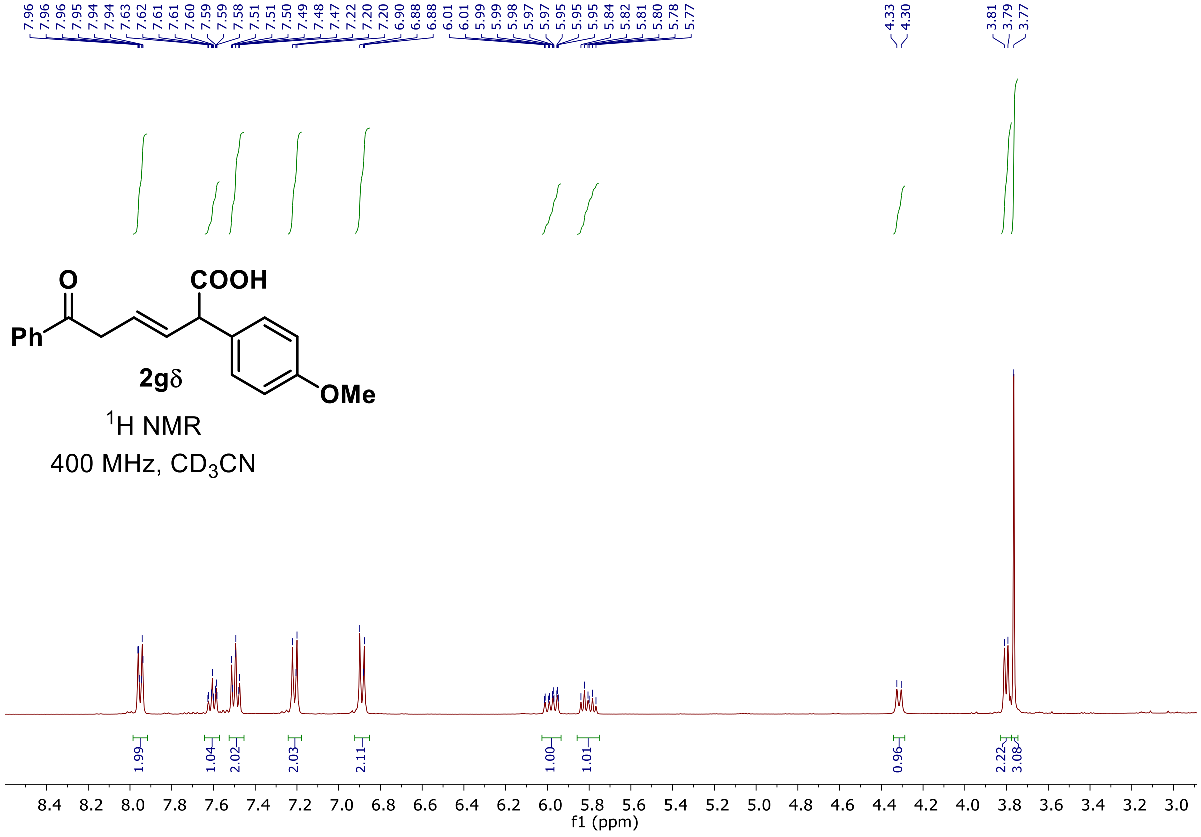
O
COOH
Ph
2gδ
OMe
1H NMR
400 MHz, CD3CN
7.96 7.96 7.95 7.94 7.94 7.63 7.62 7.61 7.61 7.60 7.59 7.59 7.58 7.51 7.51 7.50 7.49 7.48 7.47 7.22 7.20 7.20 6.90 6.88 6.88 6.01 6.01 5.99 5.99 5.98 5.97 5.97 5.95 5.95 5.95 5.84 5.82 5.81 5.80 5.78 5.77
4.33 4.30
3.81 3.79 3.77
1.99 1.04 2.02 2.03 2.11 1.00 1.01 0.96 2.22 3.08
8.4 8.2 8.0 7.8 7.6 7.4 7.2 7.0 6.8 6.6 6.4 6.2 6.0 5.8 5.6 5.4 5.2 5.0 4.8 4.6 4.4 4.2 4.0 3.8 3.6 3.4 3.2 3.0
f1 (ppm)

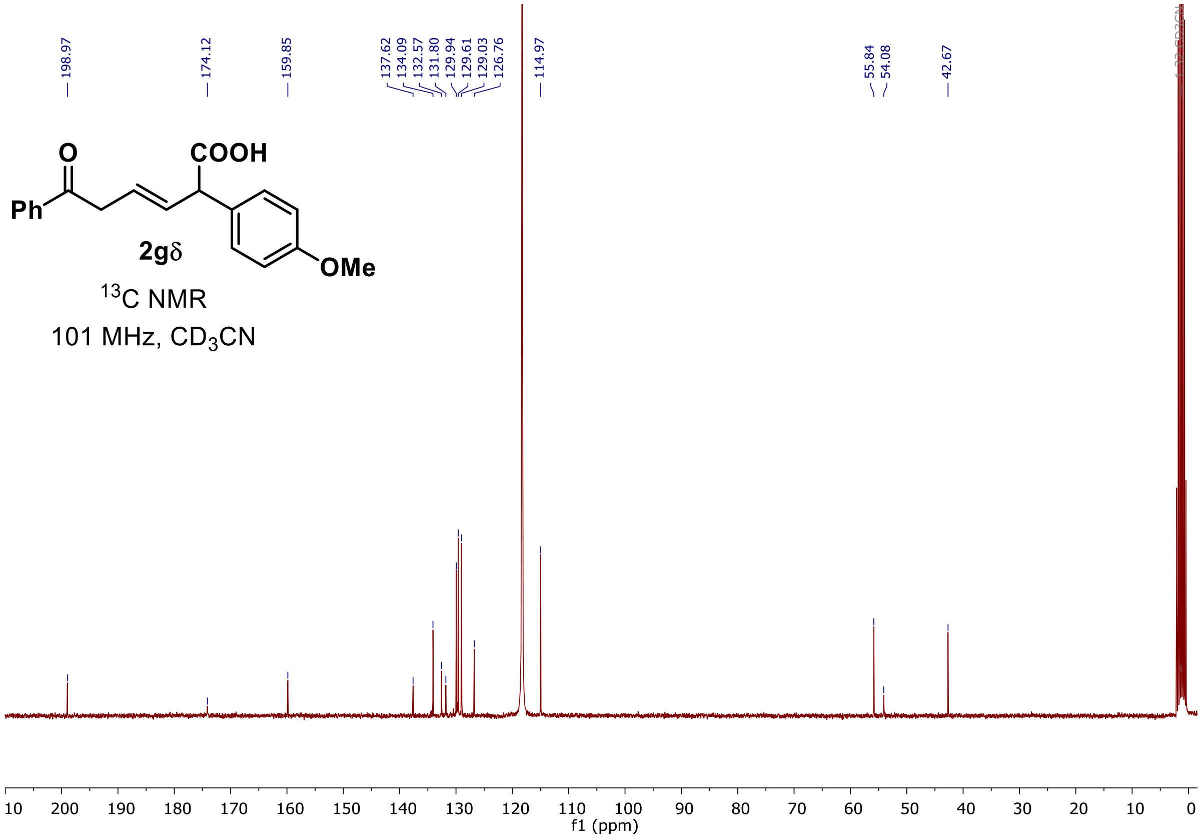
198.97
174.12
159.85
137.62 134.09 132.57 131.80 129.94 129.61 129.03 126.76
114.97
55.84 54.08
42.67
O
COOH
Ph
2gδ
OMe
13C NMR
101 MHz, CD3CN
10 200 190 180 170 160 150 140 130 120 110 100 90 80 70 60 50 40 30 20 10 0
f1 (ppm)

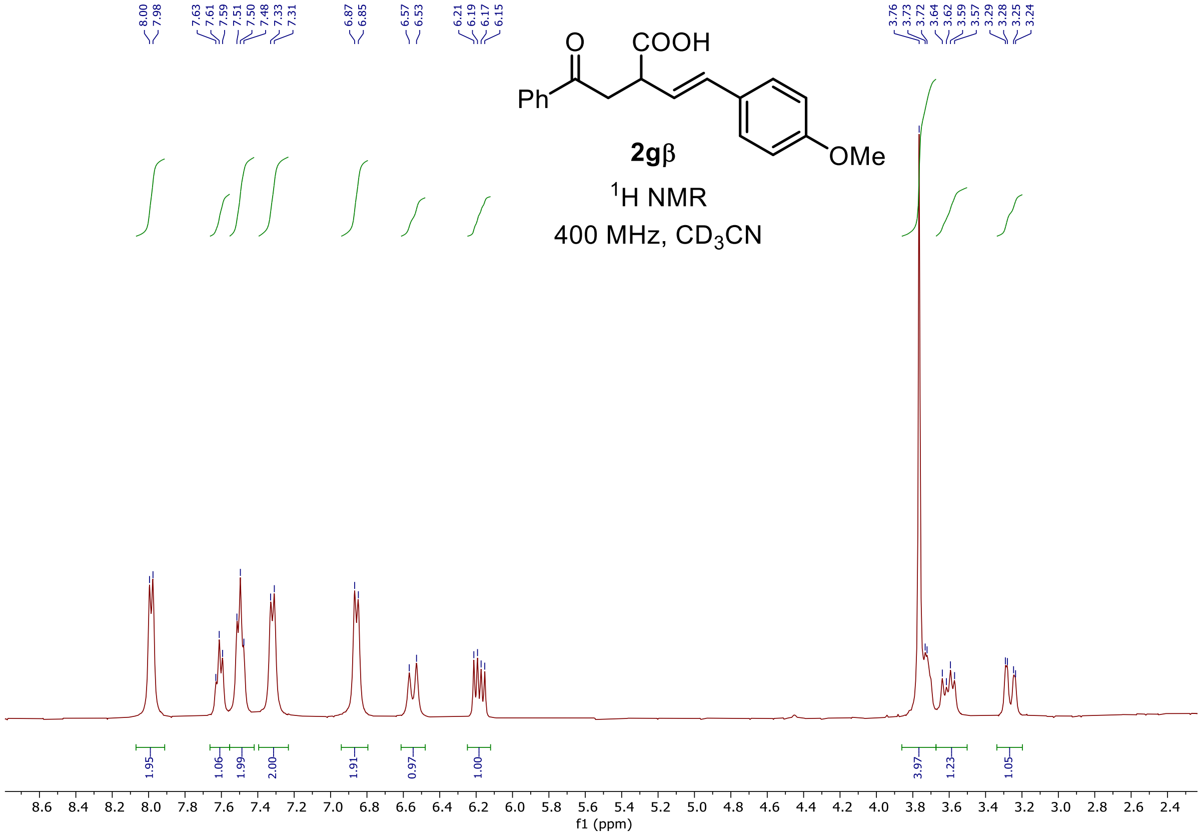
8.00
7.98
7.63
7.61
7.59
7.51
7.50
7.48
7.33
7.31
6.87
6.85
6.57
6.53
6.21
6.19
6.17
6.15
3.76
3.73
3.72
3.64
3.62
3.59
3.57
3.29
3.28
3.25
3.24
O
COOH
Ph
OMe
2gβ
1H NMR
400 MHz, CD3CN
1.95
1.06
1.99
2.00
1.91
0.97
1.00
3.97
1.23
1.05
8.6 8.4 8.2 8.0 7.8 7.6 7.4 7.2 7.0 6.8 6.6 6.4 6.2 6.0 5.8 5.6 5.4 5.2 5.0 4.8 4.6 4.4 4.2 4.0 3.8 3.6 3.4 3.2 3.0 2.8 2.6 2.4
f1 (ppm)

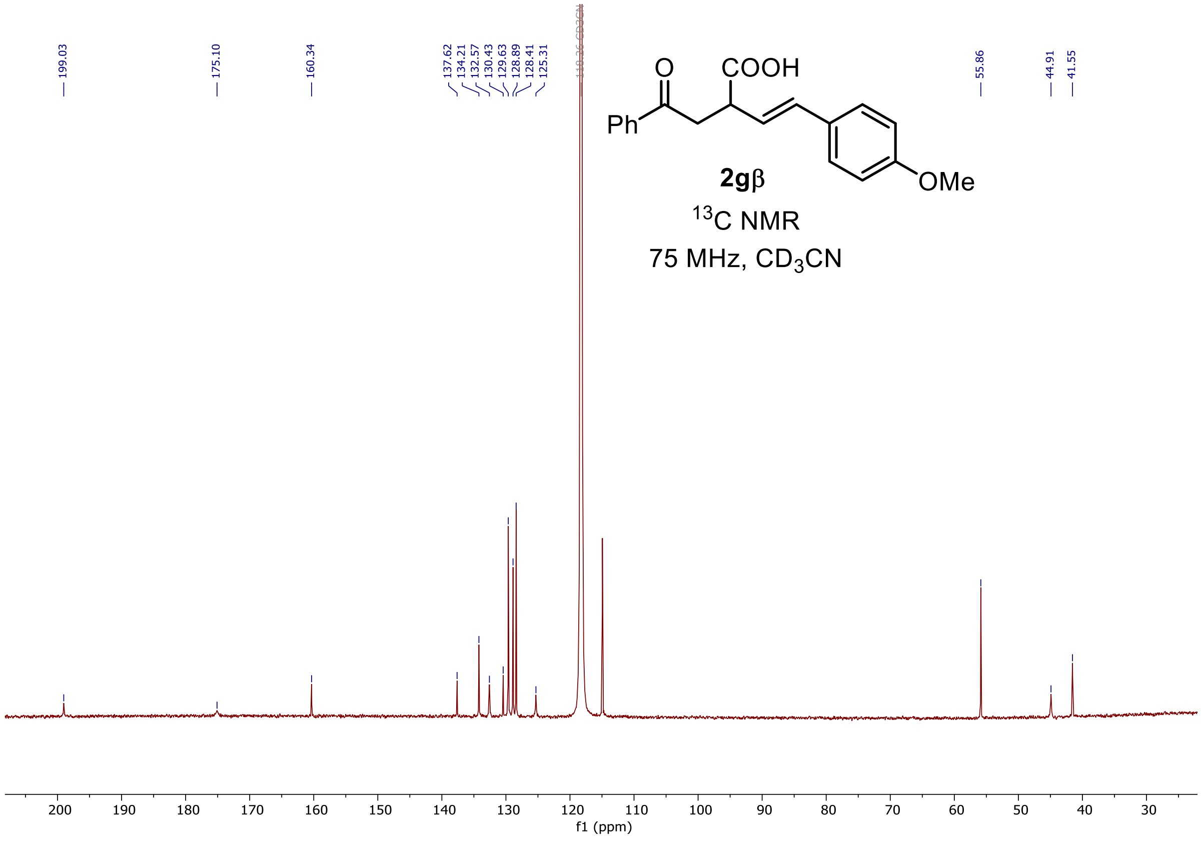
199.03
175.10
160.34
137.62
134.21
132.57
130.43
129.63
128.89
128.41
125.31
55.86
44.91
41.55
O
COOH
Ph
OMe
2gβ
13C NMR
75 MHz, CD3CN
200 190 180 170 160 150 140 130 120 110 100 90 80 70 60 50 40 30
f1 (ppm)

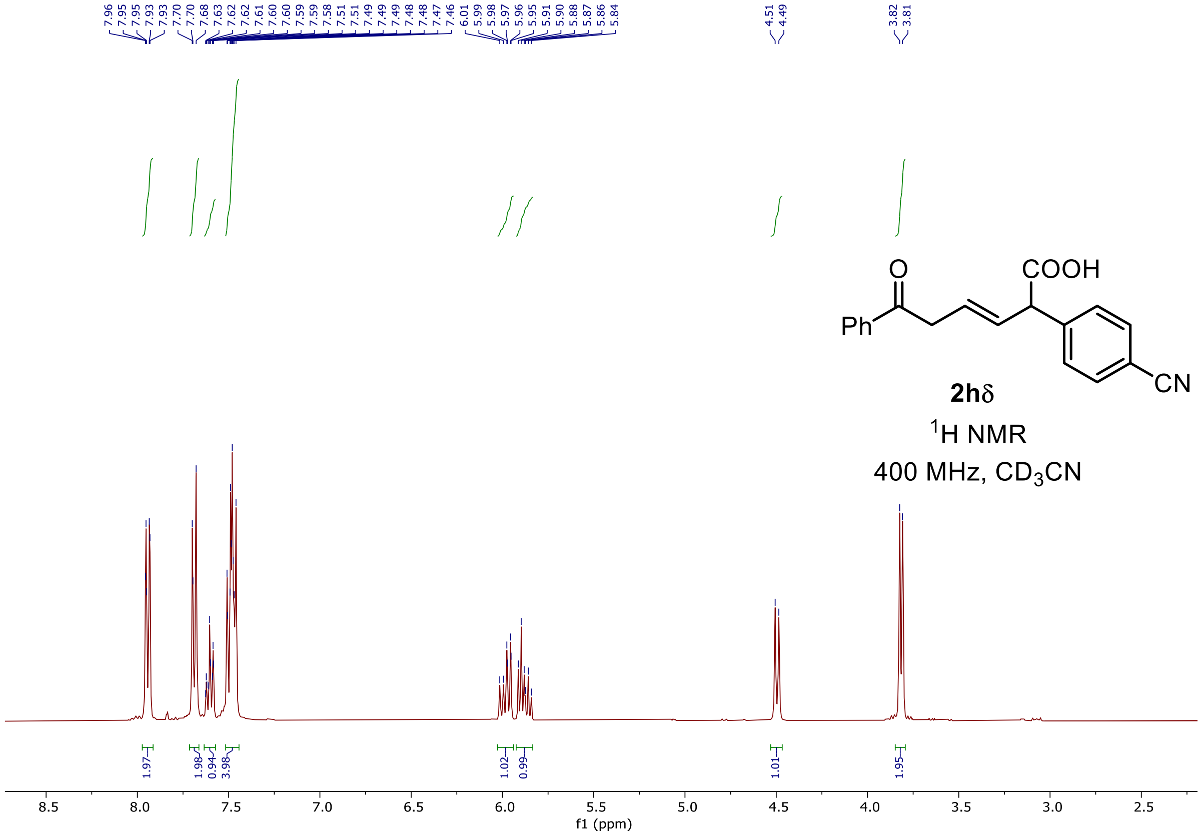
7.96 7.95 7.95 7.93 7.93 7.70 7.68 7.63 7.62 7.62 7.61 7.60 7.60 7.59 7.59 7.58 7.51 7.51 7.49 7.49 7.49 7.48 7.48 7.47 7.46 6.01 5.99 5.98 5.97 5.96 5.95 5.91 5.90 5.88 5.87 5.86 5.84
4.51 4.49
3.82 3.81
O
COOH
Ph
CN
2hδ
1H NMR
400 MHz, CD3CN
1.97 1.98 0.94 3.98 1.02 0.99 1.01 1.95
8.5 8.0 7.5 7.0 6.5 6.0 5.5 5.0 4.5 4.0 3.5 3.0 2.5
f1 (ppm)

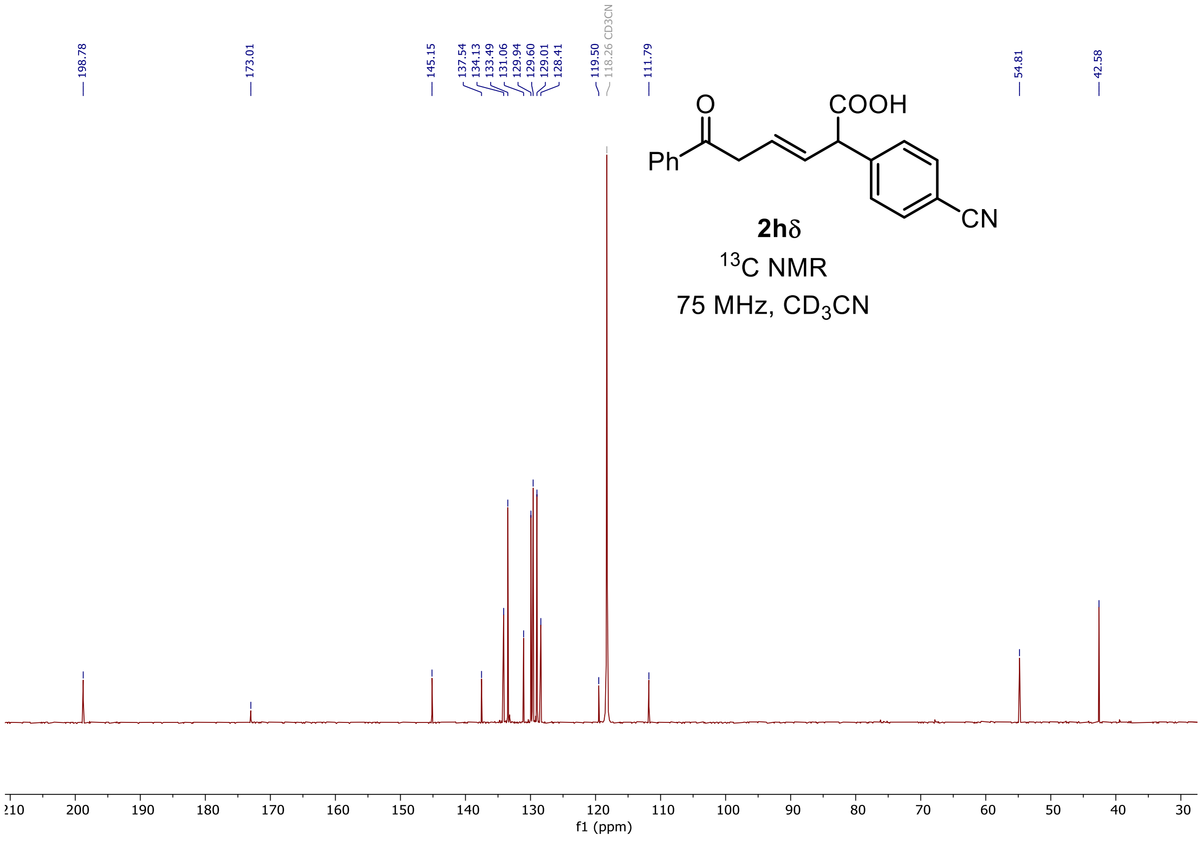
198.78
173.01
145.15 137.54 134.13 133.49 131.06 129.94 129.60 129.01 128.41
119.50 118.26 CD3CN
111.79
54.81
42.58
O
COOH
Ph
CN
2hδ
13C NMR
75 MHz, CD3CN
210 200 190 180 170 160 150 140 130 120 110 100 90 80 70 60 50 40 30
f1 (ppm)

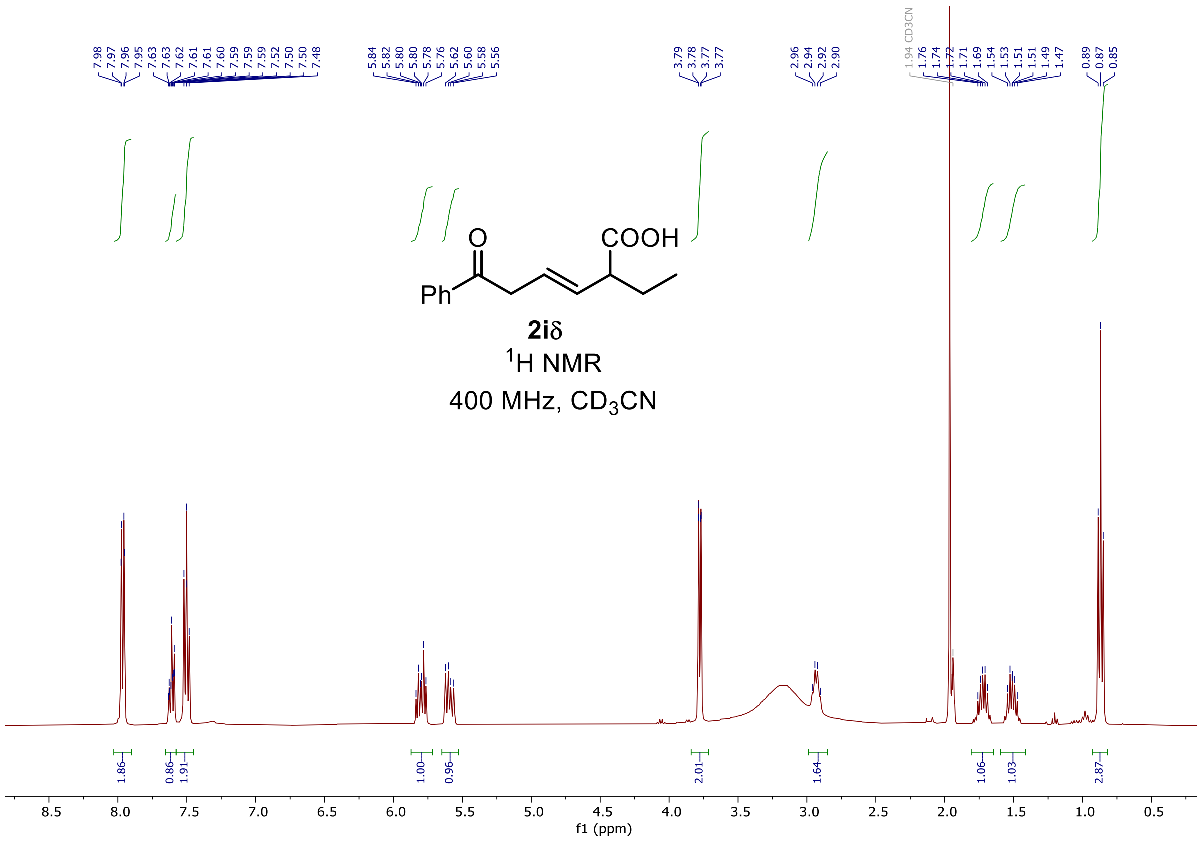

2iδ
^{1}H NMR
400 MHz, CD_3CN
f1 (ppm)

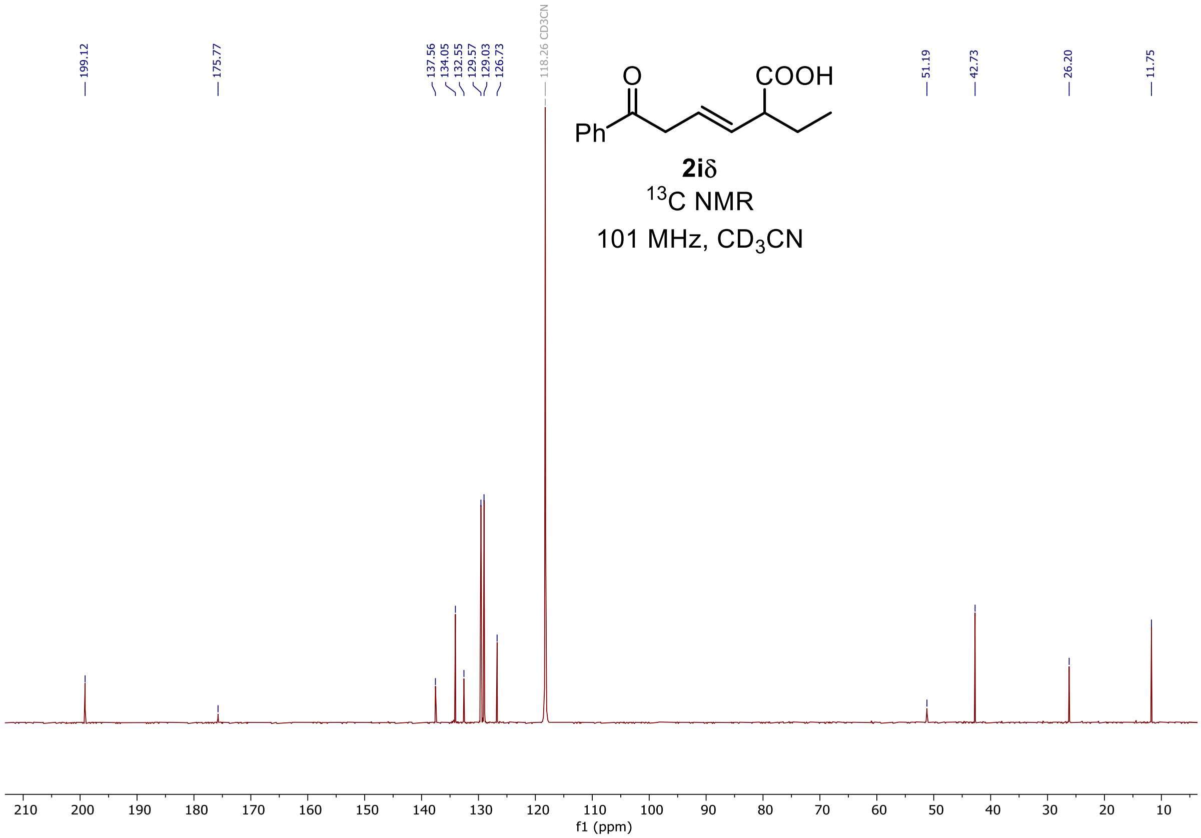

2iδ
^{13}C NMR
101 MHz, CD_3CN
f1 (ppm)

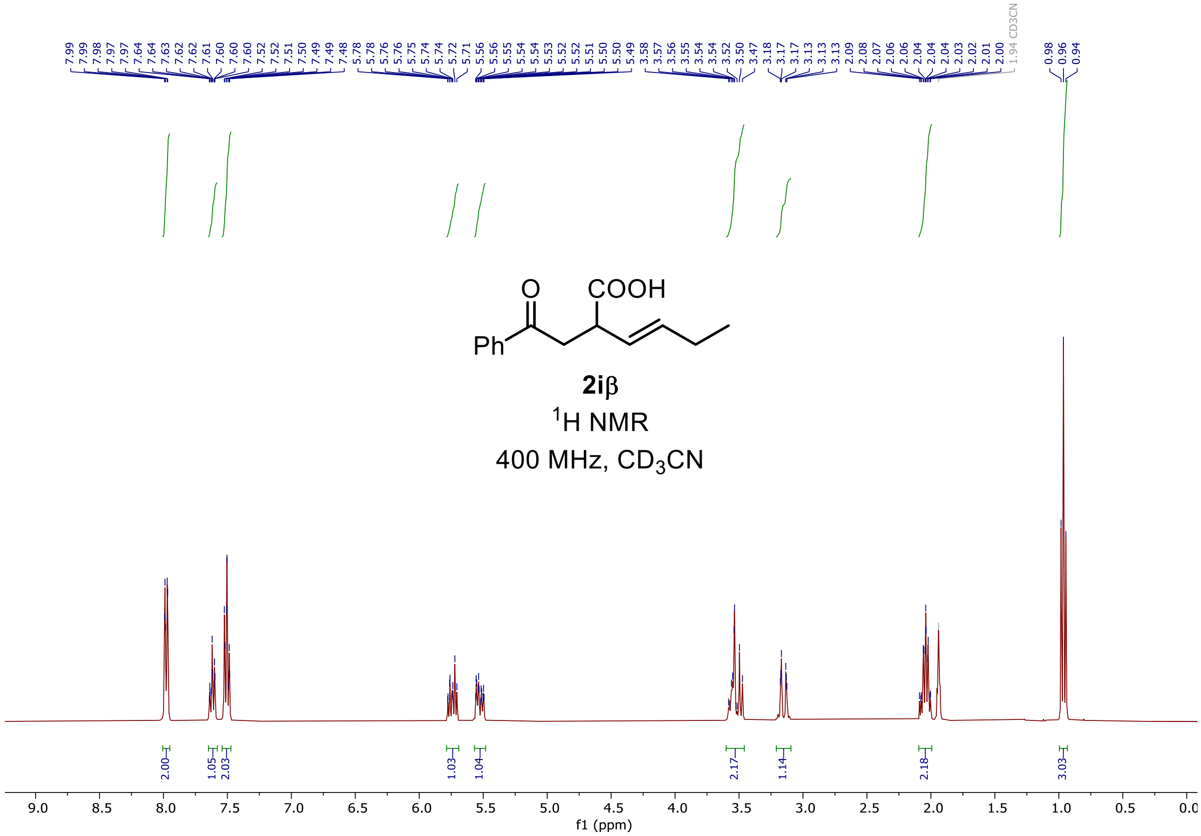
COOH
Ph
2iβ
1H NMR
400 MHz, CD3CN
f1 (ppm)

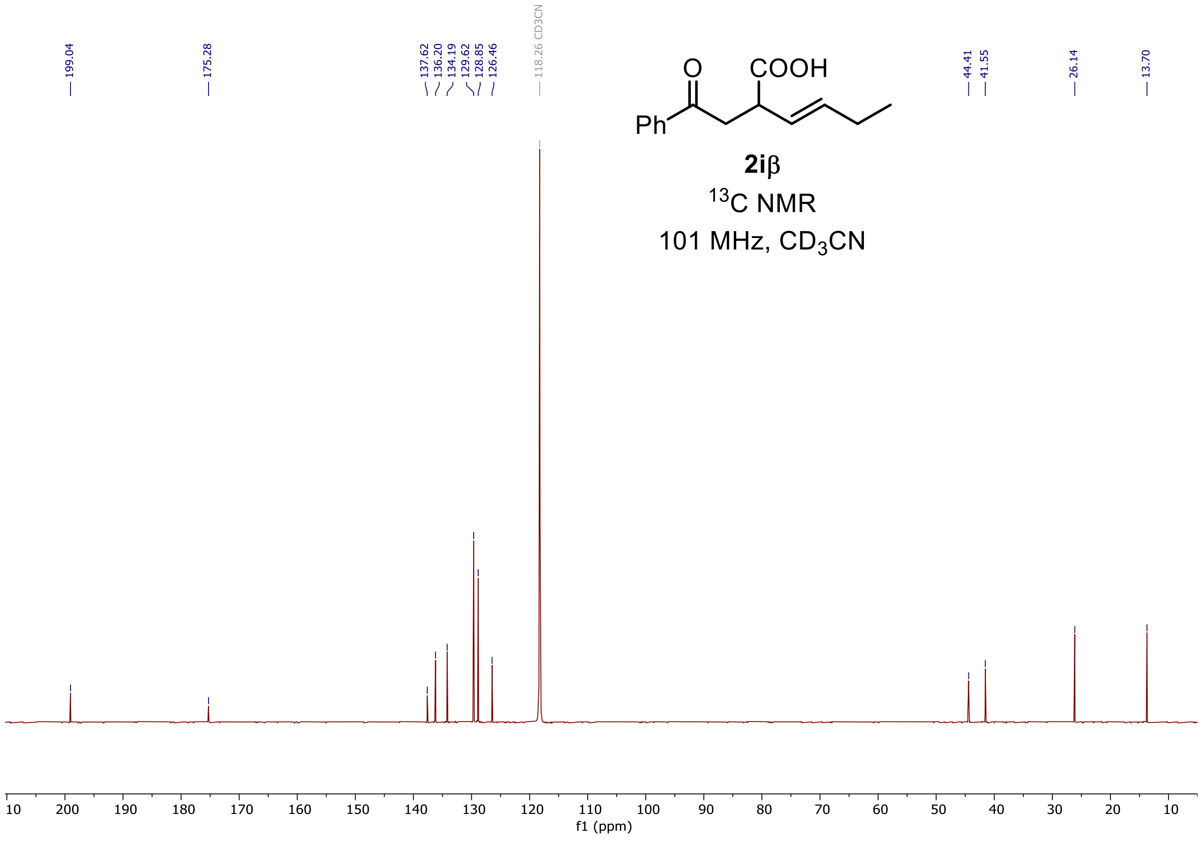
COOH
Ph
2iβ
13C NMR
101 MHz, CD3CN
f1 (ppm)

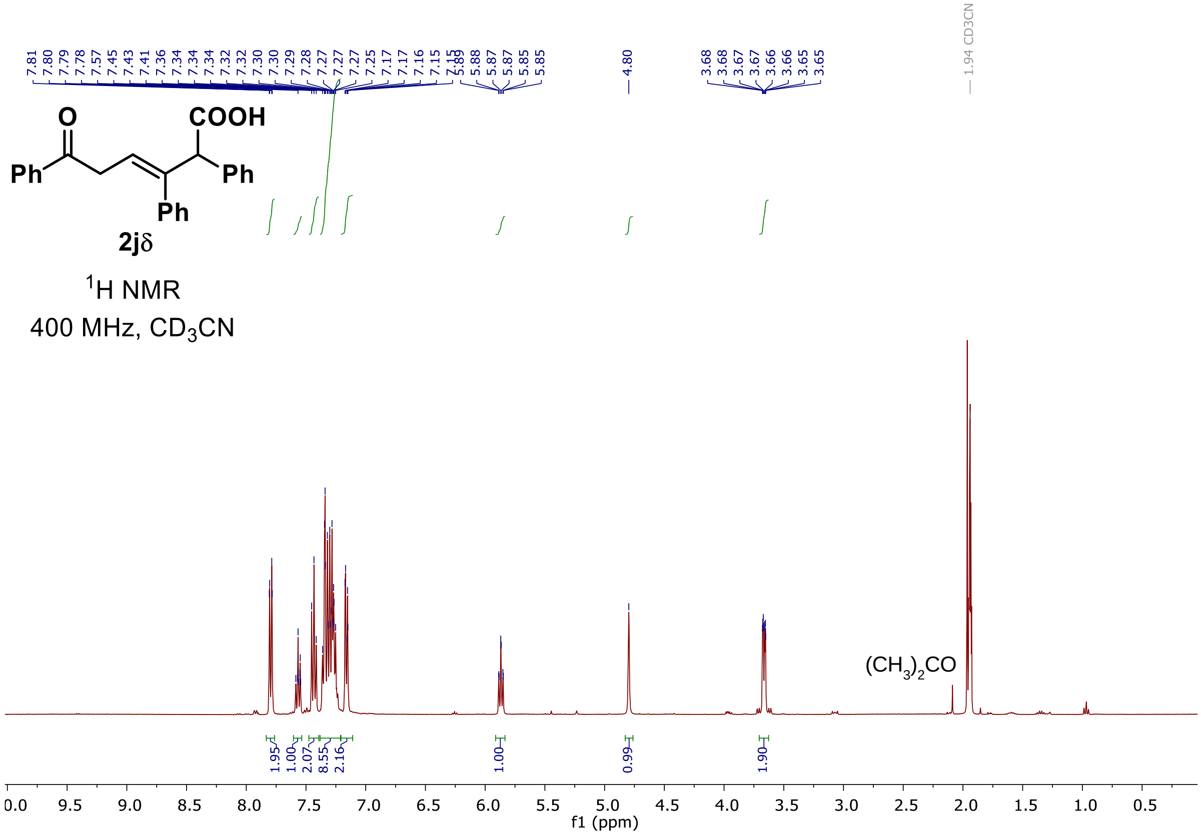
7.81 7.80 7.79 7.78 7.57 7.45 7.43 7.41 7.36 7.34 7.34 7.34 7.32 7.32 7.30 7.30 7.29 7.28 7.27 7.27 7.27 7.25 7.17 7.17 7.16 7.15 7.15 5.89 5.88 5.87 5.87 5.85 5.85
4.80
3.68 3.68 3.67 3.67 3.66 3.66 3.65 3.65
1.94 CD3CN
O
COOH
Ph
Ph
Ph
2jδ
^{1}H NMR
400 MHz, CD_3CN
$(CH_3)_2CO$
1.95 1.00 2.07 8.55 2.16
1.00
0.99
1.90
0.0 9.5 9.0 8.5 8.0 7.5 7.0 6.5 6.0 5.5 5.0 4.5 4.0 3.5 3.0 2.5 2.0 1.5 1.0 0.5
f1 (ppm)

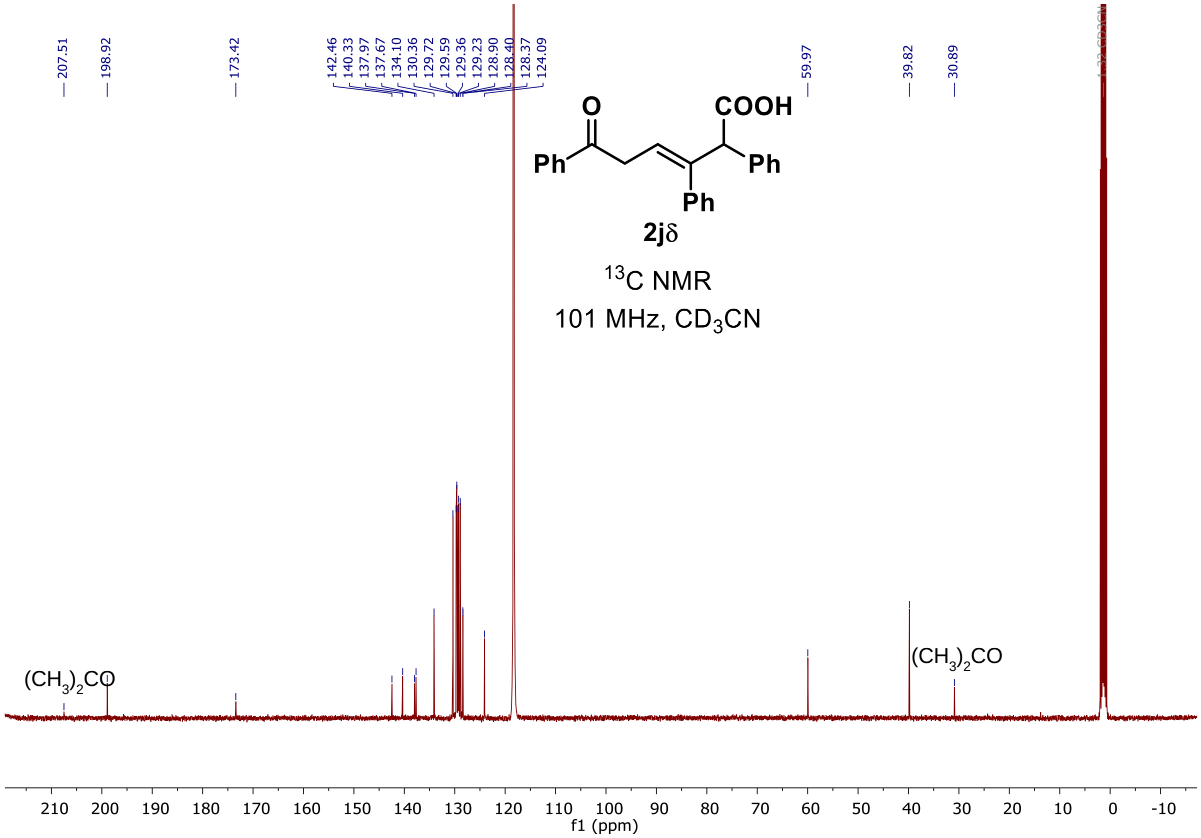
207.51
198.92
173.42
142.46 140.33 137.97 137.67 134.10 130.36 129.72 129.59 129.36 129.23 128.90 128.40 128.37 124.09
59.97
39.82
30.89
O
COOH
Ph
Ph
Ph
2jδ
^{13}C NMR
101 MHz, CD_3CN
$(CH_3)_2CO$
$(CH_3)_2CO$
210 200 190 180 170 160 150 140 130 120 110 100 90 80 70 60 50 40 30 20 10 0 -10
f1 (ppm)

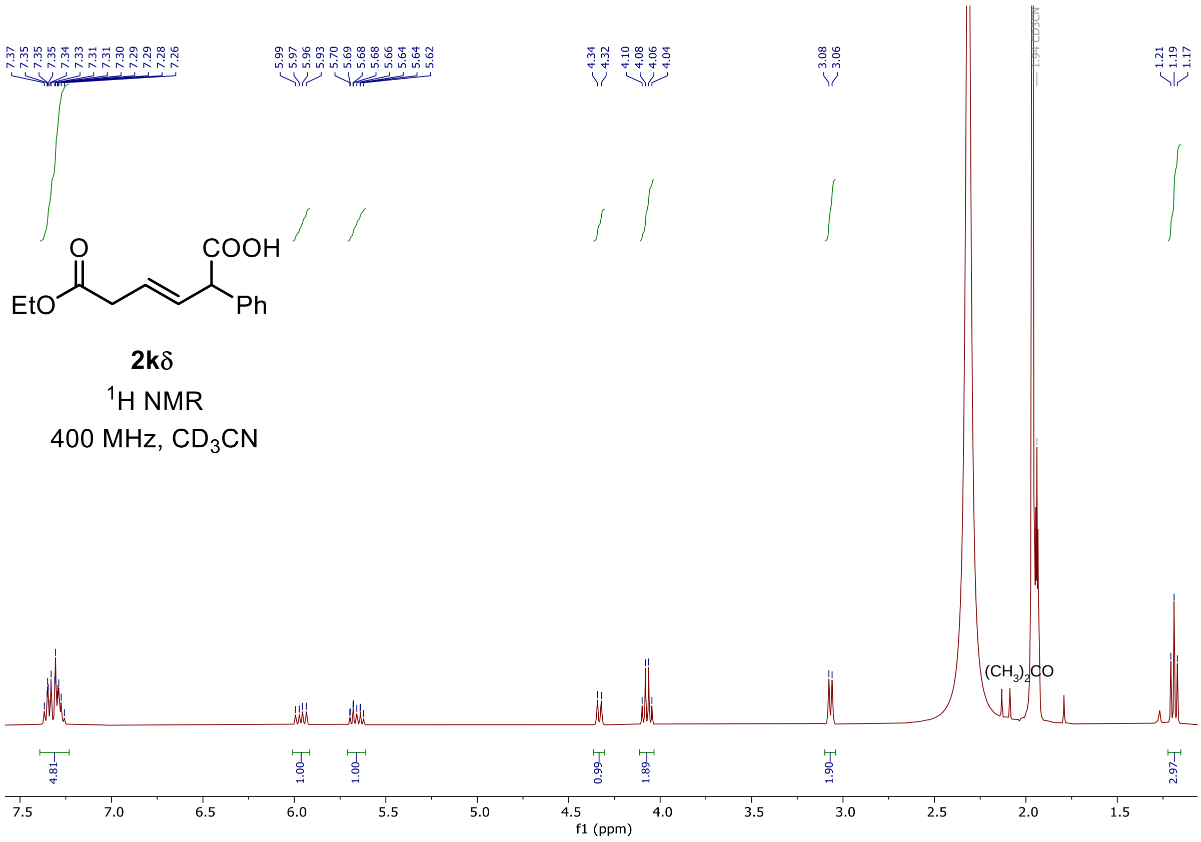
2kδ
^{1}H NMR
400 MHz, CD_3CN
$(CH_3)_2CO$
f1 (ppm)

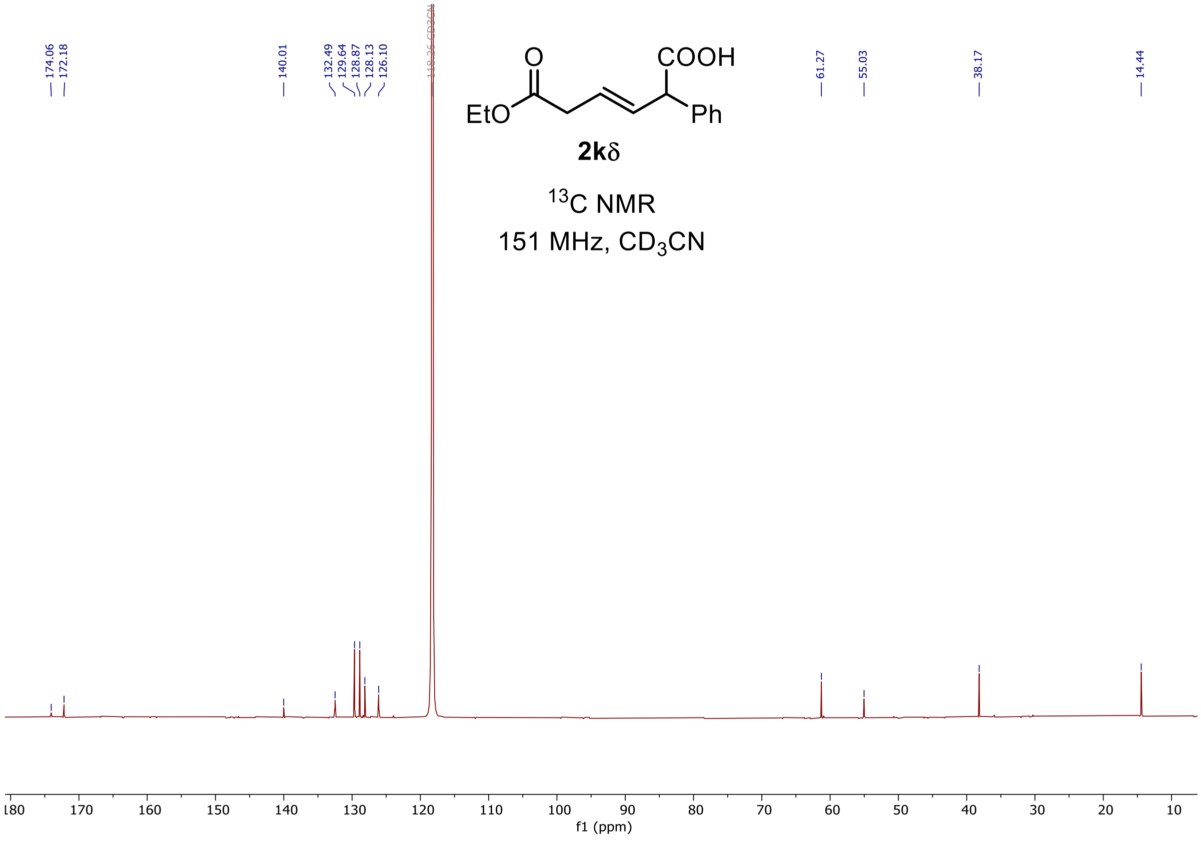
2kδ
^{13}C NMR
151 MHz, CD_3CN
f1 (ppm)

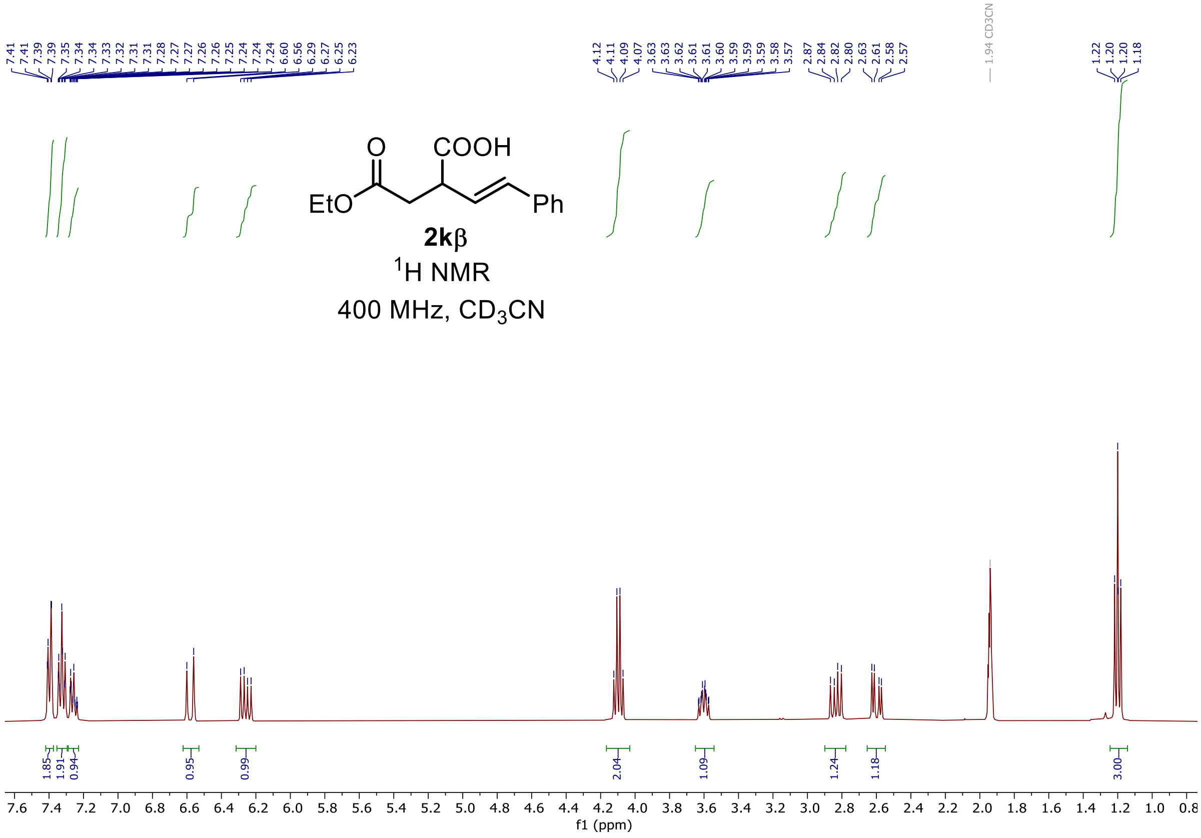
COOH
EtO
Ph
2kβ
1H NMR
400 MHz, CD3CN
f1 (ppm)

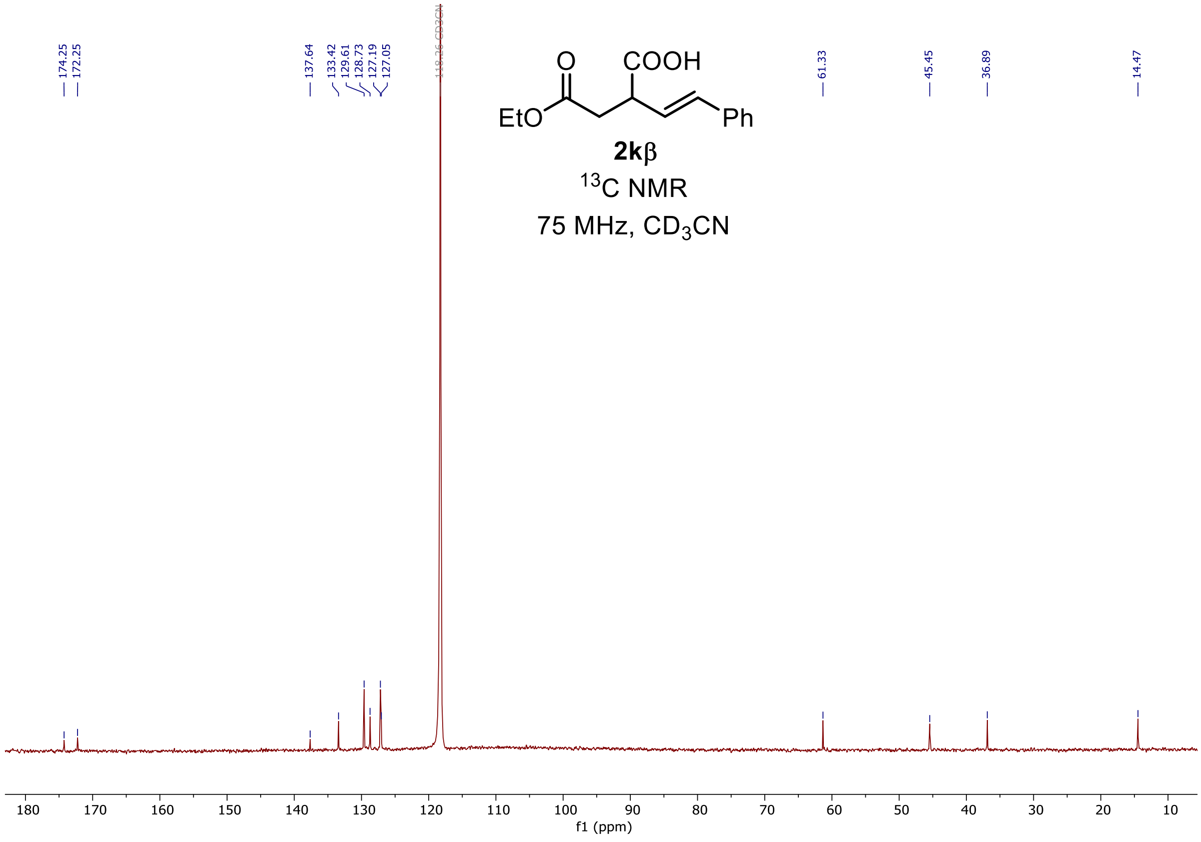
COOH
EtO
Ph
2kβ
13C NMR
75 MHz, CD3CN
f1 (ppm)

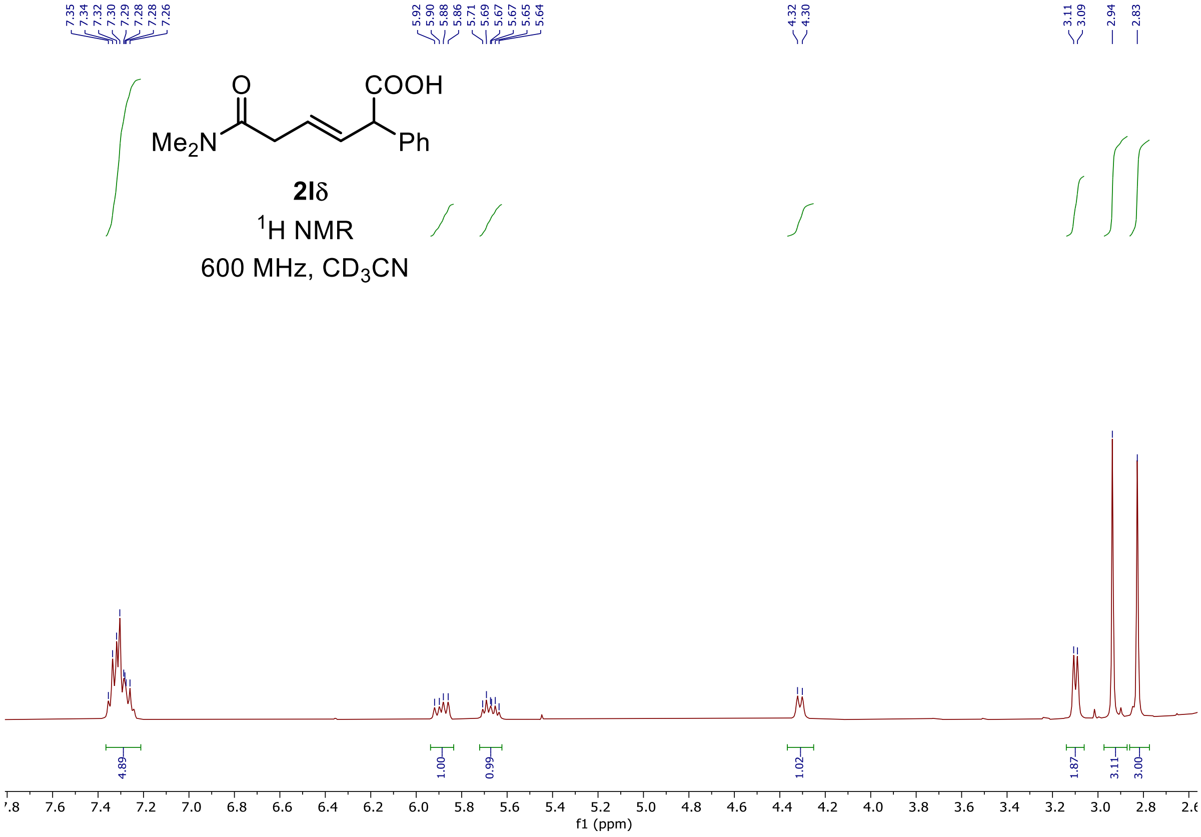
7.35 7.34 7.32 7.30 7.29 7.28 7.28 7.26
5.92 5.90 5.88 5.86 5.71 5.69 5.67 5.67 5.65 5.64
4.32 4.30
3.11 3.09 2.94 2.83
COOH
Me2N
Ph
2lδ
1H NMR
600 MHz, CD3CN
4.89
1.00
0.99
1.02
1.87
3.11
3.00
f1 (ppm)

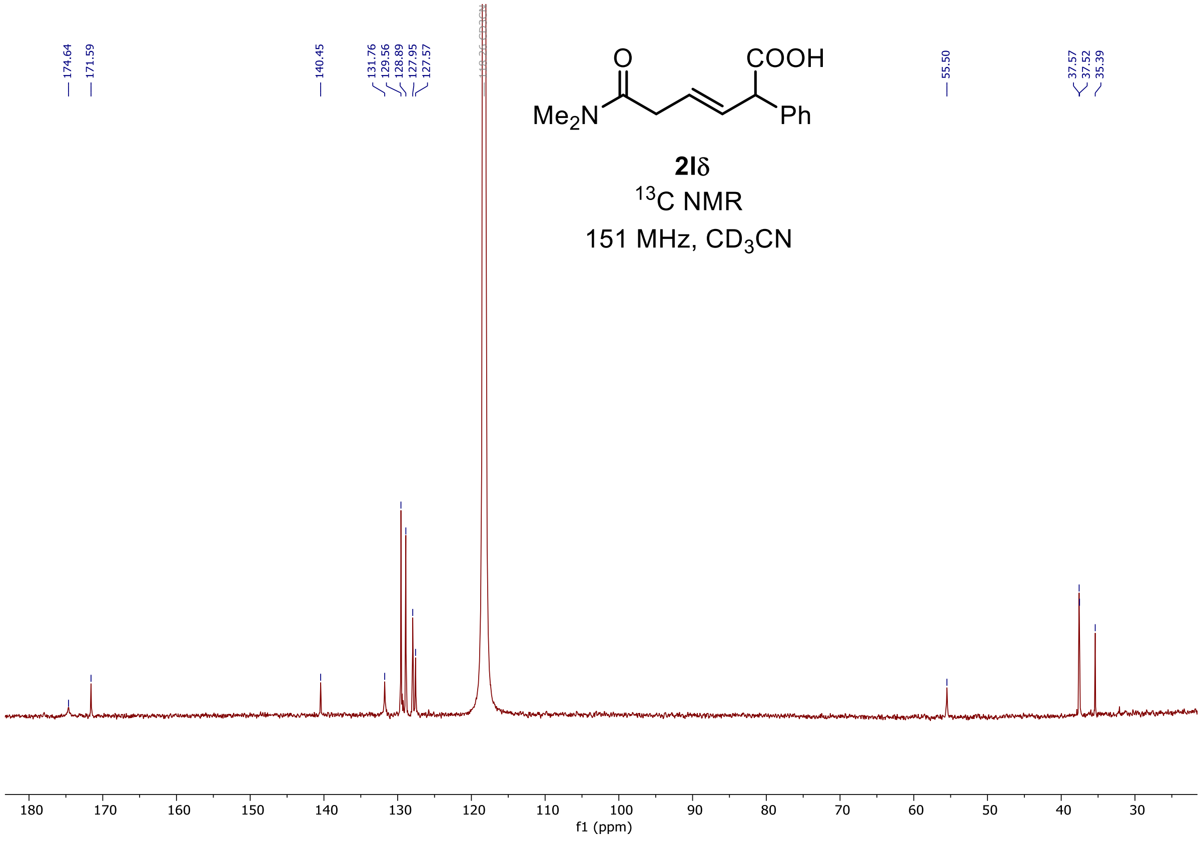
174.64 171.59
140.45
131.76 129.56 128.89 127.95 127.57
55.50
37.57 37.52 35.39
COOH
Me2N
Ph
2lδ
13C NMR
151 MHz, CD3CN
f1 (ppm)

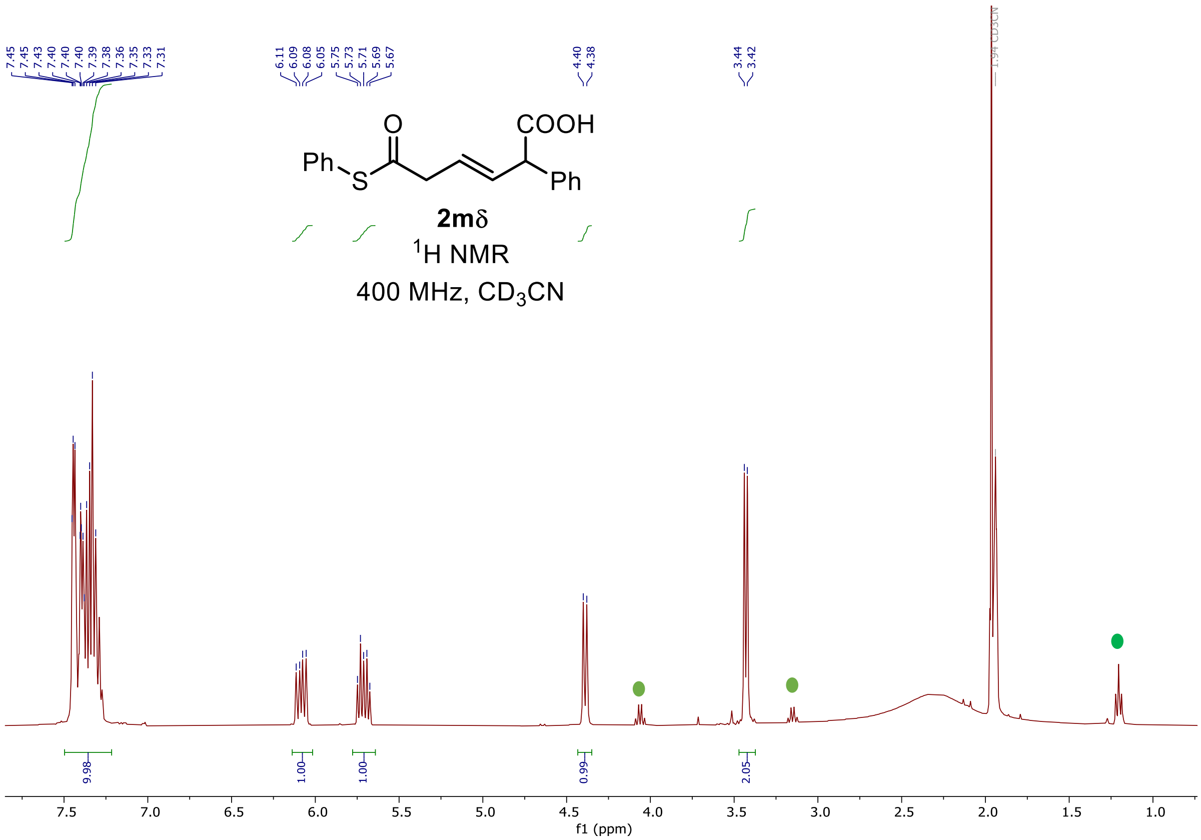

The green spots indicate signals of residual TEA$^+$.

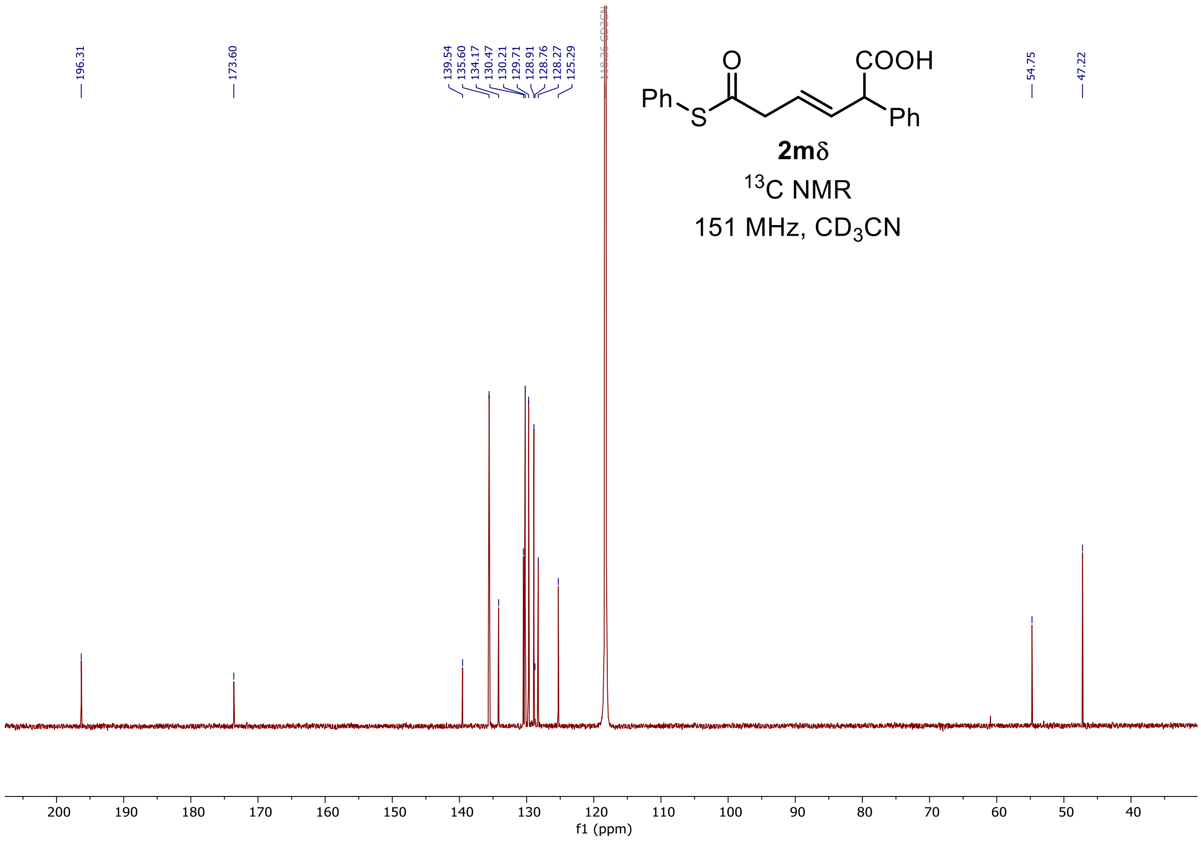
196.31
173.60
139.54
135.60
134.17
130.47
130.21
129.71
128.91
128.76
128.27
125.29
118.26 CD3CN
54.75
47.22
Ph
S
O
COOH
Ph
2mδ
13C NMR
151 MHz, CD3CN
200
190
180
170
160
150
140
130
120
110
100
90
80
70
60
50
40
f1 (ppm)

L. REFERENCES

(1) Gagne, R. R.; Koval., C. A.; Lisensky, G. C. Ferrocene as an Internal Standard for Electrochemical Measurements. *Inorg Chem* **1980**, *19* (9), 2854–2855.

(2) Li, Z.; Mayer, R. J.; Ofial, A. R.; Mayr, H. From Carbodiimides to Carbon Dioxide: Quantification of the Electrophilic Reactivities of Heteroallenes. *J Am Chem Soc* **2020**, *142* (18), 8383–8402. https://doi.org/10.1021/jacs.0c01960.

(3) Harvey, J. N.; Himo, F.; Maseras, F.; Perrin, L. Scope and Challenge of Computational Methods for Studying Mechanism and Reactivity in Homogeneous Catalysis. *ACS Catal* **2019**, *9* (8), 6803–6813. https://doi.org/10.1021/acscatal.9b01537.

(4) Isse, A. A.; Gennaro, A. Absolute Potential of the Standard Hydrogen Electrode and the Problem of Interconversion of Potentials in Different Solvents. *Journal of Physical Chemistry B* **2010**, *114* (23), 7894–7899. https://doi.org/10.1021/jp100402x.

(5) Paul, A.; Borrelli, R.; Bouyanfif, H.; Gottis, S.; Sauvage, F. Tunable Redox Potential, Optical Properties, and Enhanced Stability of Modified Ferrocene-Based Complexes. *ACS Omega* **2019**, *4* (12), 14780–14789. https://doi.org/10.1021/acsomega.9b01341.

(6) Yang, X. Y.; Tay, W. S.; Li, Y.; Pullarkat, S. A.; Leung, P. H. Asymmetric 1,4-Conjugate Addition of Diarylphosphines to α,β,γ,δ-Unsaturated Ketones Catalyzed by Transition-Metal Pincer Complexes. *Organometallics* **2015**, *34* (20), 5196–5201. https://doi.org/10.1021/acs.organomet.5b00787.

(7) Zhou, Y.; Shi, Y.; Torker, S.; Hoveyda, A. H. S N 2″-Selective and Enantioselective Substitution with Unsaturated Organoboron Compounds and Catalyzed by a Sulfonate-Containing NHC-Cu Complex. *J Am Chem Soc* **2018**, *140* (48), 16842–16854. https://doi.org/10.1021/jacs.8b10885.

(8) Guo, Y.; Kootstra, J.; Harutyunyan, S. R. Catalytic Regio- and Enantioselective Alkylation of Conjugated Dienyl Amides. *Angewandte Chemie - International Edition* **2018**, *57* (41), 13547–13550. https://doi.org/10.1002/anie.201808392.

(9) Shi, Y.; Chen, L.; Gao, Q.; Li, J.; Guo, Y.; Fan, B. Application of Oxazaborolidine Catalysts (CBS) on Enantioselective 1,4-Addition of Diarylphosphine Oxides to α,β-Unsaturated Thioesters. *Org Lett* **2023**, *25* (35), 6495–6500. https://doi.org/10.1021/acs.orglett.3c02138.

(10) Belmessieri, D.; Morrill, L. C.; Simal, C.; Slawin, A. M. Z.; Smith, A. D. Organocatalytic Functionalization of Carboxylic Acids: Isothiourea- Catalyzed Asymmetric Intra- and Intermolecular Michael Addition-Lactonizations. *J Am Chem Soc* **2011**, *133* (8), 2714–2720. https://doi.org/10.1021/ja109975c.

(11) Knölker, H. J.; Baum, G.; Foitzik, N.; Goesmann, H.; Gonser, P.; Jones, P. G.; Röttele, H. Synthesis, Molecular Structure, Fluxional Behavior, and Tricarbonyliron Transfer

Reactions of (H4-1-Azabuta-1,3-Diene)Tricarbonyliron Complexes. *Eur J Inorg Chem* **1998**, No. 7, 993–1007.

(12) Jones, B. T.; García-Cárceles, J.; Caiger, L.; Hazelden, I. R.; Lewis, R. J.; Langer, T.; Bower, J. F. Complex Polyheterocycles and the Stereochemical Reassignment of Pileamartine A via Aza-Heck Triggered Aryl C-H Functionalization Cascades. *J Am Chem Soc* **2021**, *143* (38), 15593–15598. https://doi.org/10.1021/jacs.1c08615.

(13) Pinto, D. C. G. A.; Silva, A. M. S.; Lévai, A.; Cavaleiro, José´ A. S. Patonay, T.; Elguero, J. Synthesis of 3-Benzoyl-4-Styryl-2-Pyrazolines and Their Oxidation to the Corresponding Pyrazoles. *European J Org Chem* **2000**, 2593-2599.

(14) Li, C.; Li, M.; Zhong, W.; Jin, Y.; Li, J.; Wu, W.; Jiang, H. Palladium-Catalyzed Oxidative Allylation of Sulfoxonium Ylides: Regioselective Synthesis of Conjugated Dienones. *Org Lett* **2019**, *21* (4), 872–875. https://doi.org/10.1021/acs.orglett.8b03606.

(15) Allen, J. V.; Bergeron, S.; Griffiths, M. J.; Mukherjee, S.; Roberts, S. M.; Williamson, N. M.; Wu, L. E. Juliá-Colonna Asymmetric Epoxidation Reactions under Non-Aqueous Conditions: Rapid, Highly Regio- and Stereo-Selective Transformations Using a Cheap, Recyclable Catalyst. *J Chem Soc Perkin 1* **1998**, No. 19, 3171–3179. https://doi.org/10.1039/a805407j.

(16) Armstrong, A.; Pullin, R. D. C.; Jenner, C. R.; Scutt, J. N. Amine-Promoted Synthesis of Vinyl Aziridines. *Journal of Organic Chemistry* **2010**, *75* (10), 3499–3502. https://doi.org/10.1021/jo100407s.

(17) Li, C.; Lu, W.; Lu, B.; Li, W.; Xie, X.; Zhang, Z. Ru-Catalyzed Chemo- And Enantioselective Hydrogenation of 2,4-Pentadien-1-Ones: Synthesis of Chiral 2,4-Pentadien-1-Ols. *Journal of Organic Chemistry* **2019**, *84* (24), 16086–16094. https://doi.org/10.1021/acs.joc.9b02576.

(18) Demidoff, F. C.; Caleffi, G. S.; Figueiredo, M.; Costa, P. R. R. Ru(II)-Catalyzed Asymmetric Transfer Hydrogenation of Chalcones in Water: Application to the Enantioselective Synthesis of Flavans BW683C and Tephrowatsin e. *Journal of Organic Chemistry* **2022**, *87* (21), 14208–14222. https://doi.org/10.1021/acs.joc.2c01733.

(19) Chen, Y. Z.; Wang, N.; Hou, Z. R.; Zhou, X. L.; Li, X.; Gao, F.; Jiang, T. Palladium-Catalyzed Stereoselective Ring-Opening Reaction of Aryl Cyclopropyl Ketones. *Org Biomol Chem* **2022**, *20* (27), 5412–5415. https://doi.org/10.1039/d2ob00719c.

(20) Oliva, C. G.; Silva, A. M. S.; Resende, D. I. S. P.; Paz, F. A. A.; Cavaleiro, J. A. S. Highly Enantioselective 1, 4-Michael Additions of Nucleophiles to Unsaturated Aryl Ketones with Organocatalysis by Bifunctional Cinchona Alkaloids. *European J Org Chem* **2010**, No. 18, 3449–3458. https://doi.org/10.1002/ejoc.201000273.

(21) Kolb, B.; Dos Santos, D. S.; Krause, S.; Zens, A.; Laschat, S. Sequential Hydrozirconation/Pd-Catalyzed Cross Coupling of Acyl Chlorides towards Conjugated (2E,4E)-Dienones. *Beilstein Journal of Organic Chemistry* **2023**, *19*, 176–185. https://doi.org/10.3762/bjoc.19.17.

(22) Sun, X.; Gu, P.; Qin, J.; Su, Y. Rhodium-Catalysed Diastereo- And Enantioselective Cyclopropanation of α-Boryl Styrenes. *Chemical Communications* **2020**, *56* (82), 12379–12382. https://doi.org/10.1039/d0cc02549f.

(23) Isse, A. A.; Gennaro, A. Mechanism of the Electrochemical Carboxylation of Aromatic Ketones in Dimethylformamide. *Collect Czechoslov Chem Commun* **2003**, *68* (8), 1379–1394. https://doi.org/10.1135/cccc20031379.

(24) Tyssee, D. A.; Baizer, M. M. Electrocarboxylation. II Electrocarboxylative Dimerization and Cyclization. *Journal of Organic Chemistry* **1974**, *39* (19), 2823–2828.
